# Genome-Wide Study of the GATL Gene Family in *Gossypium hirsutum* L. Reveals that *GhGATL* Genes Act on Pectin Synthesis to Regulate Plant Growth and Fiber Elongation

**DOI:** 10.3390/genes11010064

**Published:** 2020-01-06

**Authors:** Lei Zheng, Huanhuan Wu, Ghulam Qanmber, Faiza Ali, Lingling Wang, Zhao Liu, Daoqian Yu, Qian Wang, Aixia Xu, Zuoren Yang

**Affiliations:** 1 State Key Laboratory of Cotton Biology, Institute of Cotton Research of the Chinese Academy of Agricultural Sciences, Anyang 455000, China; zhengleiwangyi@126.com (L.Z.);; 2 College of Agronomy, Northwest A & F University, Yangling 712100, China; xuaixia2013@163.com

**Keywords:** *GhGATL* gene family, *Gossypium hirsutum* L., evolution, pectin, stem diameter, fiber

## Abstract

Pectin is a major polysaccharide component that promotes plant growth and fiber elongation in cotton. In previous studies, the galacturonosyltransferase-like (GATL) gene family has been shown to be involved in pectin synthesis. However, few studies have been performed on cotton *GATL* genes. Here, a total of 33, 17, and 16 *GATL* genes were respectively identified in *Gossypium hirsutum*, *Gossypium raimondii*, and *Gossypium arboreum*. In multiple plant species, phylogenetic analysis divided *GATL* genes into five groups named GATL-a to GATL-e, and the number of groups was found to gradually change over evolution. Whole genome duplication (WGD) and segmental duplication played a significant role in the expansion of the GATL gene family in *G. hirsutum*. Selection pressure analyses revealed that GATL-a and GATL-b groups underwent a great positive selection pressure during evolution. Moreover, the expression patterns revealed that most of highly expressed *GhGATL* genes belong to GATL-a and GATL-b groups, which have more segmental duplications and larger positive selection value, suggesting that these genes may play an important role in the evolution of cotton plants. We overexpressed *GhGATL2*, *GhGATL9*, *GhGATL12,* and *GhGATL15* in *Arabidopsis* and silenced the *GhGATL15* gene in cotton through a virus induced gene silencing assay (VIGS). The transgenic and VIGS lines showed significant differences in stem diameter, epidermal hair length, stamen length, seed size, and fiber length than the control plant. In addition, the pectin content test proved that the pectin was significantly increased in the transgenic lines and reduced in VIGS plants, demonstrating that *GhGATL* genes have similar functions and act on the pectin synthesis to regulate plant growth and fiber elongation. In summary, we performed a comprehensive analysis of *GhGATL* genes in *G. hirsutum* including evolution, structure and function, in order to better understand *GhGATL* genes in cotton for further studies.

## 1. Introduction

The plant cell wall is a complex macromolecular structure mostly composed of polysaccharides. It has important roles in plants, including defending against pathogens, providing structural support to cells, and regulating cell to cell communication [1,2]. Pectin is an essential polysaccharides within the cell wall that makes connections between cellulose, hemicelluloses, and proteoglycans in the plant [3]. The presence of pectin promotes cell wall deposition and assembly, which can regulate cell expansion [4,5]. Homogalacturonan (HG), a linear homopolymer of 1,4-linked-D-galactopyranosyluronic acid (GalA) [6,7], is the most abundant pectic domain and an important polysaccharide that contributes to the structure and mechanical strength of the plant cell wall [8].

The glycosyltransferases (GT) family of carbohydrate-active enzymes (CAZys) constitute a large family of enzymes that are involved in the biosynthesis of oligosaccharides, polysaccharides, and glycoconjugates [9]. Recently, GT family 8 (GT8) was confirmed to catalyze the transfer of diverse sugars onto lipo-oligosaccharide, protein, inositol, oligosaccharide or polysaccharide acceptors, involved in the synthesis of the cell wall pectic polysaccharide in higher plants [10,11]. Galacturonosyltransferase (GAUT) is the largest family of the GT8, a kind of alpha-1,4-galacturonosyltransferase (GalAT) that can transfer galacturonic acid onto the pectic polysaccharide and synthesizes HG [12]. In *Arabidopsis*, the *Arabidopsis thaliana* GAUT1-related (AtGAUT1-related) gene family was divided into four related clades of *GAUT* and *GAUT-like* genes by sequence alignment and phylogenetic analysis, and those genes are distinct from the other members of GT8 [10,13].

The function of some *GAUT* genes has been reported in *Arabidopsis*. *GAUT1* was the first gene verified to be involved in pectin synthesis, and it was confirmed to catalyze the transfer of GalA onto HG and exhibited the characteristic of Golgi-localized type-II membrane protein [12]. The *GAUT1* anchor in the Golgi requires association with *GAUT7* to form a *GAUT1:GAUT7* heteromeric enzyme complex, and the *GAUT1:GAUT7* complex shows soluble properties and catalyzes elongation of HG products in vitro [14,15]. Further, mutation in *GAUT8* (*QUA1*) showed a dwarf phenotype and reduced in cell adhesion, and *GAUT8* gene had been identified to affect HG and xylan biosynthesis [16,17,18,19]. The *GAUT12* is abundantly expressed in xylem vessels and interfascicular fiber cells. The *irx8/gaut12* mutants showed collapse of xylem vessels and the xylan and HG significantly decreased in the vascular tissues [20,21]. The transcription of *GAUT13* and *GAUT14* is strongest in pollen tubes, which are essential for pollen tube growth and possibly participate in pectin biosynthesis of the pollen tube wall [22,23]. In addition, Caffall et al. systematically analyzed the composition of somatic cell wall glycosyl residues in 26 homozygous Transfer DNA (T-DNA) insertion mutants for 13 of the 15 *Arabidopsis GAUT* genes. The results showed that *GAUT6, 8, 9, 10, 11, 12, 13, 14* mutants significantly changed the composition of the glycosyl residues in cell walls compared to wild type, suggesting that mutations of these *GAUT* genes affect the biosynthesis of pectin and xylan [24].

The galacturonosyltransferase-like (GATL) gene family was identified as closely related to the GAUT family based on the conservation of amino acid motifs [10]. In *Arabidopsis*, *AtGATL5* is expressed in all plant tissues and the T-DNA insertion mutant of *AtGATL5* caused seed coat epidermal cell defects by affecting mucilage synthesis and cell adhesion [25]. In woody plants, *PdGATL1.1* and *PdGATL1.2* were shown to function in xylan synthesis, and may be involved in the synthesis of other cell wall polymers [26]. The GATL genes family has been systematically analyzed in *Arabidopsis*, in terms of gene structure, genomic organization, protein topology, phylogeny, evolutionary history, and expression pattern, and three *AtGATL* genes have been identified to encode proteins involved in cell wall biosynthesis [27]. In addition, seven members of the GATL gene family have been identified in the rice and their preliminary evolutionary and structural analyses have been carried out [28]. Nevertheless, the detailed biological functions of most of the *GATL* genes still remain elusive.

Cotton is the most important fiber crop and cotton fiber is an important raw material for the textile industry. In cotton fiber, cell walls not only define plant morphogenesis, but also define the industrially important fiber quality parameters. Pectin can provide structural support in primary walls, and influence secondary wall formation in fibers and woody tissues [29,30]. In previous studies, the cotton *GT8* gene was found highly express in the zero days post-anthesis (DPA) ovules and its transcripts were regulated by the *GbPDF1* gene, which was confirmed to participate in fiber initiation and early elongation [31]. The cotton *GT8* gene was highly identical to *Arabidopsis AtGATL1* [32], demonstrating that the GATL family proteins may also be related to fiber growth in cotton. However, a comprehensive analysis of the GATL gene family in cotton remains elusive. Thereby, it was imperative to identify GATL family members and analyze their functions in cotton. In this study, we identified *GATL* genes in three cotton species and 12 other plant species in order to determine their phylogenetic relationships. Furthermore, gene structure, chromosome location, segmental duplication, and expression patterns of the GhGATL gene family were analyzed. Moreover, for functional analysis we overexpressed the *GhGATL* genes in *Arabidopsis*, and silenced them by virus induced gene silencing (VIGS) in cotton.

## 2. Materials and Methods

### 2.1. Identification of GATL Genes

The genome sequences of *Gossypium hirsutum* (NAU, v1.1 and HAU, v1.1), *Gossypium raimondii* (JGI, v1.0), and *Gossypium arboreum* (BJI, v1.0) were downloaded from COTTONGEN (www.cottongen.org). The amino acid sequences of *GATLs* from *Arabidopsis thaliana* were acquired from The Arabidopsis Information Resource, version 10 (TAIR 10) (http://www.arabidopsis.org). The genome sequences of grape (*Vitis vinifera Genoscope* v2.1), poplar (*Populus trichocarpa* v3.1), cacao (*Theobroma cacao* v1.1), peach (*Prunus persica* v2.1), maize (*Zea mays* v4), apple (*Malus domestica* v1.1), rice (*Oryza sativa* v7.0), sorghum (*Sorghum bicolor* v3.1.1), soybean (*Glycine max* v1.1), and two types of moss (*Sphagnum fallax* v0.5 and *Physcornitrella patens* v3.3) were obtained from JGI (https://phytozome.jgi.doe.gov/pz/portal.html). We downloaded the Hidden Markov Model (HMM) of the Glyco_transf_8 (PF01501) domain from Pfam (http://pfam.xfam.org/) and used it to build a new HMM with *Arabidopsis* GATL protein sequences as template with the hmmbuild program (http://hmmer.org/). Then we used the new HMM of GATL as a query to search the *G. hirsutum* protein database for candidate sequences, employing the hmmsearch program (http://hmmer.org/). The GATL gene families in *G. arboreum*, *G. raimondii,* and other species were analyzed as described above. The blastp program was also used to search *GATL* genes and compared with the result of hmmsearch.

### 2.2. Conserved Sequence and Phylogenetic Analysis

The ClustalW program was used for multiple-sequence alignments (build-in MEGA 7.0) [33]. First, we aligned the full length GATL protein sequences of all 15 plant species, and the multiple sequence alignment results were used to construct a phylogenetic tree by neighbor-joining (NJ). The best substitution model was tested with the method of maximum likelihood (ML) (build-in MEGA 7.0). The minimum-evolution method (ME) was also used to construct a phylogenetic tree for validating the NJ tree. Methods and parameters were used as in the Jones–Taylor–Thornton (JTT) model, gamma distributed rates (G) and gamma parameter 1. The bootstrap method was used with 1000 replicates.

### 2.3. Chromosomal Location and Synteny Analysis

The *GhGATL* gene loci were extracted from the annotated gff3 file using a Perl script, and their locations were displayed on the chromosome using MapChart [34]. For synteny analysis, Basic Local Alignment Search Tool (BLAST) was used to align the entire protein sequences of *G. hirsutum* with each other by an e-value of 1 × 10^−5^. The MCSCAN software was used to analyze the results of BLASTP, identify collinearity blocks across the whole genome and classify the duplicate type of *GhGATL* genes [35]. The collinearity pairs belonging to the GhGATL gene family were extracted and used to draw a synteny map by CIRCOS software [36].

### 2.4. Gene Structure Analysis and Protein Motif Analysis

The Clustal W program was used for protein sequences of *G. hirsutum*, and NJ was used to construct the phylogenetic tree with the method and the parameters as described above. The conserved motifs of the GhGATL gene family were determined by the online program MEME (http://meme-suite.org) [37] and displayed by TBtools [38]. The online tool GSDS 2.0 was used to display the exon positions acquired from the gff3 file using a Perl script [39].

### 2.5. Selective Pressure Analysis

The Clustal ×2.0 was used to align amino acid sequences from *GATL* homologous genes and the results were converted to PAML format using the EasyCodeML convertor program [40]. The MEGA 7.0 was used to build the tree file from the alignment result and formed a Newick format file. The selective pressure was estimated using the EasyCodeML. A branch model was used in this current study, the free-ratio model and two-ratio model were used to determine the ratio of nonsynonymous to synonymous substitution rates (ω) among branches of the tree. The adaptability of these two models was assessed by likelihood ratio test (LRT).

### 2.6. Transcriptome Data Analysis and Gene Expression Heatmap

The raw RNA-Seq data of *G. hirsutum* TM-1 were downloaded from the National Center for Biotechnology Information (NCBI) Gene Expression repository under the accession number PRJNA248163 (https://www.ncbi.nlm.nih.gov/bioproject/PRJNA248163/). TopHat and cufflinks were used to map reads and analyze gene expression levels, FPKM (fragments per kilobase million values) were used to normalize gene expression levels. The expression pattern of *GhGATLs* were visualized using MeV_4_9_0 software (open source genomic analysis software; www.tm4.org).

### 2.7. Plasmid Construction and Plant Transformation

To generate over expression plant lines, the coding regions of four *GhGATL* genes were amplified from complementary DNA (cDNA) of the CRI24 accession obtained from the Institute of Cotton Research of the Chinese Academy of Agricultural Sciences and inserted into the vector pCambia2300 containing the CAULIFLOWER MOSAIC VIRUS (CaMV) 35S constitutive promoter. The fusion genes were introduced into *Arabidopsis* wild-type plants (Columbia-0 ecotype) via the floral dip method [41] with *Agrobacterium tumefaciens* (strain GV3101). Positive lines were selected on ½ MS medium plates containing kanamycin (50 mg/L), kept at 4 °C for 3 days in darkness to break seed dormancy and then shifted to a growth incubator at 22 °C under 16 h light and 8 h dark cycle. The positive lines were further verified by using PCR and the same method was used until Transgenic 4 (T4) homozygous generations. Collected phenotypic data of the transgenic and wild type (WT) plants at different developmental stages.

### 2.8. Pectin Staining

Paraffin sections were made from the stem of *Arabidopsis*, and the sections were placed on a glass slide. After dewaxing and rehydration, sections were stained with 0.02% ruthenium red for 4 min, then decolorized with water for 30 min at room temperature [42]. Staining was observed under microscope (LEICA DM6B).

### 2.9. Pectin Content Determination

Pectin content was determined using the pectin content kit from Sino Best Biological Technology Company. The sample and distilled water were mixed at a ratio of 1:5 (mass:volume), fully grinded and the supernatant was collected directly after centrifugation (8000× *g* for 10 min) at 37 °C. The carbazole reagent and standard reagent were preheated at 37 °C, then the supernatant, carbazole reagent, standard reagent and concentrated sulfuric acid were mixed in the following proportions: A1 (blank tube), 100 μL ddH_2_O, 100 μL carbazole reagent, and 800 μL concentrated sulfuric acid; A2 (standard tube), 100 μL standard reagent, 100 μL carbazole reagent, and 800 μL concentrated sulfuric acid; A3 (control tube), 100 μL supernatant, 100 μL ddH_2_O, and 800 μL concentrated sulfuric acid; A4 (measuring tube), 100 μL supernatant, 100 μL carbazole reagent, and 800 μL concentrated sulfuric acid. The mixtures were put in a 95 °C water bath for 5 min and detected with a microplate reader. The pectin content was calculated using the following formula:

Pectin content (mg/g) = (C × V1) × (A4 – A3)/(A2 – A1)/W × dilution multiple,

where C is the concentration of the standard tube (0.05 mg/mL), V1 is sample volume, and W is the fresh weight of the sample (g) [43].

### 2.10. Virus Induced Gene Silencing Assay

Virus induced gene silencing (VIGS) was used to mute *GATL* gene function in plants. An approximately 300-bp coding region of *GhGATL15* was amplified by using CRI24 cDNA as a template and inserted into the pCLCrVA vector. The fusion *GhGATL15* and empty vector (negative control) transferred into *Agrobacterium tumefaciens* (strain GV3101) and respectively mixed with the pCLCrVB (helper vector) strain in ratio of 1:1 (OD600 = 1.5), injected into cotyledons of CRI24 wild-type plants. The plants first were dark cultured for 24 h, then shifted to a growth environment of 22 °C with a 16 h light/8 h dark cycle. For qRT-PCR analysis, samples were collected from at least six uniformly injected plants. Phenotypic variations in the plants were observed at different developmental stages [44].

## 3. Results

### 3.1. Identification of GATL Genes

Ten *Arabidopsis* GATL protein sequences were obtained from TAIR (http://www.arabidopsis.org) and used to build a new GATL HMM with the HMM of the Glyco_transf_8 (PF01501) domain from Pfam (http://pfam.xfam.org/). Thereafter, we queried the GATL HMM to search for the GATL proteins among the obtained protein database, and 33, 17, 16, 7, 10, 8, 8, 12, 8, 18, 10, 6, and 5 genes were confirmed as *GATL* genes in *G. hirsutum*, *G. raimondii*, *G. arboreum*, rice, maize, cacao, grape, poplar, peach, sorghum, apple, *Sphagnum fallax* and *Physcornitrella patens*, respectively (Table S1). The numbers of *GATL* genes in our search results were the same as that identified in rice and *Arabidopsis* in previous studies [27,28]. The number of *GATL* genes in *G. hirsutum* was the sum of *GATL* genes present in *G. arboreum* (AA) and *G. raimondii* (DD), consistent with the established fact that *G. hirsutum* was derived from hybridization of *G. arboreum* (A2) and *G. raimondii* (D5) and their subsequent polyploidization [45,46]. In addition, the Dt-subgenome had one more gene (*GhGATL2*) than the At-subgenome in *G. hirsutum*, consistent with the fact that *G. raimondii* had one more gene (*GrGATL2*) than *G. arboreum*.

To verify the previous results of *GhGATL* genes obtained from the *G. hirsutum* NAU (v1.1) database, the HMM of GATL was used as a query to identify the *GhGATL* genes from the *G. hirsutum* HAU (v1.1) database. We found a slight difference between the two versions (NAU and HAU) of *G. hirsutum* database: in D05 and A05 chromosomes, *G. hirsutum* NAU had one more *GhGATL* gene than *G. hirsutum* HAU, and in D07 chromosome *G. hirsutum* HAU had one more *GhGATL* gene than *G. hirsutum* NAU. We amplified these three *GhGATL* genes using CRI24 cDNA as a template, and the sequencing results showed that these three *GhGATLs* were present in the cotton genome (Figure S1). Besides this, there were two scaffolds which contained *GhGATL* genes that have not been assembled into the chromosome in *G. hirsutum* NAU; we confirmed that one was the same as the *GhGATL* gene in the A02 chromosome of *G. hirsutum* HAU and the other was just a repeat sequence segment in *G. hirsutum* NAU. Finally, the *GhGATL* genes were grouped according to the *Arabidopsis* homologous gene, and sorted the genes in each group based on positions on the chromosome, then given the proposed genes names *GhGATL1* to *GhGATL17* (Table S2), and the same numbers were given to their orthologs in *G.raimondii* and *G. arboreum* (Table S3).

### 3.2. Phylogenetic Analysis of GATL Genes

**Figure 1:**
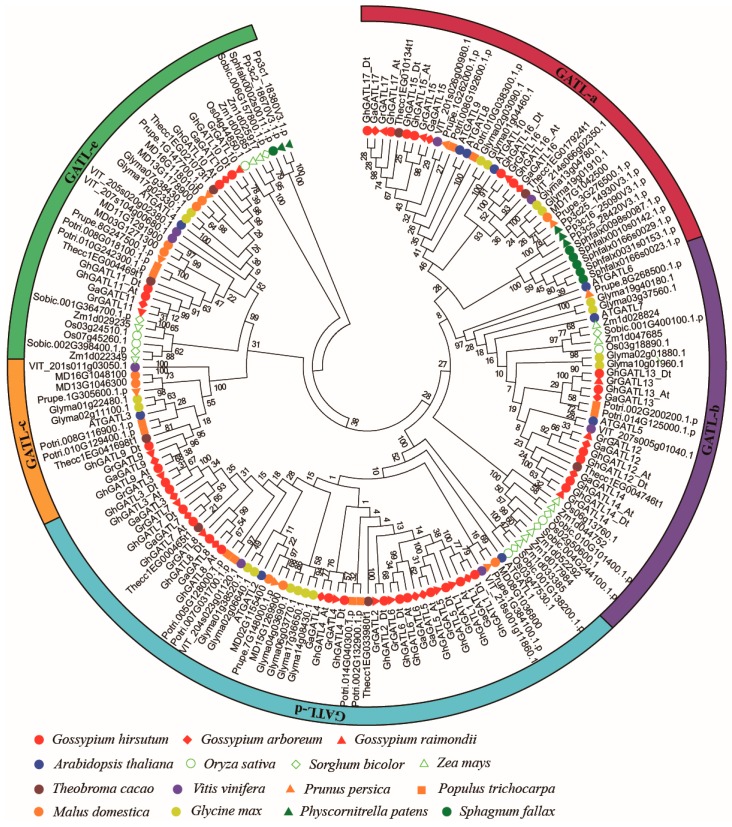
Phylogenetic tree of *GATL* genes from 15 species indicating that *GATL* genes can be divided into five groups. The outer colored circles represent the GATL gene family groups from GATL-a to GATL-e. The prefixes Gh, Ga, Gr, At, Os, Sobic, Zm, Thecc, VIT, Prupe, Potri, MD, Glyma, Pp3c, and Sphfalx, represent *Gossypium hirsutum*, *Gossypium arboreum*, *Gossypium raimondii*, *Arabidopsis thaliana*, *Oryza sativa*, *Sorghum bicolor*, *Zea mays*, *Theobroma cacao*, *Vitis vinifera Genoscope*, *Prunus persica*, *Populus trichocarpa*, *Malus domestica*, *Glycine max*, *Physcornitrella patens,* and *Sphagnum fallax*, respectively. The “At” and “Dt” indicate the A-and D-subgenomes in *G. hirsutum* respectively. The reliability value of nodes was provided using bootstrapping.

To investigate the phylogenetic relationships of *GATL* genes among three cotton species (*G. hirsutum, G. raimondii, and G. arboreum*) and the 12 other species, we constructed a NJ tree using MEGA 7.0 [33]. Like the previous phylogenetic analysis of *GATL* genes in *Arabidopsis*, *GATL* genes from all 15 plant species in this study were divided into five groups from GATL-a to GATL-e (Figure 1) [27]. The ME tree was constructed using MEGA 7.0 to evaluate the accuracy of the NJ tree (Figure S2). Both NJ and ME trees showed almost the same topology, indicating the NJ tree could be used for further analysis. Group GATL-d contained the maximum number of *GATL* genes, group GATL-c contained the minimum number of *GATL* genes, and we found that group GATL-c contained at least one gene from each dicot plant, indicating that group GATL-c has not undergone expansion and the members of this group may have similar biological functions. Only groups GATL-a and GATL-e contained *GATL* genes from mosses of *Physcomitrella patens* and *Sphagnum fallax*; monocots species such as maize, rice, and sorghum had *GATL* genes in only three groups (GATL-b, GATL-d, and GATL-e); and other dicotyledons contained *GATL* genes from four or five groups. These findings suggested that the number of GATL groups may have changed gradually during the evolutionary process.

In addition, cotton *GATL* genes showed a close relationship with cacao *GATL* genes, as their *GATL* genes clustered together with each other in different groups; this is consistent with the fact that the cacao genome is the closest relative of cotton sequenced so far. There are seven to twelve *GATL* genes in most diploid species such as grape, poplar, cacao, maize, sorghum, rice, and apple. Soybean is a diploid crop derived from an ancient tetraploid that has two times the number of *GATL* genes compared to other diploid species [47]. *G. hirsutum* has four times the number of *GATL* genes compared to other diploid species, because *G. hirsutum* is a typical allotetraploid composed from *G. arboreum* and *G. raimondii* [48]. These results showed that the number of *GATL* genes were stable over evolution, but the genes have different duplications or doubling in each subgroup; whole genome duplication (WGD) is the major impetus of *GATL* genes expansion in evolution.

### 3.3. Gene Expansion and Synteny Analysis

**Figure 2:**
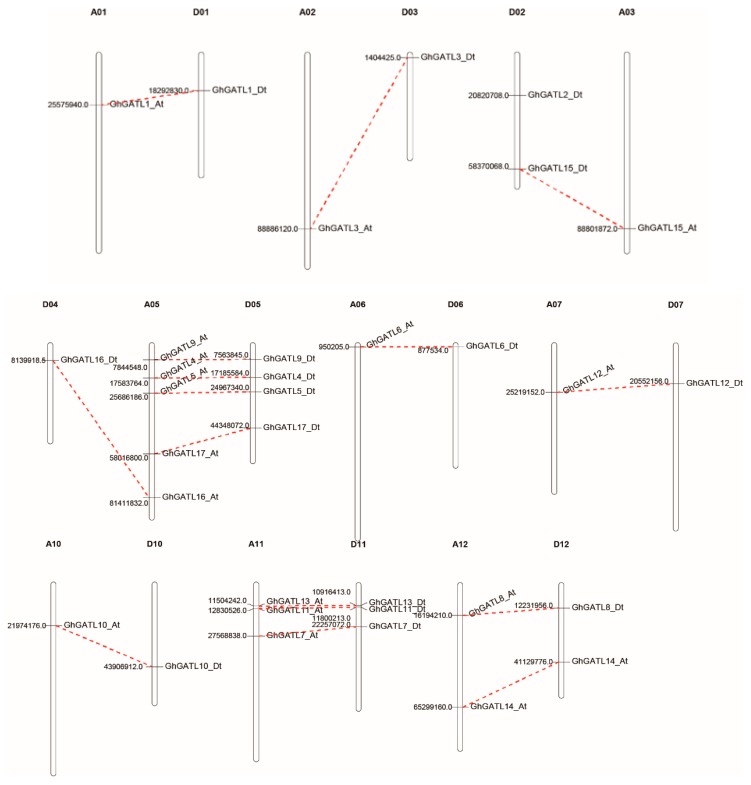
Chromosomal distribution of *GhGATL* genes in *G. hirsutum*. The number represents the median bases number of *GhGATL* genes in the splicing sequence of the chromosome. The red dotted lines link the orthologs genes on the At and Dt subgenomes.

*G. hirsutum* is a typical allotetraploid, which is an ideal material for studying genome polyploidy and its effects [48]. The synteny analysis in *G. hirsutum* was performed to analyze the collinearity relationship of orthologs between the At and Dt subgenomes. The incomplete sequencing in *G. hirsutum* NAU (v1.1) meant that *GhGATL3_At* and *GhGATL12_Dt* were not mapped to a chromosome. Therefore, we localized them in the chromosomes map based on the *G. hirsutum* HAU (v1.1) gff3 file. The synteny analysis results indicated that most of the *GhGATL* loci were significantly conserved between the At and Dt subgenomes. A total of 33 *GhGATL* genes were distributed unevenly among 19 chromosomes and no *GhGATL* gene was found on chromosomes A04, A08, D08, A09, and D09. Two homologous gene pairs of *GhGATL3* (At/Dt) and *GhGATL15* (At/Dt) were located on the same two non-homologous chromosomes of A02/D02 and A03/D03, which showed that translocation might occur between two non-homologous chromosomes after allotetraploid formation. *GhGATL16_Dt* on the D04 chromosomes is homologous with *GhGATL16_At* on the A05 chromosomes and there was not any other *GhGATL* gene on the A04 chromosomes, which might result from possible chromosome fragment translocation from the A05 to D04 (Figure 2).

**Figure 3:**
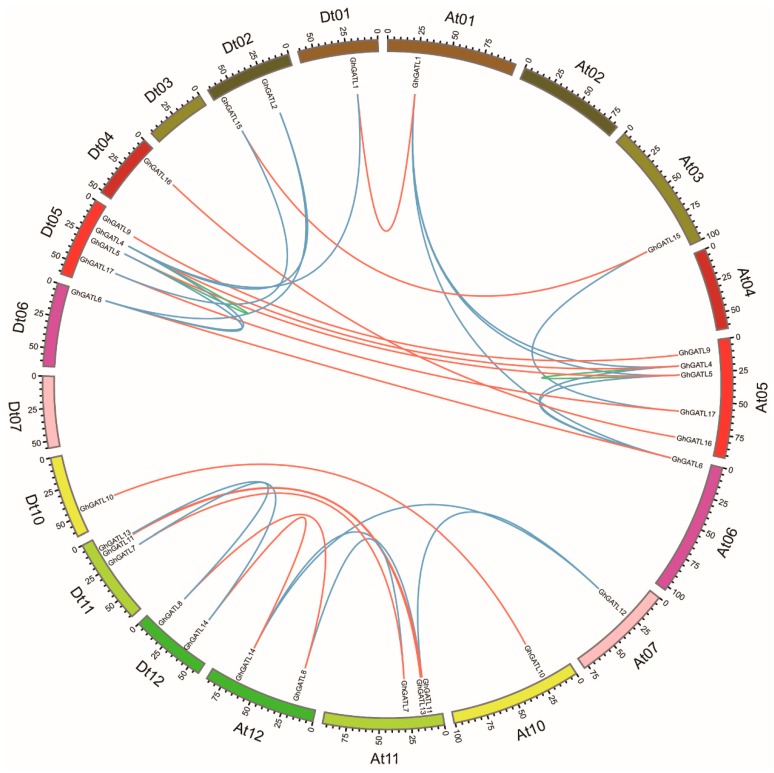
Synteny analyses of *GhGATL* genes in *G. hirsutum*. The orange lines link the orthologous genes from the At and Dt subgenomes. The blue lines link paralogous pairs derived from segmental duplication. The green lines link paralogous pairs derived from segmental duplication in the same chromosomes.

Duplication is the major impetus underlying gene expansion during evolution. Five types of gene duplication may occur in evolution, including singleton, dispersed, tandem, proximal, and segmental duplication. We used MCSCAN to determine the duplicate gene type and collinearity blocks across the whole genome [35]. During our analysis, as *GhGATL12_Dt* could not be found in *G. hirsutum* NAU (v1.1) database, the duplication type was not identified. Many members of the *GhGATL* genes were duplicated in collinearity regions. In particular, chromosome 5 contained four *GhGATL* genes and an abundant collinearity replication relationship with other chromosomes, suggesting that these genes were active during evolution. *GhGATL9_At/Dt* did not have segmental duplication, and *GhGATL9_At/Dt* belonged to the GATL-c group which had the fewest *GATL* genes based on phylogenetic analysis. Among the *GhGATL* genes, there were no tandem or proximal duplications (in the nearby chromosomal region, not in the adjacent region) (Figure 3, Table S4).

In evolution, function mutations may occur after genes duplication, some genes may retain original function, lose their functions, or acquire some novel function [49]. In order to determine the significance of these duplicated genes during the long history of evolution, we used EasyCodeML to identify genes based on different selective pressures, including purifying selection, positive selection, and negative selection [40]. The selection pressure was estimated using a branch model test among different branches of the phylogenetic tree (Table 1). As shown in Table 1, the mean ω values of GATL-a and GATL-b branches were all 999.0, which were significantly larger than 1.0 and background values, suggesting that the branches of GATL-a and GATL-b underwent major positive selection. The mean ω value of GATL-e branches was 2.0, suggesting that it underwent less positive selection. Moreover, the other two branches had the mean ω values smaller than 1.0, suggesting that they underwent purifying selection.

### 3.4. Gene Structure and Motif Analysis

**Figure 4:**
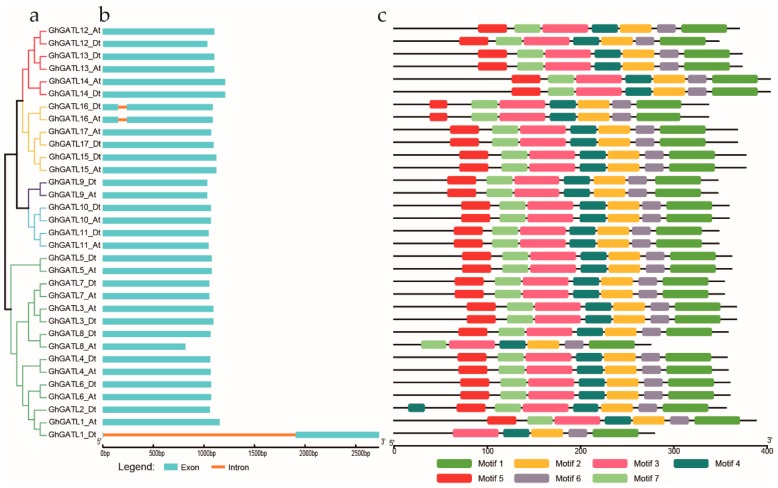
Comparison of the gene structures and motif distribution pattern of *GhGATL* gene in *G. hirsutum*. (**a**) The neighbor-joining (NJ) tree of *G. hirsutum GhGATL* genes. (**b**) The position of exons and introns within *GhGATL* genes. Exons and introns are shown by blue boxes and orange lines, respectively. (**c**) The distribution pattern of predicted motifs in the *GhGATL* genes.

The GATL gene family was identified as closely related to the GAUT family, but GATL protein sequences had some significant differences from GAUTs. For instance, GATL protein sequences were smaller than GAUTs, an identifiable transmembrane domain that is found in almost all GAUTs was lacking in GATL proteins, and most of *GATL* genes lacked introns [11,50]. To further study the structure of *GhGATL* genes in cotton, we analyzed gene structure and conserved domains of *GhGATL* genes. Most of the *GhGATL* genes lacked introns except *GhGATL1_Dt*, *GhGATL16_At*, and *GhGATL16_Dt* (Figure 4). The *GhGATL16_At* and *GhGATL16_Dt* are homologous genes, and both had an 85 base pair (bp) intron in the N-terminal region. Similarly, *GhGATL1_Dt* had a 1901bp intron behind the 7 bp exon in the N-terminal region (Figure 4b). In order to better understand the phylogenetic relationships and structure of *GhGATL* genes, we used MEME to discover the possible motifs within *GhGATL* gene sequences (Table S5). A total of seven motifs were identified in the Glyco_transf_8 (PF01501) domain of *GhGATL* genes except *GhGATL1_Dt* and *GhGATL8_At* which lacked two and one motifs in the N-terminal region of Glyco_transf_8 domain, respectively (Figure 4c). We found that the missing motif did not affect the phylogenetic relationship of *GATL* genes.

### 3.5. Expression Profiles of GhGATL Gene in Different Tissues under Multiple Stresses

**Figure 5:**
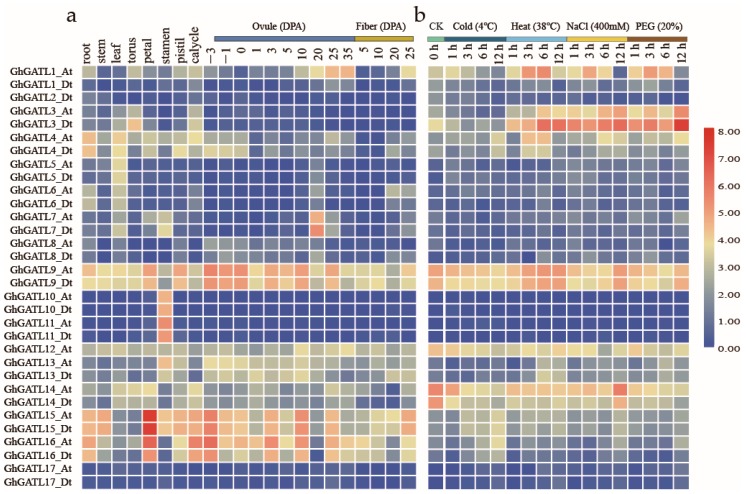
Expression profiles of *GhGATL* genes in different tissues (**a**), and under different stresses including cold, heat, NaCl, and PEG (polyethylene glycol) (**b**). Levels of gene expression are depicted in different colors on the scale, red represents high expression and blue represents low expression. DPA is an acronym for days post-anthesis.

The *GhGATL* gene expression profiles were investigated in different tissues and under various stresses, because the reference genome of the transcriptome is the *G. hirsutum* NAU (v1.1) genome, the transcription information of *GhGATL12_Dt* is lacking. The results showed that *GhGATL* genes were significantly different in each vegetative tissue and reproductive tissue, include roots, stems, leaves, torus, petal, stamen, pistil, and calycle. In particular, *GhGATL10_At/Dt* and *GhGATL11_At/Dt* were specifically expressed in stamens, suggesting that these genes may function in the growth and development of stamens (Figure 5a). Cotton fiber regulation is an important part of cotton research, there was no gene specifically expressed in stages of ovule development (−3, −1, 0, 1, 3, 5, 10, 20, 25, and 35 days) or stages of fiber development (5, 10, 20, and 25 DPA). However, *GhGATL15_At/Dt* expression was found to increase gradually along with fiber growth, suggesting that *GhGATL15_At/Dt* may contribute to the fiber elongation process. In addition, there were six pairs of *GhGATL* genes including *GhGATL9_At/Dt*, *GhGATL12_At*, *GhGATL13_At/Dt*, *GhGATL14_At/Dt*, *GhGATL15_At/Dt,* and *GhGATL16_At/Dt* that were highly expressed in almost all tissues, however, with no significant tissue specificity. Among them, the *GhGATL9_At/Dt* belonged to the GATL-c subgroup, which was the smallest group and contained at least one gene from each dicot species and *GhGATL12_At*, *GhGATL13_At/Dt*, *GhGATL14_At/Dt*, *GhGATL15_At/Dt*, and *GhGATL16_At/Dt* belonged to GATL-a and GATL-b groups which had a large positive selection value (Figure 5a). Additionally, most of the *GhGATL* gene expression patterns did not exhibit significant differences under cold, heat, PEG (polyethylene glycol), and NaCl treatments, indicating that the expression of *GhGATL* genes was less affected by stress. We only found *GhGATL3_At/Dt* displayed down-regulated patterns under cold treatments, indicating that *GhGATL3_At/Dt* might be a negative regulator under exposure to cold stress (Figure 5b).

### 3.6. Overexpression of the GhGATL Genes in Arabidopsis

**Figure 6:**
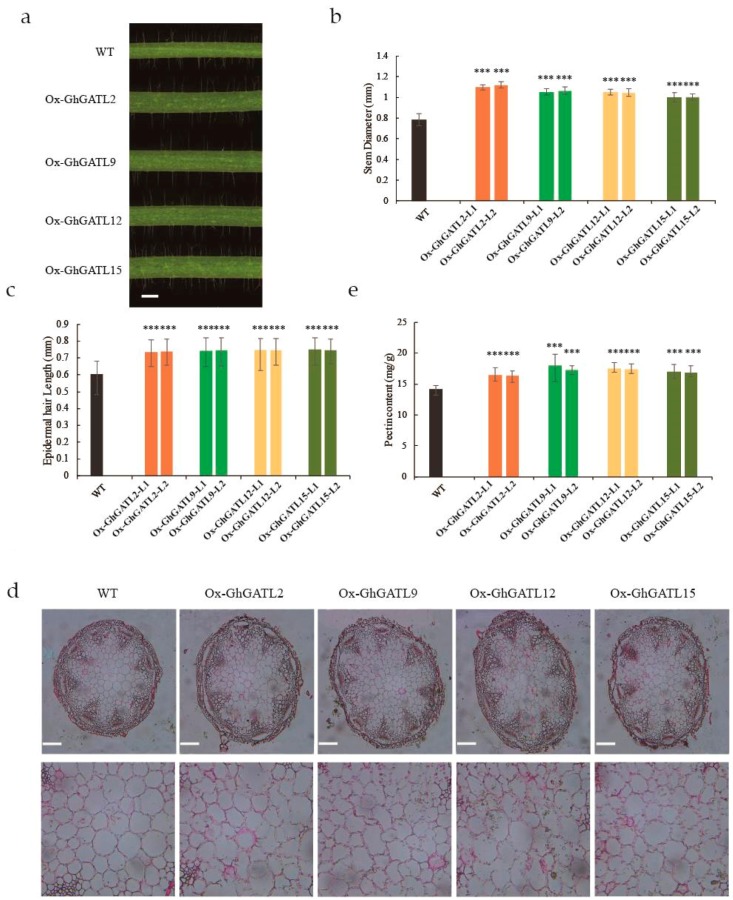
Phenotypic observation and pectin content detection between overexpressing *GhGATL* genes plants and wild type. (**a**) The middle part of the stem between the rosette leaves and the first leaf after 35 days of growth. Bar = 1 mm. (**b**) Measurement of stem diameter and (**c**) epidermal hair after 35 days of growth. (**d**) The cross-section of the stem was stained with ruthenium red after 35 days of growth. Bar = 150 μm. (**e**) Determination of pectin content in stem by carbazole reagent reaction. Significant differences compared with wild type (WT) (Student’s t test): *** *p* < 0.001.

To investigate the function of the GhGATL gene family in plants, four genes were selected including *GhGATL2*, *GhGATL9*, *GhGATL12*, and *GhGATL15* from GATL-a, GATL-b, GATL-c, and GATL-d groups to generate overexpression lines, respectively. The *GhGATL2* was unique in the D subgenome, *GhGATL9* belonged to the GATL-c subgroup which contained at least one gene from each dicot plant, *GhGATL12*, and *GhGATL15* were the most highly expressed genes in subgroup GATL-a and GATL-b which had the highest positive selection during evolution. We generated overexpression lines under the control of CAULIFLOWER MOSAIC VIRUS (CaMV) 35S promoter in *Arabidopsis* (Columbia-0). Two representative overexpression lines were selected in each of the overexpressed *GATL* gene lines in the T3 generation and detected gene expression by RT-PCR, the T4 generation was obtained by self-cross. The T4 generation was grown under normal growth conditions and 20 plants were selected from each inbred line to collect phenotypic data. We observed that all transgenic lines of these genes depicted diverse phenotypes including larger stamens, longer epidermal hair, and especially thicker and stronger stem as compared to wild type (WT) (Figure 6, S3).

The GATL gene family has been reported as closely related to the GAUT gene family and might be involved in plant pectin synthesis. We determined the pectin content in *Arabidopsis* transgenic lines by staining the stem sections of transgenic lines and wild type with ruthenium red. As in a previous report, the pectin will turn red after staining and the difference in pectin content under different treatments can be directly observed [51]. We observed that the transgenic lines had a significantly deeper staining effect than wild type on the section of the stem (Figure 6d). The open source software image J was used to convert the degree of staining into gray values to quantify the effect of staining, the staining area and total gray value significantly increased in transgenic lines (Table S6). After that, we measured the pectin content of the stem by the pectin content kit. In order to eliminate the effects of different pectin contents in different structures, we removed the stem epidermis and only took the pith to measure the pectin content, and the results were consistent with the staining results (Figure 6e). Therefore, we speculated that *GhGATL* genes had a similar function and overexpressing *GhGATL* genes could increase the pectin content in *Arabidopsis* stems, which is likely to be the reason for the apparent thickening of transgenic *Arabidopsis* stems.

### 3.7. Silencing of GhGATL15 in Cotton via VIGS

**Figure 7:**
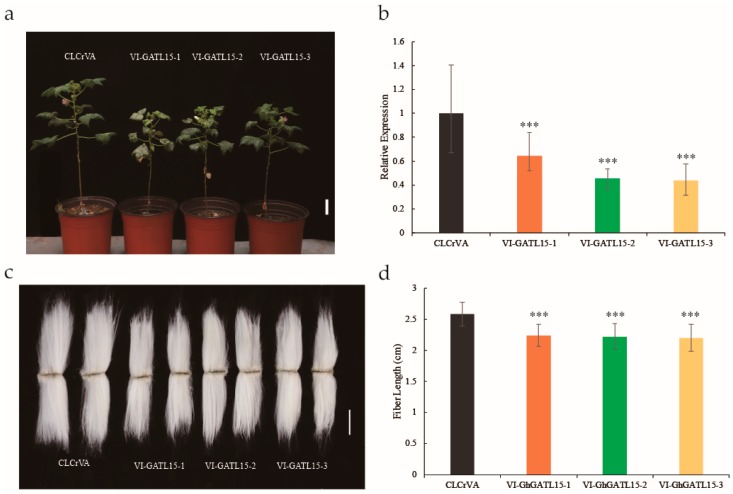
Phenotypes of *GhGATL15* virus induced gene silencing (VIGS) plants and control plants. (**a**) Morphology of the control plants and silencing *GhGATL15* gene plants (VI-GhGATL15) after three months of growth. Bar = 5 cm. (**b**) qRT-PCR analysis of *GhGATL15* in the control plants and VI-GhGATL15 plants. (**c**) Morphology of fiber between the control plants and VI-GhATL15 plants. Bar = 1 cm. (**d**) Measurement of fiber length. Significant differences compared with the control plant (Student’s t test): *** *p* < 0.001.

Fiber growth regulation is an important part of cotton research. In previous reports, the *GATL* gene was considered to be possibly involved in the fiber growth regulation of cotton. In addition, we found the expression of *GhGATL15* gradually increased with the elongation of fiber. To further identify the functional role of *GhGATL15* genes, a VIGS assay was performed in cotton and the expression of *GhGATL15* was tested by qRT-PCR to evaluate the gene silencing effects. First, two weeks after VIGS when the third leaf was grown, the stem of the third leaf from the control plants (CLCrVA) and *GhGATL15* silencing plants (VI-GhGATL15) were tested for *GhGATL15* gene expression level and pectin content. The results showed that pectin content was significantly down-regulated with the decrease of *GhGATL15* gene expression, indicating that the *GhGATL15* gene is related to the production of pectin in cotton (Figure S4). Following this, three two-month-older cotton plants were selected to collect phenotypic data. The VI-GhGATL15 showed shorter plants compared with CLCrVA (Figure 7a) and these results were consistent with the expression of *GhGATL15* tested by qRT-PCR (Figure 7b). In addition, the cotton fiber length tended to become slightly shorter (Figure 7c,d) in VI-GhGATL15 plants suggesting that the *GhGATL15_At/Dt* gene might function in cotton fiber elongation. Moreover, the seed size of VI-GhGATL15 was significantly smaller than control plants, especially the width of the seeds (Figure S5).

## 4. Discussion

Pectin is a major polysaccharide component in the plant cell wall [3] and is an important compound that promotes plant growth and fiber elongation in cotton [52]. The GATL gene family was identified as closely related to the GAUT family which has been found to be involved in pectin synthesis [11]. The GATL gene family has been studied in model plants such as *Arabidopsis* and rice [27,28], but few studies of *GATL* genes have been performed in cotton. In this study, the evolution, structure, and function of the GhGATL genes family in *G. hirsutum* were systematically analyzed, in order to better understand their roles in plant growth and development for further studies.

### 4.1. The Cotton GATL Family Expanded during Evolution

*G. hirsutum* is a natural allotetraploid plant, an ideal plant for polyploid researching in evolution. Previous studies have shown that *G. hirsutum* was formed by natural hybridization of the diploid cotton species *G. arboreum* (A2) and *G. raimondii* (D5) and subsequent chromosome doubling, along with natural and human selection [46]. We found that the number of *GATL* genes in *G. hirsutum* was equal to the total number of *GATL* genes present in *G. arboreum* (A2) and *G. raimondii* (D5). The Dt-subgenome had one more gene (*GhGATL2*) than the At-subgenome of *G. hirsutum* in accordance with *G. raimondii* having one more gene (*GrGATL2*) than *G. arboreum*. Based on the phylogenetic analysis of *GATL* genes, the genetic relationships of *GATL1*, *GATL4*, *GATL5*, and *GATL6* genes were closest with *GATL2* (Figure 1), and synteny analysis showed a collinear relationship between *GATL2*, *GATL4*, and *GATL6* (Figure 3). Therefore, we hypothesized that the *GATL2* gene might have been produced through segmental duplication and translocation between chromosomes in the *G. raimondii*. This suggested that no change in the number of *GATL* genes occurred after the formation of allotetraploids and WGD was the major impetus underlying the expansion of *GhGATL* genes in *G. hirsutum*.

During evolution, major chromosome mutations such as segmental duplication and translocation can facilitate rapid adaptation of plants to new environmental changes [53]. In this study, we found that the *GATLs* were clearly divided into five groups. However, the *GATLs* from *Physcomitrella patens* and *Sphagnum fallax* were limited into only two groups (GATL-a and GATL-e); the maize, rice, and sorghum *GATLs* were distributed into three groups (GATL-b, GATL-d, and GATL-c); and most dicot species had *GATLs* in all five groups in the phylogenetic tree. We found that the number of *GATL* gene groups had significant differences among species and whether dicots will acquire some novel function due to more *GATL* gene groups necessitates further study. Moreover, we found that GATL-c was the smallest group, but it contained at least one gene from each dicot species and did not have any *GATL* genes from monocots, suggesting it might have an indispensable function in dicots. The GATL-a, GATL-b, and GATL-d groups have undergone abundant expansion compared to GATL-c and GATL-e groups, and selective stress analysis showed strong positive selection pressure in GATL-a and GATL-b groups compared with the other three groups, indicating that the *GATL* genes from GATL-a and GATL-b groups might have more important functions in the plant during evolution.

### 4.2. GhGATL Genes Were Highly Conserved during Evolution

In our study, we found that *GhGATL* genes all have a conserved Glyco_transf_8 (PF01501) domain and most lack introns consistent with previous reports [11]. In the motif analysis, *GhGATL1_Dt* and *GhGATL8_At* lacked two and one motifs, respectively (Figure 4C), but the missing motifs only appeared in the N-terminal of the Glyco_transf_8 (PF01501) domain, the main domain was not changed. In addition, we found that the lack of motif did not affect its classification in the evolutionary tree, which also showed that the missing motif did not affect the integrity of the conserved sequence. Although there were significant differences in protein sequences between *GATLs* and *GAUTs*, the Glyco_transf_8 domain in the C-terminal domains of both *GAUTs* and *GATLs* were relatively conserved. The results of structural analysis showed that the *GATL* genes were highly conserved during cotton evolution and may have similar functions as *GAUT* genes, but there was no detailed information on the function of *GATL* genes in cotton.

### 4.3. Diverse Expression Patterns of GhGATL Genes in G. hirsutum

Based on gene expression patterns, six pairs of *GhGATL* genes were highly expressed in most tissues: *GhGATL9_At/Dt*, *GhGATL12_At*, *GhGATL13_At/Dt*, *GhGATL14_At/Dt*, *GhGATL15_At/Dt*, and *GhGATL16_At/Dt*, without significant tissue specificity (Figure 5). In these *GhGATL* genes, *GhGATL9_At/Dt* belonged to the GATL-c subgroup, which was the smallest group and contained at least one gene from each dicot species, indicating that the genes in group GATL-c have not undergone expansion but might have stable biological functions in plants. *GhGATL12_At*, *GhGATL13_At/Dt*, *GhGATL14_At/Dt*, *GhGATL15_At/Dt*, and *GhGATL16_At/Dt* belonged to GATL-a and GATL-b subgroups, which had the largest positive selection values. As in previous reports, gene duplication had significant effects on stabilizing selection via perturbation of gene expression [54,55]. For these highly expressed *GhGATL* genes, we found that the gene expression pattern was associated with the results of gene duplication analysis and selective pressure detection, suggesting that these genes may play an important role in the evolution of cotton and affect growth and development.

### 4.4. GhGATL Genes Have Similar Function and Control Fiber Growth in Cotton

In our study, we respectively selected *GhGATL2*, *GhGATL9*, *GhGATL12*, and *GhGATL15* from GATL-a, GATL-b, GATL-c, and GATL-d subgroups, and generated overexpression lines. The transgenic lines showed that *GhGATL* genes have similar functions in plant growth and the pectin content test also proved that the pectin was all significantly increased in the transgenic lines, the similar functions might be caused by the highly conserved structure of the *GhGATL* gene family. On the other hand, as the stem of transgenic *Arabidopsis* became thicker, the epidermal hair became longer than the wild type (Figure 6c). In a previous report, the trichomes of *Arabidopsis* and cotton fibers were shown to have a similar formation mechanism: the polar elongation of a single epidermal cell [56]. Then, we demonstrated that *GhGATL15* regulated cotton fibers through a virus induced gene silencing assay (Figure 7b,d). And a cotton *GT8* gene was been reported highly expressed in the 0-DPA ovules and regulated by *GbPDF1*, which was confirmed to participate in fiber initiation and early elongation [31], the cotton *GT8* gene was the *GhGATL4_Dt* we identified in this study. Therefore, we predicted that the *GhGATL* genes may be involved in the pathway of regulating plant pectin content, and may play an important role in fiber elongation in cotton (Table S7). 

## Figures and Tables

**Table 1:** Analysis of natural selection patterns using PAML.

Group	Model	LnL	Estimates of Parameters		LRT	ω for Branch
			Background (ω)	Foreground (ω)	*p*-Value	
GATL-a	Two ratio Model 2	−17,512.215008	0.07026	999	0.046449	999
	Model 0	−17,514.197630	0.07048			
GATL-b	Two ratio Model 2	−17,511.041741	0.06975	999	0.011994	999
	Model 0	−17,514.197630	0.07048			
GATL-c	Two ratio Model 2	−17,509.850784	0.06975	0.57126	0.003193	0.57126
	Model 0	−17,514.197630	0.07048			
GATL-d	Two ratio Model 2	−17,514.197593	0.07048	1.82286	0.993136	0.07048
	Model 0	−17,514.197630	0.07048			
GATL-e	Two ratio Model 2	−17,511.322904	0.06997	2.01258	0.016494	2.01258
	Model 0	−17,514.197630	0.07048			

## Supplementary Materials

The following are available online at https://www.mdpi.com/2073-4425/11/1/64/s1. Figure S1: Sequencing and comparing differential *GhGATL* genes between the two versions of *G. hirsutum* (NAU, v 1.1) and *G. hirsutum* (HAU, v 1.1) databases, Figure S2: Phylogenetic tree of *GATL* genes constructed by MEGA software version 7.0 using minimum evolution (ME) method, Figure S3: Detection of *GhGATL* genes expression and stamen phenotype in overexpressing *GhGATL* genes lines and WT, Figure S4: Determination of pectin content between control plant and VI-GhATL15 plants, Figure S5: Phenotypes of seeds between control plant and VI-GhATL15 plants, Table S1: Protein sequences used in this study, Table S2: The GhGATL gene family between *G. hirsutum* (NAU, version 1.1) and *G. hirsutum* (HAU, version 1.1) database, Table S3: The *GATL* genes in *G. raimondii* and *G. arboreum*, Table S4: The *GhGATL* genes duplication types in *G. hirsutum*, Table S5: The motifs consensus identified by MEME, Table S6: The degree of staining of *Arabidopsis* stem sections converted into gray values, Table S7: Sequences of primers.
