# Supplementary material for: Genome-Wide Study of the GATL Gene Family in Gossypium hirsutum L. Reveals that GhGATL Genes Act on Pectin Synthesis to Regulate Plant Growth and Fiber Elongation
Source: Genes (Basel). 2020 Jan 6;11(1):64. doi: 10.3390/genes11010064 (PMC7016653; doi:10.3390/genes11010064)
Supplement: Supplementary file 1 [file genes-11-00064-s001.zip › Supplementary Files/Figure S1.pdf]

Figure S1: Sequencing and comparing differential *GhGATL* genes between the two versions of *G. hirsutum* (NAU, v 1.1) and *G. hirsutum* (HAU, v 1.1) databases

```
Gh_A05G2220     ATGCCTAAATCACCATCACTCTTCGATTTTCACCTTCTCTTACTCTTCACTATCTTTTCCTTAGCCGCCGCCGATGCCATCGCCGCTGCAAGGCCAATAACCCAACAATTCAAAGAAGCCCCACA   125
sequence        .......................................................TTTCCTTAGCCGCCGCCGATGCCATCGCCGCTGCACGGCCAATAACCCAACAATTCAAAGAAGCCCCACA   70
Consensus                                                              tttccttagccgccgccgatgccatcgccgctgca ggccaataacccaacaattcaaagaagccccaca

Gh_A05G2220     ATTCTATAACTCTGAAATTTGCCCTAAGATAGTTGGCGAAGATGATGAATCTGATAATGGAACCTCCATTTTTTTATGCTCGGATCAATCTGTTCACGTGGCAATGACCCTTGATTCAGCTTACA   250
sequence        ATTCTATAACTCTGAAATTTGCCCTAAGATAGTTGGCGAAGATGATGAATCTGATAATGGAACCTCCATTTTTTTATGCTCGGATCAATCTGTTCACGTGGCAATGACCCTTGATTCAGCTTACA   195
Consensus       attctataactctgaaatttgccctaagatagttggcgaagatgatgaatctgataatggaacctccatttttttatgctcggatcaatctgttcacgtggcaatgacccttgattcagcttaca

Gh_A05G2220     TACGGGGTTCAATGGCGGCTGTTCTTTCAATACTTCAACACTCCTCTTGTCCCCAGAATATCGTCTTCCATTTCGTCGCCTCCGCCACCGCCAACGCCTCGTTTTTACGCGCCACCATCTCTTCC   375
sequence        TACGGGGTTCAATGGCGGCTGTTCTTTCAATACTTCAACACTCCTCTTGTCCCCAGAATATCGTCTTCCATTTCGTCGCCTCCGCCACCGCCAACGCCTCGTTTTTACGCGCCACCATCTCTTCC   320
Consensus       tacggggttcaatggcggctgttctttcaatacttcaacactcctcttgtccccagaatatcgtcttccatttcgtcgcctccgccaccgccaacgcctcgtttttacgcgccaccatctcttcc

Gh_A05G2220     TCCTTCCCTTACCTCAACTTCCAAATCTACTCCTTCGACGACTCTTCTGTTTCACGTCTCATTTCAACCTCAATCCGCTCCGCACTAGACTGCCCTTTGAACTACGCTCGAAGCTACTTGGCCGA   500
sequence        TCCTTCCCTTACCTCAACTTCCAAATCTACTCCTTCGACGACTCTTCTGTTTCACGTCTCATTTCAACCTCAATCCGCTCCGCACTAGACTGCCCTTTGAACTACGCTCGAAGCTACTTGGCCGA   445
Consensus       tccttcccttacctcaacttccaaatctactccttcgacgactcttctgtttcacgtctcatttcaacctcaatccgctccgcactagactgccctttgaactacgctcgaagctacttggccga

Gh_A05G2220     TCTCCTCCCTTCATGCGTCCGCCGAATTGTGTACCTCGACTCCGACCTGGTTCTCGTCGACGACATAGCTAAACTCGCCGCAACTCAACTTGGAGACAACTCGGTCTTGGCTGCTCCAGAACATT   625
sequence        TCTCCTCCCTTCATGCGTCCGCCGAATTGTGTACCTCGACTCCGACCTGGTTCTCGTCGACGACATAGCTAAACTCGCCGCAACTCAACTTGGAGACAACTCGGTCTTGGCTGCTCCAGAACATT   570
Consensus       tctcctcccttcatgcgtccgccgaattgtgtacctcgactccgacctggttctcgtcgacgacatagctaaactcgccgcaactcaacttggagacaactcggtcttggctgctccagaacatt

Gh_A05G2220     GCAATGCAAACTTCACGTCTTACTTCACTCTAACGTTTTGGTCAAGCGCTACTTTATCCTTAACATTTGCGAACCGTAAACCTTGTTATTTTAACACCGGAGTAATGGTGATCGACCTTGATAGA   750
sequence        GCAATGCAAACTTCACGTCTTACTTCACTCTAACGTTTTGGTCAAGCGCTACTTTATCCTTAACATTTGCGAACCGTAAACCTTGTTATTTTAACACCGGAGTAATGGTGATCGACCTTGATAGA   695
Consensus       gcaatgcaaacttcacgtcttacttcactctaacgttttggtcaagcgctactttatccttaacatttgcgaaccgtaaaccttgttattttaacaccggagtaatggtgatcgaccttgataga

Gh_A05G2220     TGGAGACGAGGAGATTACACAAAAAAGATTGAAGAATGGATGGAGATTCAAAAAAGAACAAGAATCTACGAGTTGGGTTCTTTGCCGCCATTTTTATTAGTTTTTGCAGGAAACATAGTTGCCGT   875
sequence        TGGAGACGAGGAGATTACACAAAAAAGATTGAAGAATGGATGGAGATTCAAAAAAGAACAAGAATCTACGAGTTAGGTTCTTTGCCGCCATTTTTATTAGTTTTTGCAGGAAACATAGTTGCCGT   820
Consensus       tggagacgaggagattacacaaaaaagattgaagaatggatggagattcaaaaaagaacaagaatctacgagtt ggttctttgccgccatttttattagtttttgcaggaaacatagttgccgt

Gh_A05G2220     TGATCATCGATGGAATCAACATGGACTTGGGGGCGATAATTTTAAAGGTTTATGCAGGGATTTGCATCCAGGTCCAGTTAGTCTTTTACATTGGAGTGGAAAAGGAAAGCCATGGGTGAGGCTTG   1000
sequence        TGATCATCGATGGAATCAACATGGACTTGGGGGCGATAATTTTAAAGGTTTATGCAGGGATTTGCATCCAGGTCCAGTTAGTCTTTTACATTGGAGTGGAAAAGGAAAGCCATGGGTGAGGCTTG   945
Consensus       tgatcatcgatggaatcaacatggacttgggggcgataattttaaaggtttatgcagggatttgcatccaggtccagttagtcttttacattggagtggaaaaggaaagccatgggtgaggcttg

Gh_A05G2220     ATGCTAACAGGCCTTGCCCTTTGGATGCTTTATGGGCACCTTATGATCTCCTGCAAACGCCGTTTGCATTGGATTCTTG   1079
sequence        ATGCTAACAGGCCTTGCCCTTTGGATGC...................................................   973
Consensus       atgctaacaggccttgccctttggatgc

Gh_A05G2796     ATGCGTTTTATTTTATATGCGGCCGCCCTTTTACTTCTTCATGTCTTTCTCACGGTATCGTTTTGTCTTGCTATTCGGACCACAGTCGGAGGAGATGGTTGGGGTTCCGGTTTCCGGAGGCACC   125
sequence        ...............................................TCTCACGGTATCGTTTTGTCTTGCTATTCGGACCACAGTCGGAGGAGATGGTTGGGGTTCCGGTTTCCGGAGGCACC   78
Consensus                                                      tctcacggtatcgttttgtcttgctattcggaccacagtcggaggagatggttggggttccggtttccggaggcacc

Gh_A05G2796     GTATTACCGTAACGGAGTGGAGTGTCCGGTTTCCTCCGGCGACCGCAATTTAGTCCAAGTGGCAATGACTTTAGACTTTGAATACTTGCGGGGGTCGATCGCCGCCATCCACTCCGTCGTCCGCC   250
sequence        GTATTACCGTAACGGAGTGGAGTGTCCGGTTTCCTCCGGCGACCGCAATTTAGTCCAAGTGGCAATGACTTTAGACTTTGAATACTTGCGGGGGTCGATCGCCGCCATCCACTCCGTCGTCCGCC   203
Consensus       gtattaccgtaacggagtggagtgtccggtttcctccggcgaccgcaatttagtccaagtggcaatgactttagactttgaatacttgcgggggtcgatcgccgccatccactccgtcgtccgcc

Gh_A05G2796     ACGCTTCGTGTCCTGAAAACATCTTCTTTCACTTCATTGCCGCCGAGTTCGACCCCGCGAGTCCAAGAGTCTTGAGCAAACTCGTTCGCTCCACATTCCCTTCACTCAACTTCAGAATCTACATC   375
sequence        ACGCTTCGTGTCCTGAAAACATCTTCTTTCACTTCATTGCCGCCGAGTTCGACCCCGCGAGTCCAAGAGTCTTGAGCAAACTCGTTCGCTCCACATTCCCTTCACTCAACTTCAGAATCTACATC   328
Consensus       acgcttcgtgtcctgaaaacatcttctttcacttcattgccgccgagttcgaccccgcgagtccaagagtcttgagcaaactcgttcgctccacattcccttcactcaacttcagaatctacatc

Gh_A05G2796     TTCCGCGAAGACACAGTGATCAACTTGATCTCTTCTTCAATCCGGCAAGCCCTCGAGAACCCACTGAACTACGCTCGGAACTACCTCGGGGACATGTTGGATCTCCAAGTAGACCGAGTCATTTA   500
sequence        TTCCGCGAAGACACAGTGATCAACTTGATCTCTTCTTCAATCCGGCAAGCCCTCGAGAACCCACTGAACTACGCTCGGAACTACCTCGGGGACATGTTGGATCTCCAAGTAGACCGAGTCATTTA   453
Consensus       ttccgcgaagacacagtgatcaacttgatctcttcttcaatccggcaagccctcgagaacccactgaactacgctcggaactacctcggggacatgttggatctccaagtagaccgagtcattta

Gh_A05G2796     TCTCGACTCAGACCTGGTCCTCGTCGATGACATCCTCAAACTCTGGAACACAACGTTGACCAACTCTCGAGTCATCGGCGCACCCGAATACTGCCATGCAAACTTCACCAAGTATTTCACGGCCG   625
sequence        TCTCGACTCAGACCTGGTCCTCGTCGATGACATCCTCAAACTCTGGAACACAACGTTGACCAACTCTCGAGTCATCGGCGCACCCGAATACTGCCATGCAAACTTCACCAAGTATTTCACGGCCG   578
Consensus       tctcgactcagacctggtcctcgtcgatgacatcctcaaactctggaacacaacgttgaccaactctcgagtcatcggcgcacccgaatactgccatgcaaacttcaccaagtatttcacggccg

Gh_A05G2796     GGTTTTGGTCGGACCCGGTAATATCCCGGGTTTTCCGGTCCAGAAAGCCGTGTTATTTCAACACGGGAGTAATGGTAATGGATTTAGTGCGTTGGAGAGAAGGGAATTACAGGAAAAGAATAGAG   750
sequence        GGTTTTGGTCGGACCCGGTAATATCCCGGGTTTTCCGGTCCAGAAAGCCGTGTTATTTCAACACGGGAGTAATGGTAATGGATTTAGTGCGTTGGAGAGAAGGGAATTACAGGAAAAGAATAGAG   703
Consensus       ggttttggtcggacccggtaatatcccgggttttccggtccagaaagccgtgttatttcaacacgggagtaatggtaatggatttagtgcgttggagagaagggaattacaggaaaagaatagag

Gh_A05G2796     AATTGGATGGAAATACAAAGGAAAAAAAAGATATACGAGTTGGGCTCATTGCCACCCTATTTGCTGGTATTTGCTGGGAATGTAGAAGGCATTGATCATAGATGGAACCAACATGGGCTTGGAGG   875
sequence        AATTGGATGGAAATACAAAGGAAAAAAAAGATATACGAGTTGGGCTCATTGCCACCCTATTTGCTGGTATTTGCTGGGAATGTAGAAGGCATTGATCATAGATGGAACCAACATGGGCTTGGAGG   828
Consensus       aattggatggaaatacaaaggaaaaaaaagatatacgagttgggctcattgccaccctatttgctggtatttgctgggaatgtagaaggcattgatcatagatggaaccaacatgggcttggagg

Gh_A05G2796     GGACAATATACGAGGTTCATGTCGGTCGTTGCATCCGGGTCCGGTCAGCTTGTTGCATTGGAGTGGGAAAGGGAAGCCATGGGTTCGACTGGATGGTGGAAACCCGTGCCCTCTTGATCATATTT   1000
sequence        GGACAATATACGAGGTTCATGTCGGTCGTTGCATCCGGGTCCGGTCAGCTTGTTGCATTGGAGTGGGAAAGGGAAGCCATGGGTTCGACTGGATGGTGGAAACCCGTGCCCTCTTGATCATATTT   953
Consensus       ggacaatatacgaggttcatgtcggtcgttgcatccgggtccggtcagcttgttgcattggagtgggaaagggaagccatgggttcgactggatggtggaaacccgtgccctcttgatcatattt

Gh_A05G2796     GGGAATCCTATGATCTTTACAAAGGGAATTTAATCAAACACCAATCTTTTCCTTCTGCTTTTGCCAATTTCTTTGAATACCCCAGCTATTTGTTTTG   1097
sequence        GGGAATCCTATGATCTTTACAAAGGGAATTTAATCAAACACCAATCTTTT................................................   1003
Consensus       gggaatcctatgatctttacaaagggaatttaatcaaacaccaatctttt

Gohir.D07G131000.1   ATGTTTCCTTCAAAGTCATTTCCACCAGCTGAAGCCATTAGGTCCTCTCATCTCCACGCCTACCTCCGGTTACCCTCATATCAGGTGTCCTCGTCAACGTACTCACCGCTTGACCGGTTCTCTTT   125
sequence             ..............................................CTCATCTCCACGCCTACCTCCGGTTACCCTCATATCAGGTGTCCTCGTCAACGTACTCACCGCTTGACCGGTTCTCTTT   79
Consensus                                                          ctcatctccacgcctacctccggttaccctcatatcaggtgtcctcgtcaacgtactcaccgcttgaccggttctcttt

Gohir.D07G131000.1   TCGAAAAGCCTCGGAATTTGGCAATGCCGACAAATGTCGCTCCACCGACCATAAAATCACCGGCGTTTGCGATCCTTCTTTGGTCCACGTGGCCATCACTCTTGACGTTGAGTACCTCCGTGGCT   250
sequence             TCGAAAAGCCTCGGAATTTGGCAATGCCGACAAATGTCGCTCCACCGACCATAAAATCACCGGCGTTTGCGATCCTTCTTTGGTCCACGTGGCCATCACTCTTGACGTTGAGTACCTCCGTGGCT   204
Consensus            tcgaaaagcctcggaatttggcaatgccgacaaatgtcgctccaccgaccataaaatcaccggcgtttgcgatccttctttggtccacgtggccatcactcttgacgttgagtacctccgtggct

Gohir.D07G131000.1   CAATCGCCGCCGTTCACTCCATTCTCCAGCATTCTTTGTGTCCGGAGAACGTTTTCTTCCATTTCCTCGTCTCTGATACTGATTTAGAAACCCTGGTTCGGTCCACGTTCCCTCAATTGAAGTTC   375
sequence             CAATCGCCGCCGTTCACTCCATTCTCCAGCATTCTTTGTGTCCGGAGAACGTTTTCTTCCATTTCCTCGTCTCTGATACTGATTTAGAAACCCTGGTTCGGTCCACGTTCCCTCAATTGAAGTTC   329
Consensus            caatcgccgccgttcactccattctccagcattctttgtgtccggagaacgttttcttccatttcctcgtctctgatactgatttagaaaccctggttcggtccacgttccctcaattgaagttc

Gohir.D07G131000.1   AAGGCTTACTATTTCGACCCCGCGATTGTACGGAACTTGATATCGTCTTCCGTTAGGCAAGCGCTTGAACAGCCGTTAAATTATGCTAGAAATTACTTAGCGGATCTGCTAGAGCCGTGCGTGCG   500
sequence             AAGGCTTACTATTTCGACCCCGCGATTGTACGGAACTTGATATCGTCTTCCGTTAGGCAAGCGCTTGAACAGCCGTTAAATTATGCTAGAAATTACTTAGCGGATCTGCTAGAGCCGTGCGTGCG   454
Consensus            aaggcttactatttcgaccccgcgattgtacggaacttgatatcgtcttccgttaggcaagcgcttgaacagccgttaaattatgctagaaattacttagcggatctgctagagccgtgcgtgcg

Gohir.D07G131000.1   GAGGGTGATTTATTTGGACTCTGATCTAGTTGTTGTTGACGATATCGCTAAACTATGGAGTACCAACCTAGGTTCACGGACAATCGGCGCACCGGAATATTGCCACGCCAACTTCACAAAATATT   625
sequence             GAGGGTGATTTATTTGGACTCTGATCTAGTTGTTGTTGACGATATCGCTAAACTATGGAGTACCAACCTAGGTTCACGGACAATCGGCGCACCGGAATATTGCCACGCCAACTTCACAAAATATT   579
Consensus            gagggtgatttatttggactctgatctagttgttgttgacgatatcgctaaactatggagtaccaacctaggttcacggacaatcggcgcaccggaatattgccacgccaacttcacaaaatatt

Gohir.D07G131000.1   TCACGAGCAGTTTCTGGTCGGCCGAACGGTTTTCAGGGACATTCAAAGGGAGGAAGCCGTGCTATTTCAATACGGGGGTGATGGTGATAGATCTGGTGAAATGGAGGAGGGTGGGATACACGAAG   750
sequence             TCACGAGCAGTTTCTGGTCGGCCGAACGGTTTTCAGGGACATTCAAAGGGAGGAAGCCGTGCTATTTCAATACGGGGGTGATGGTGATAGATCTGGTGAAATGGAGGAGGGTGGGATACACGAAG   704
Consensus            tcacgagcagtttctggtcggccgaacggttttcagggacattcaaagggaggaagccgtgctatttcaatacgggggtgatggtgatagatctggtgaaatggaggagggtgggatacacgaag

Gohir.D07G131000.1   CGGATCGAAAGGTGGATGGAAATCCAAAAAACCGATCGGATCTACGAGCTGGGTTCGTTGCCGCCGTTCCTGTTGGTTTTTGCGGGACGCGTGGCACCCATCGAGCATAGATGGAACCAGCATGG   875
sequence             CGGATCGAAAGGTGGATGGAAATCCAAAAAACCGATCGGATCTACGAGCTGGGTTCGTTGCCGCCGTTCCTGTTGGTTTTTGCGGGACGCGTGGCACCCATCGAGCATAGATGGAACCAGCATGG   829
Consensus            cggatcgaaaggtggatggaaatccaaaaaaccgatcggatctacgagctgggttcgttgccgccgttcctgttggtttttgcgggacgcgtggcacccatcgagcatagatggaaccagcatgg

Gohir.D07G131000.1   GTTGGGTGGGGACAACGTGAGGGGAAGTTGCCGAGGCTTGCATCCGGGTCCCGTCAGCCTCTTGCATTGGTCCGGTAGTGGCAAGCCATGGGATAGGCTTGATTCCAGACGGCCATGCCCACTCG   1000
sequence             GTTGGGTGGGGACAACGTGAGGGGAAGTTGCCGAGGCTTGCATCCGGGTCCCGTCAGCCTCTTGCATTGGTCCGGTAGTGGCAAGCCATGGGATAGGCTTGATTCCAGACGGCCATGCCCACT..   952
Consensus            gttgggtggggacaacgtgaggggaagttgccgaggcttgcatccgggtcccgtcagcctcttgcattggtccggtagtggcaagccatgggataggcttgattccagacggccatgcccact

Gohir.D07G131000.1   ACGCATTATGGGCACCTTACGACTTGTACCGACACTCACATTG   1043
sequence             ...........................................   952
Consensus
```
