# Supplementary material for: Genome-Wide Study of the GATL Gene Family in Gossypium hirsutum L. Reveals that GhGATL Genes Act on Pectin Synthesis to Regulate Plant Growth and Fiber Elongation
Source: Genes (Basel). 2020 Jan 6;11(1):64. doi: 10.3390/genes11010064 (PMC7016653; doi:10.3390/genes11010064)

Figure S2: Phylogenetic tree of ***GATL*** genes constructed by MEGA software version 7.0 using minimum evolution (ME) method. The prefixes Gh, Ga, Gr, At, Os, Sobic, Zm, Thecc, VIT, Prupe, Potri, MD, Glyma, Pp3c and Sphfalx represent ***G. hirsutum, G. arboreum, G. raimondii, Arabidopsis thaliana, Oryza sativa, Sorghum bicolor, Zea mays, Theobroma cacao, Vitis vinifera Genoscope, Prunus persica, Populus trichocarpa, Malus domestica, Glycine max, Physcornitrella patens*** and ***Sphagnum fallax***, respectively. "At" and "Dt" indicate the A- and D- subgenomes in *G. hirsutum* respectively. Bootstrapping was used to provide reliability for nodes.

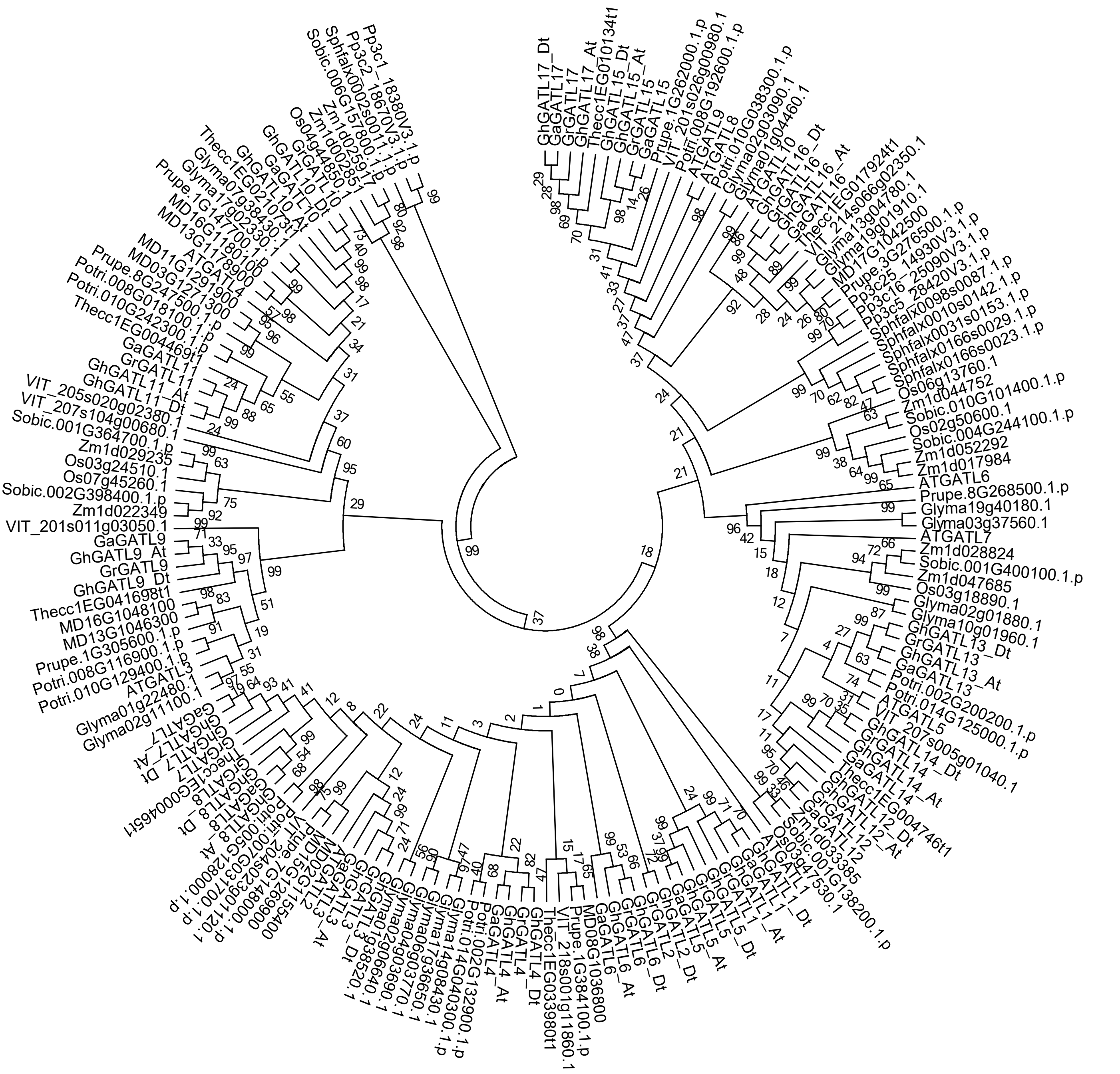

Supplement: Supplementary file 1 [file genes-11-00064-s001.zip › Supplementary Files/Figure S2.pdf]
