# Supplementary material for: Genome-Wide Study of the GATL Gene Family in Gossypium hirsutum L. Reveals that GhGATL Genes Act on Pectin Synthesis to Regulate Plant Growth and Fiber Elongation
Source: Genes (Basel). 2020 Jan 6;11(1):64. doi: 10.3390/genes11010064 (PMC7016653; doi:10.3390/genes11010064)

Figure S3: Detection of *GhGATL* genes expression and stamen phenotype in overexpressing *GhGATL* genes lines and WT. (a) qRT-PCR analysis of *GhGATL* genes in WT and overexpressing *GhGATL* genes lines. (b) Morphology of stamen between WT and overexpressing *GhGATL* genes lines. Bar = 0.5 mm. (c) Measurement of stamen length. Significant differences compared with WT (Student's *t*-test): *, P < 0.05, **, P < 0.01, ***, P < 0.001.

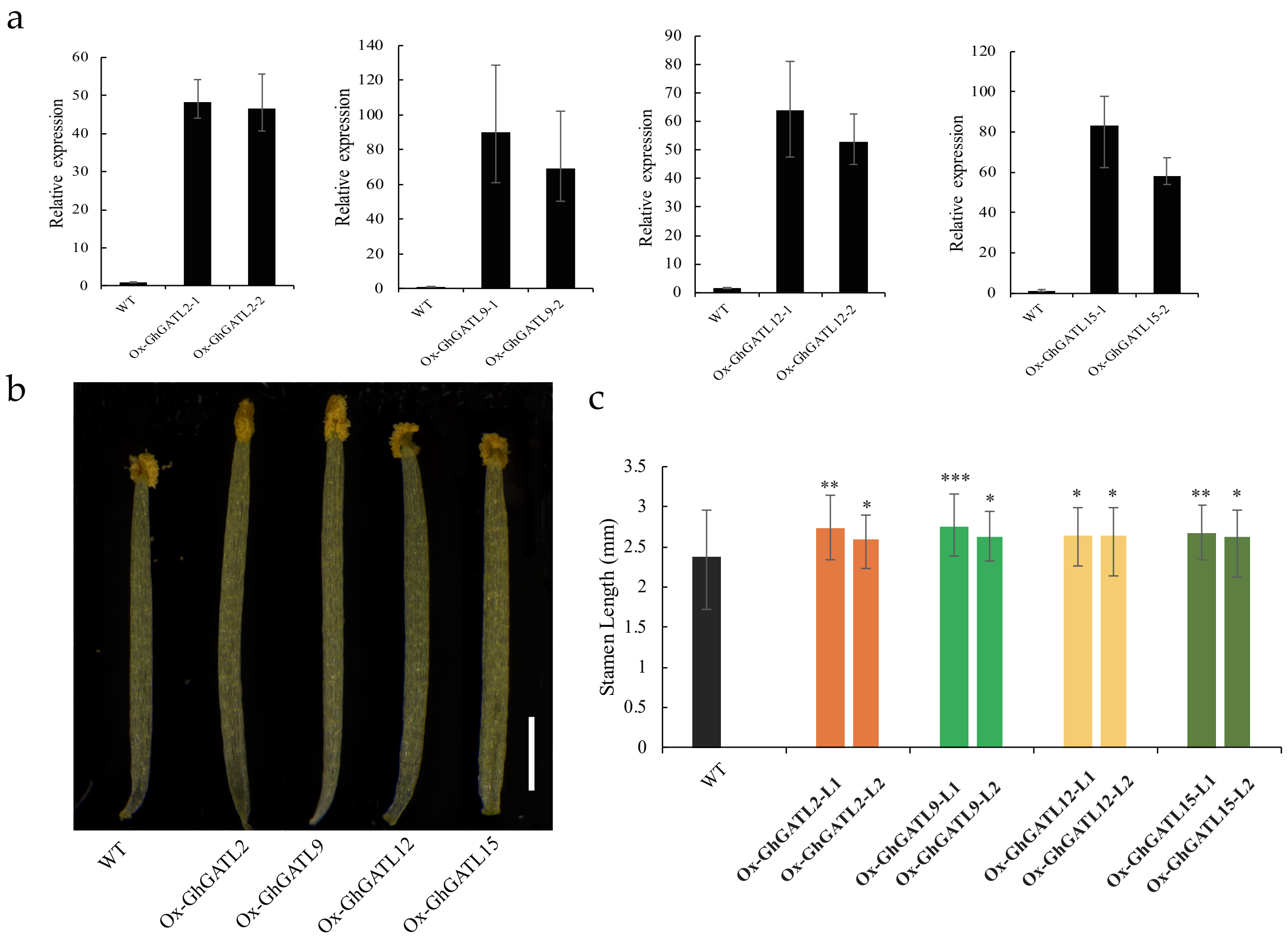

Supplement: Supplementary file 1 [file genes-11-00064-s001.zip › Supplementary Files/Figure S3.pdf]
