# Supplementary material for: Genome-Wide Study of the GATL Gene Family in Gossypium hirsutum L. Reveals that GhGATL Genes Act on Pectin Synthesis to Regulate Plant Growth and Fiber Elongation
Source: Genes (Basel). 2020 Jan 6;11(1):64. doi: 10.3390/genes11010064 (PMC7016653; doi:10.3390/genes11010064)

**Figure S4:** Determination of pectin content between control plant and VI-GhATL15 plants. (a) qRT-PCR analysis of *GhGATL15* expression in control plant and VI-GhGATL15 plants. (b) Determination of pectin content in third leaf stem by carbazole reagent reaction. Significant differences compared with WT (Student's *t*-test): ***, P < 0.001.

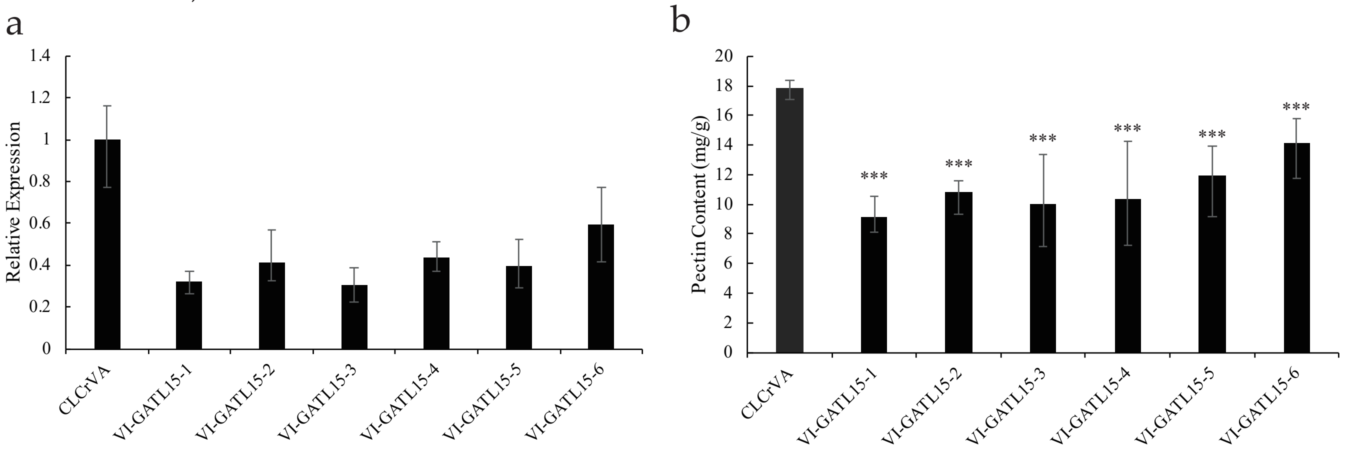

Supplement: Supplementary file 1 [file genes-11-00064-s001.zip › Supplementary Files/Figure S4.pdf]
