# Supplementary material for: Genome-Wide Study of the GATL Gene Family in Gossypium hirsutum L. Reveals that GhGATL Genes Act on Pectin Synthesis to Regulate Plant Growth and Fiber Elongation
Source: Genes (Basel). 2020 Jan 6;11(1):64. doi: 10.3390/genes11010064 (PMC7016653; doi:10.3390/genes11010064)

Figure S5: Phenotypes of seeds between control plant and VI-GhATL15 plants. (a-b) Morphology of seeds between control plant and VI-GhATL15 plants. Bar = 1cm. (c) Measurement of seed length. (d) Measurement of seed width. Significant differences compared with control plant (Student's *t*-test): ***, $P < 0.001$.

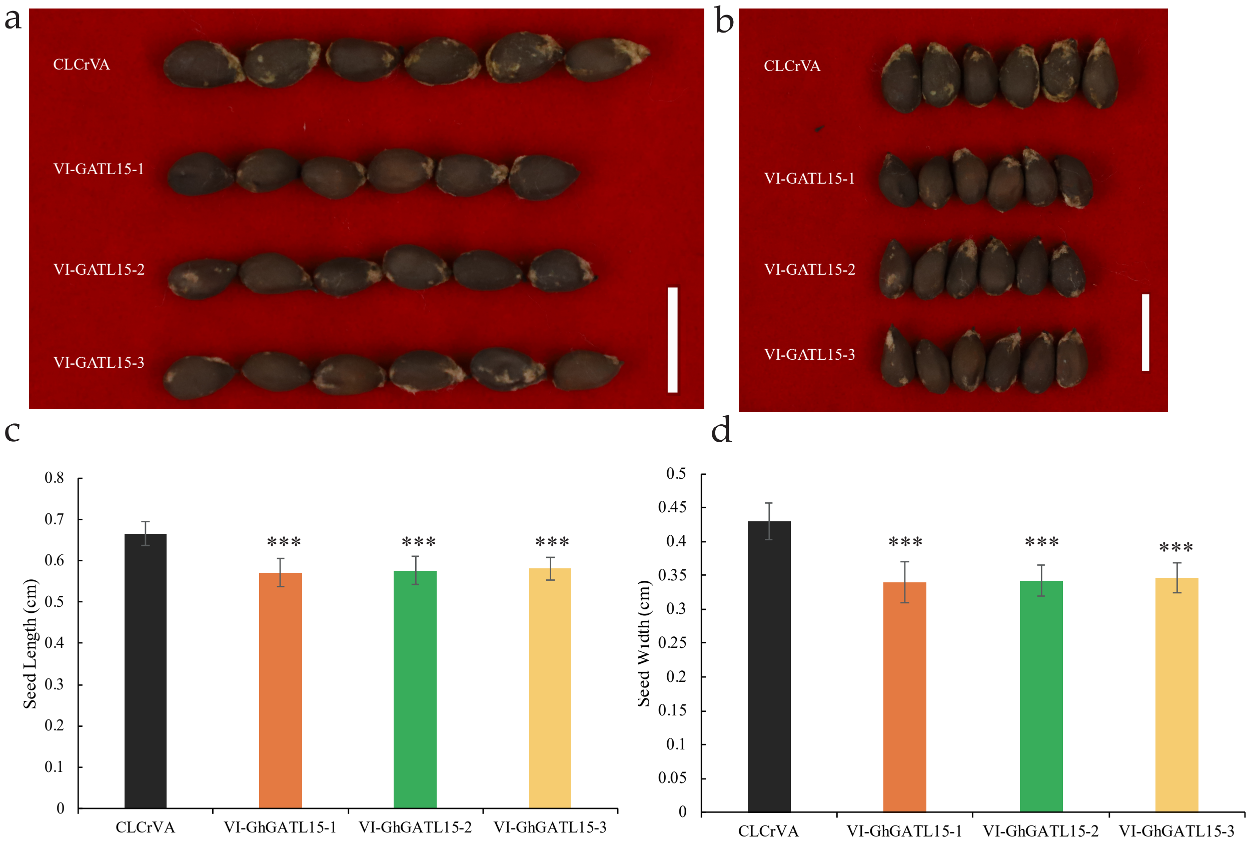

Supplement: Supplementary file 1 [file genes-11-00064-s001.zip › Supplementary Files/Figure S5.pdf]
