# Supplementary material for: Genome-Wide Study of the GATL Gene Family in Gossypium hirsutum L. Reveals that GhGATL Genes Act on Pectin Synthesis to Regulate Plant Growth and Fiber Elongation
Source: Genes (Basel). 2020 Jan 6;11(1):64. doi: 10.3390/genes11010064 (PMC7016653; doi:10.3390/genes11010064)
Supplement: Supplementary file 1 [file genes-11-00064-s001.zip › Supplementary Files/Table S1.docx]

**Supplementary Table 1. Protein sequences used in this study.**

>GhGATL1_At

MRRNHLSYPNSLFFTFNINRLFRYRVNIVCIYIDSVLMMKVICTMAHLLHRLVIFTILITMAATVTPITHQHSKEASKFYNSPACLSISEPYILCSHQALHLAMTIDKAYIRGSMAAILSVLQHSSCPHNIAFHFLISATANASLLRATISSSFPYLYFRLYPFDHSSVPLISTSIRSALDCPLNYARIYLASLLPSCIHRVLYLDSDLILVDDVAELAAIQLGDNSVLAATEYCSANFVSYFTPTFWSNPCLSLTFANRKACYFNTGVMVMDLDRWREGDYTRKIEEWMEIQKRMRIYELGSLPPFLLVFAGNIVPVDHRWNQHGLGGDNFDGLCRDLHPGPVSVLHWSGKGKPWARLDAKTPCLLDALWASYDLMDTSFAFDS

>GhGATL12_At

MLWVMQFSGFFSAAMLMIVLSPSFQSFPPAEAIRSSHLHAYLRLSSYQVSSSPYSPLDRFSFRKASEFGNADKCRSTDHKITGVCDPSLVHVAITLDVEYLRGSIAAVHSILQHSLCPENVFFHFLVSDTDLETLVRSTFPQLKFKAYYFDPAIVRNLISSSVRQALEQPLNYARNYLADLLEPCVRRVIYLDSDLVVVDDIAKLWSTNLGSRTIGAPEYCHANFTKYFTSGFWSAERFSGTFKGRKPCYFNTGVMVIDLVKWRRVGYTKRIERWMEIQKTDRIYELGSLPPFLLVFAGRVAPIEHRWNQHGLGGDNVRGSCRGLHPGPVSLLHWSGSGKPWDRLDSRRPCPLDALWAPYDLYRHSH

>GhGATL10_Dt

MALWRTLPIPIASPHGLILSFLILLHSAVTTTVAIRVRTIIHDPSPVKLPVFREAPAFRNGDSCGSNKDDRIHIAMTLDENYLRGTMAAVLSMLQHSTCPENLSFHFLCAHSDVELVSSIESTFPYLNFKIYRFDSNRVRGKISKSIRQALDQPLNYARIYLADILPADVKRVLYLDSDLVVVDDIGKLWDVDMEDKVLAAPEYCHANFTVYFNDAFWSDPVLSNTFQGRNPCYFNTGVMVVDVDKWRKGGYTKKVEQWMAFHKKKRIYHLGSLPPFLLVLAGNIKPVDHRWNQHGLGGDNFEGKCRNLHPGPISLLHWSGKGKPWLRLDSRKPCVVDHLWAPYDLYRSSRHFLEE

>GhGATL4_Dt

MSKPPRFDLLIVLLFTVIALASNSVTATPITQQFKEAPQFYNSPDCAVLVDEDESDGEASILCSDQAVHVAMTLDTAYIRGSMAAILSVLQHSSCPQNIAFHFVASATANATFLLATISSSFPYLNFRVYPFDDSSVSRLISTSIRSALDCPLNYARSYLANLLPPCVRRVVYLDSDLVLVDDIAKLAATPLGDDPVLAAPEHCNANFTTYFTPTFWSNPSLSLTFANRKPCYFNTGVMVIDLDRWREGDYTTKIEEWMELQKRIRIYELGSLPPFLLVFAGNIVPVDHRWNQHGLGGDNFRGLCRDLHPGPVSLLHWSGKGKPWARLDANRPCPLDALWAPYDLLETPFALDS

>GhGATL6_Dt

MPKPQLLHFLHVLSFNVFAIAAVIATVTAATVTLQLKEAPQFYNSPDCPSITNIEDDPNGESSIFCSDQAVHVAVTLDSAYIRGSMAVTLSVLQHSSCPQNIVFHFVTSAAANVSFLHATISASFPYLNFQIYPFDDASVSRLISTSVRSALDCPLNYARSYLANLLPLCVTRVVYLDSDLILVDDIAKLAATPLGDNSVLAAPQYCNANFTSYFTTTFWSNPSLYLTFANRKPCYFNTGVMVMDLDRWRNGDYTTKIEEWMEIQKQMRIYELGSLPPFLLVFAGNIVPVDHRWNQHGLGGDNFRGLCRNLHPGPVSLLHWSGKGKPWARLDANRPCPLDALWAPYDLLQTPFVLDS

>GhGATL8_Dt

MPKLALIPPHDHHRHHRQLLLIFLLISATTAAPASATTNYQVFKEAPQFYNSPTCPSIHTNGMCCDQAVHVAMTLDTAYLRGLMAAILSILQHSSCPQNILFHFIASATANHQHFRYTISRAFPALVFQIYPYDSKALSGLISTSIRSALDCPLNYARNYLPNLLPRCLRRVVYLDSDLVLVDDMAKLAATPLGDSSVLAAPEYCNANFTSYFTPTFWSNPTLSLTFSGRKACYFNTGVMVIDLQRWRAGDYTTEIVEWMELQKRMRIYELGSLPPFLLVFAGNIAPVDHRWNQHGLGGDNYRGLCRDLHPGPTSLLHWSGKGKPWARLDANRPCPLDAFWAPYDLLQTSSALES

>GhGATL10_At

MALWRTLPIPIASPHGLILSFLILLHSAVTTTVAIRVRTIIHDPSPVKLPVFREAPAFRNGDSCGSNKDDRIHIAMTLDENYLRGTMAAVLSMLQHSTCPENLSFHFLCAHSDVELVSSIESTFPYLNFKIYRFDSNRVRGKISKSIRQALDQPLNYARIYLADILPADVKRVLYLDSDLVVVDDIGKLWDVDMEDKVLAAPEYCHANFTVYFNDAFWSDPVLSNTFQGRNPCYFNTGVMVVDVDKWRKGGYTKKVEQWMAFQKKKRIYHLGSLPPFLLVLAGNIKPVDHRWNQHGLGGDNFEGKCRNLHPGPISLLHWSGKGKPWLRLDSRKPCVVDHLWAPYDLYRSSRHFLEE

>GhGATL8_At

MAAILSILPHSSCPQNILFHFIASDTANHHHFRYTISRAFTALVFQIYPYDSKAVSGLISSSIRSALDCPLNYARNYLPNLLPHCLRRVVYLDSDLVLVDDMAKLTATPLGDSSVLAAPEYCNANFTSYFTPTFWSNPTLSLTFSGRNACYFNTGVMVIDLQRWRAGDYTTKIVEWMELQKRMRIYELGSLPPFLLVFAGNIAPVDHRWNQHGLGGDNYRGLCRDLHPGPTSLLHWSGKGKPWARIDANRPCPLDAFWAPYDLLQTSSSLES

>GhGATL6_At

MPKSQLLHFLHVLAFNVFAIAAVIATVTAATVTLQLKEAPQFYNSPDCPSITNIEDDPNGESSIFCSDQAVHVAVTLDSAYIRGSMAVTLSVLQHSSCPQNIVFHFVTSAAANVSFLHATISSSFPYLNFQIYPFDDASVSRLISTSIRSALDCPLNYARSYLANLLPLCVTRVVYLDSDLILVDDIGKLAATPLGDNSVLAAPQYCNANFTSYFTTTFWSNPSLSLTFANRKPCYFNTGVMVMDLDRWRNGDYTTKIEEWMEIQKQMRIYELGSLPPFLLVFAGNIVPVDHRWNQHGLGGDNFRGLCRNLHPGPVSLLHWSGKGKPWARLDANRPCPLDALWAPYDLLQTPFVLDS

>GhGATL17_At

MRFILYAAALLLLHVFLTVSFCLAIRTTVGGDGLGFRFPEAPYYRNGVECPVSSGDRNLVQVAMTLDFEYLRGSIAAIHSVVRHASCPENIFFHFIAAEFDPASPRVLSKLVRSTFPSLNFRIYIFREDTVINLISSSIRQALENPLNYARNYLGDMLDLQVDRVIYLDSDLVLVDDILKLWNTTLTNSRVIGAPEYCHANFTKYFTAGFWSDPVISRVFRSRKPCYFNTGVMVMDLVRWREGNYRKRIENWMEIQRKKKIYELGSLPPYLLVFAGNVEGIDHRWNQHGLGGDNIRGSCRSLHPGPVSLLHWSGKGKPWVRLDGGNPCPLDHIWESYDLYKGNLIKHQSFPSAFANFFEYPSYLF

>GhGATL1_Dt

MVGDPWPLFFPYFNTPLVPTTLPSISSSPPPQTPHFYAPPYPPPSLTSTSDYPFDHSSVAPLISTSIRSALDCPLNYARIYLASLLPSCIHRVLYLDSDLILVDDIAELAAIQLGDNSVLAATEYCSANFVSYFTPTFWSNPCLSLTFANRKACYFNTGVMVMDLDRWREGDYTRKIEEWMEIQKRMRIYELGSLPPFLLVFAGNIVPVDHRWNQHGLGGDNFDGLCRDLHPGPVSVLHWSGKGKPWARLDAKTPCRLDALWASYDLLDTSFAFDS

>GhGATL17_Dt

MRFILYAAALLLLHFLLTASFCLAIRTTVGGDGLGFRFPEAPYYRNGVECPVSSGDRNLVQVAMTLDFEYLRGSIAAVHSVVRHASCPENIFFHFIAAEFDPASPRVLSKLVRSTFPSLNFRIYIFREDTVINLISSSIRQALENPLNYARNYLGDMLDLQVGRVIYLDSDLVLVDDILKLWNTTLTNSRVIGAPEYCHANFTKYFTAGFWSDPVISRVFRSRKPCYFNTGVMLMDLVRWREGNYRKRIENWMEIQRKKKIYELGSLPPYLLVFAGNVEGIDHRWNQHGLGGDNIRGSCRSLHPGPVSLLHWSGKGKPWVRLDGGSPCPLDHIWESYDLYKGNLIKHQSFPSAFANFFEYPSYLF

>GhGATL11_Dt

MVALPFLGRLSFLLLIATTTTTAFAIGIHHGSIIRKPSSGVSNFREAPKFRNGDVCGTNASERIHIAMTLDVNYLRGTMAAVLSVLMHSTCPENTEFHFLWGKYEPEVLVSINSTFPYLNFRLYHFDSDRVRGKISRSIRQALDQPLNYARIYLADMLPPDVKRVLYLDSDLVVVDDIVKLWEVDLEGKVLAAPEYCHANFTKYFTDLFWSDKELSSTFNGRKPCYFNTGVMVVDVDKWRQGGYTPKVEEWMALQKQKRIYTLGSLPPFLLVLAGNIKAVHHRWNQHGLGGDNLEGKCRSLHPGPISLLHWSGKGKPWLRLDSRKPCAVDHLWAPYDLYGSPDHSLEE

>GhGATL7_Dt

MPEPHGHLHRHHLLLLLFLLITATNSAPASATTTNYQRFKEAPQFYNSPACPSLDTNEMCSHQAVHVAMTLDAAYLRGSMAAILSVLQHSSCPQNIRFHFIPSSTANHQHLRLTISRSFPSLKFQIYPYDSSKVSGLISTSIRSALDCPLNYARNYLANLLPPCLSRVVYLDSDLVLVDDIAKLAATPLGDRSVLAAPEYCNANFTSYFTPTFWSNPTLSLTFAGRKACYFNTGVMVIDLQRWRQGDYTTKIIEWMELQKRMRIYELGSLPPFLLVFAGNIAPVDHRWNQHGLGGDNYRGLCRNLHPGPVSLLHWSGKGKPWVRLDANRPCPLDALWAPYDLLQTPFALES

>GhGATL5_Dt

MPKSPSLFDFHLLLLFTIFSLAAADAIAAARPITQQFKEAPQFYNSEICPKIVGEDDESDNGTSIFLCSDQSVHVAMTLDSAYIRGSMAAVLSILQHSSCPQNIVFHFVASATANASFLHATISSSFPYLSFQVYSFDDSSVSRLISASIRSALDCPLNYARSYLADLLPSCVRRIVYLDSDLVLVDDIAKLAATQLGDNSVLAAPEHCNANFTSYFTLTFWSSPTLSLTFANRKPCYFNTGVMVIDLDRWRRGDYTKKIEEWMEIQKRTRIYELGSLPPFLLVFAGNIVAVDHRWNQHGLGGDNFKGLCRDLHPGPVSLLHWSGKGKPWVRLDANRPCPLDALWAPYDLLQTPFALDS

>GhGATL7_At

MPKPHDHLHRHHLLLLLFLLITATNSAPASATTTNYQRFKEAPQFYNSPACPSLDTNEMCSHQAVHVAMTLDAAYLRGSMAAILSALQHSSCPQNIRFHFIPSATANHHHLRLTISRSFPSLKFQIYPYDSSKVSGLISTSIRSALDCPLNYARNYLANLLPPCLSRVVYLDSDLVLVDDIAKLAATPLGDRSVLAAPEYCNANFTSYFTPTFWSNPTLSLTFAGRKACYFNTGVMVIDLQRWRQGDYTTKIIEWMELQKRIRIYELGSLPPFLLVFAGNIAPVDHRWNQHGLGGDNYRGLCRNLHPGPVSLLHWSGKGKPWVRLDANRPCPLDALWAPYDLLQTPFALES

>GhGATL14_At

MTDLKRILLFFRSSLFILLADWLDFKSKKKKRRGICSSKILWIRRFSGFFFAAMLMIILSPSLHSFPPAEAIKSSHLDSYLRLPSYQASHSSKDRFSFRKASGFRNADECGFTDRKITGVCDPSLVHVAITLDVVYLRGSIAAVHSILQHSLCPENIFFHFIVSDADLETLVRSTIPQLKFKVYYFDPEIVRNLISSSVRQALEQPLNYARNYLADLLEPCVRRVIYLDSDLVVVDDITKLWITNLGSRTIGAPEYCHANFTNYFTGGFWSDQRFSRTFKGRKPCYFNTGVMVIDLVKWRRVGYTKRIERWMQIQKRYRIYELGSLPPFLLVFAGRVAPIEHRWNQHGLGGDNVKGSCRELHPGPVSLLHWSGSGKPWLRLDSNRPCPLDALWAPYDLYGHPH

>GhGATL4_At

MSKPPRFDLLLVLLFTVIALASNSVTVATPITQQFKEAPQFYNSPDCAVLVDEDESDGEASILCSDQAVHVAMTLDTAYIRGSMAAILSVLQHSSCPQNIAFHFIASATANASFLLATISSSFPYLNFRVYPFDDSSVSRLISTSIRSALDCPLNYARSYLANLLPPCVRRVVYLDSDLVLVDDIAKLAATPLGDDSVLAAPEHCNANFTTYFTPTFWSNPSLSLTFANRKPCYFNTGVMVIDLDRWREGDYTTKIEEWMELQKRIRIYELGSLPPFLLVFAGNIVPVDHRWNQHGLGGDNFRGLCRDLHPGPVSLLHWSGKGKPWARLDANRPCPLDALWAPYDLLQTPFALDS

>GhGATL14_Dt

MVDLKRILLFFRSSLFILLADWLDFKSKKKKRRGICSSKILWIRRFSGFFFAAMLMIILSPSLHSFPPAEAIKSSHLDNYLRLPSYQASHSSKYRFSFRKASGFRNADECGFTDHRITGVCNPSLVHVAITLDVVYLRGSMAAVHSILQHSLCPENILFHFIVSDADLETLVQSTIPQLKFKVYYFDPEIVRNLISSSVRQALEQPLNYARNYLADLLEPCVRRVIYLDSDLVVVDDITKLWITNLGSRTIGAPEYCHANFTNYFTGGFWSDQRFSRTFKGRKPCYFNTGVMVIDLVKWRRVGYTKRIERWMQIQKRYRIYELGSLPPFLLVFAGRVAPIEHRWNQHGLGGDNVKGSCRELHPGPVSLLHWSGSGKPWLRLDSNRPCPLDALWAPYDLYGHPH

>GhGATL5_At

MPKSPSLFDFHLLLLFTIFSLAAADAIAAARPITQQFKEAPQFYNSEICPKIVGEDDESDNGTSIFLCSDQSVHVAMTLDSAYIRGSMAAVLSILQHSSCPQNIVFHFVASATANASFLRATISSSFPYLNFQIYSFDDSSVSRLISTSIRSALDCPLNYARSYLADLLPSCVRRIVYLDSDLVLVDDIAKLAATQLGDNSVLAAPEHCNANFTSYFTLTFWSSATLSLTFANRKPCYFNTGVMVIDLDRWRRGDYTKKIEEWMEIQKRTRIYELGSLPPFLLVFAGNIVAVDHRWNQHGLGGDNFKGLCRDLHPGPVSLLHWSGKGKPWVRLDANRPCPLDALWAPYDLLQTPFALDS

>GhGATL13_Dt

MLWIMQFPGFFSAAMMMIVLSPSLQSFPPAEAIRSSHLDSYIRLPSFQVSTSPDSQDDRFSFRKAFEFRNADECGFADHKTTGVCDPTLVHAAITLDVQYLRGSIAAVHSILQHSLCPENVFFHFLVSETDMETLVRSTFPQLKFKVYYFDPEIVRNLISSSVRQALEQPLNYARNYLADLLEPCVRRVIYLDSDLVFVDNISKLWSTNLRSRTIGAPEYCHANFTKYFTNSFWSDDRFSGTFRGRKPCYFNTGVMVIDLVRWRRAGFTRRIERWMEIQKSNRIYELGSLPPFLLVFAGRVAPIEHRWNQHGLGGDNVRGSCRDLHPGPISLLHWSGSGKPWLRLDSKRPCPLDALWAPYDLYRHPH

>GhGATL11_At

MVALPFLGRLSFLLLIATTTTTAFAIGIHHGSIIRKPSSGVLNFREAPEFRNGDVCGTNASERIHIAMTLDVNYLRGTMAAVLSVLMHSTCPENTEFHFLWGKYEPEVLVSINSTFPYLNFRLYRFDSNRVRGKISRSIRQALDQPLNYARIYLADMLPPDVKRVLYLDSDLVVVDDIVKLWEVDLEGKVLAAPEYCHANFTKYFTDLFWSDKELSSTFNGRKPCYFNTGVMVVDVDKWRQGGYTPKVEEWMALQKQKRIYTLGSLPPFLLVLAGNIKAVHHRWNQHGLGGDNLEGKCRSLHPGPISLLHWSGKGKPWLRLDSRKPCVVDHLWAPYDLYGSSDHSLEE

>GhGATL13_At

MLWIIQFSGFFSAAMMMIVLSPSLQSFPPAEAIRSSHLDSYIRLPSFQVSTSPDSQDDRFSFRKAFEFRNADECGFADHQTTGVCDPTLVHVAITLDVQYLRGLIAAVHSILQHSLCPENVFFHFLVSETDMETLVRSTFPQLKFKVYYFDPEIVRNLISSSVRQALEQPLNYARNYLADLLEPCVRRVIYLDSDLVFVDDISKLWNTNLRSRTIGAPEYCHANFTKYFTNSFWSDDRFSGTFRGRKPCYFNTGVMVIDLVRWRRAGFTRRIERWMEIQKSNRIYELGSLPPFLLVFAGRVAPIEHRWNQHGLGGDNVRGSCRDLHPGPISLLHWSGSGKPWLRLDSKRPCPLDALWAPYDLYRHPH

>GhGATL9_Dt

MHPSKPLNLFFILTVMIRLCFADLPSFREAPAFRNGRECPQTTWSSLDKEIHNPSIIHIAMTLDTAYLRGSVAGVFSVLHHATCPENIVFHFVTTHRHGAKLTRAITSTFPYLNFHLYYFNTNLVKGKISSSIRRALDQPLNYARMYLADLLPAGVRRIIYFDSDLIVVDDVINLWSINLRSHVLGAPEYCHANFTNYFTSKFWSNPAFSASFKGRPRNPCYFNTGVMVIDLWKWREGKYTEKLENWMRIQKRYRIYELGSLPPFLLVFAGDVEGMEHRWNQHGLGGDNLEGLCRALHPGPVSLLHWSGKGKPWLRIDSKRPCPLDSLWAPYDLFRHPSLFSNS

>GhGATL15_Dt

MRSIFHAAAAVFLLHFFLTVTFSVGIRTIGGGDGSGPGFGFSEAPDYRNGVECPVSVNKEVVSSCDPDLVHVAMTLDSEYLRGSIAAVHSVLRHASCPENVFFHFIAAEFDPASPRVLSKLVRSTFPSLNFKIYIFREDAVINLISSSIRQALENPLNYARNYLGDILDLCVDRVIYLDSDLVVVDDIHKLWNTALTNSRVIGAPEYCHANFTKYFTDGFWSDPVLSRVFHSRRPCYFNTGVMVMDLVRWREGNYRKRIENWMEIQRKRRIYELGSLPPFLLVFAGNVEAIDHKWNQHGLGGDNVRGSCRSLHTGPVSLLHWSGKGKPWVRLDARNPCPLDHLWKPYDLYKGSSIKDRSSFPSSIFLGFSSFLS

>GhGATL2_Dt

MSKPQHYHFVLLLSTVVVALAAIANAAAATSITPRFKEAPSFYNSPDCPLIIDESHGFSILFSDQAVHVAMTLDAAYIRGTVAAILSILQHSSCPQNIAFHFVASADASLLRATISSSFPHLNFLVYPFCDSSVSRLISTSIRSALDCPLNYARSYLANLLPSCVHRVVYLDSDLVLIDDIAKLAATPLGDNSVLAAPEYCNANFTSYFTSTFWSNTYFSLTFANRKACYFNTGVMVIDLDRWREGDYTTKIEEWMEVQKRMRIYELGSLPPFLLVFAGNIVPVDHRWNQHGLGGDNFIGLCRDLHHGPVSLLHWSGKGKPWARLDANGPCPLDALWEPYDLLQPKTPFALDS

>GhGATL9_At

MHPSKPLNLFFILTVMIRLCFADLPSFREAPAFRNGRECPQTTWSSLDKEIHNPSIIHIAMTLDTAYLRGSVAGVFSVLHHATCPENIVFHFVTTHRHGAKLTRAITSTFPYLNFHLYYFNTNLVKGKISSSIRRALDQPLNYARMYLADLLPAGVRRIIYFDSDLIVVDDVINLWSINLRSHVLGAPEYCHANFTNYFTSKFWSNPAFAASFKGRPRNPCYFNTGVMVIDLWKWREGKYTEKLENWMRIQKRYRIYELGSLPPFLLVFAGDVEGMEHRWNQHGLGGDNLEGLCRALHPGPVSLLHWSGKGKPWLRIDSKRPCPLDSLWAPYDLFRHPSLFSNS

>GhGATL16_Dt

MFLSRSVLAFIFLSSLLLFPANSIRLFKAEDESDLFMEAPVYQNGPHCPVLAIHSVVKHGSCPQNVFFHFIASDSSSVVPTQLTRIVKSVFPSLSFKVYVFQKKLVRDLVSSSIRQALDNPLNYARIYLADLLETCIQKVIYLDSDTIVVDNIQKLWNINLTGSRTIGAPEYCNANFEKYFTNDFWSNPRFSKVFEGKRACYFNTGVMVMDLGRWRQGDYTREIEKWMRVQKDKRIYELGSLPPFLLVFGGDIEAIDHRWNQHGLGGDNLVNSCRTLHPGPISLLHWSGKGKPWVRLDAKRPCSVDFLWAPYDLYKFHGYSRHRQLDGFRFDSL

>GhGATL16_At

MFLSRSVLGFIFLSSLLLFPANSIRLFKAEDESDLFMEAPVYQNGPHCPVLAIHSVVKHGSCPQNVFFHFIASDSSLVVPTQLTRIVKSVFQSLSFKVYVFQKKLVSDLVSSSIRQALDNPLNYARIYLADLLETCIQKVIYLDSDTIVVDDIQKLWNINLTGSRTIGAPEYCNANFDKYFTSDFWSSPGFSKVFEGKRACYFNTGVMVMDLGRWRQGDYTREIEKWMRVQKDKRIYELGSLPPFLLVFGGDIEAIDHRWNQHGLGGDNLVNSCRTLHPGPVSLLHWSGKGKPWVRLDAKRPCSIDFLWAPYDLYKFHRYSRHRQLDGFRFDSL

>GhGATL15_At

MHSIFHAAAAVFLLHFFLTVTFSVGIRTIGGGDGSGPGFGFSEAPDYRNGVECPVSVNKEVVSSCDPDLVHVAMTLDSEYLRGSIAAVHSVLRHASCPENVFFHFIAAEFDPASPRVLSKLVRSTFPSLNFKIYIFREDAVINLISSSIRQALENPLNYARNYLGDILDLCVDRVIYLDSDLVVVDDIHKLWNTALTNSRVIGAPEYCHANFTKYFTDGFWSDPVLSRVFHSRRPCYFNTGVMVMDLVRWREGNYRKRIENWMEIQRKRRIYELGSLPPFLLVFAGNVEAIDHKWNQHGLGGDNVRGSCRSLHTGPVSLLHWSGKGKPWVRLDARNPCPLDHLWKPYDLYKGSSIKDRSSFPSSIFLGFSSYLS

>GhGATL3_At

MSKFALVIPPNKLHRHHHHRHHLFLLFLLISAVNAAPSLASTTTNNDQKFKEAPQFYNAPTCPSINGTTNEMCSHEAVHVAMTLDAAYSRGSMTAIFSILQHSSCPQNILFHFVSTSTADHRHLRLTITHSFPSLKFQIYPYDSSVVSGLISTSIRSALDSPLNYARNYLADLLPHCIHRIVYLDSDLVLVDDIAKLAATPLDENTVLAAPEYCNANFTSYFTPTFWSNPTLSLTFAGRKPCYFNTGVMVIDLQKWREGDYTIKIIEWMELQKRIRIYELGSLPPFLLVFAGTIAPVDHRWNQHGLGGDNYRGLCRNLHPGRVSLLHWSGKGKPWVRLDANRPCPLDALWAPYDLLQTPFALEP

>GhGATL3_Dt

MSKFALVIPPNHPHRHHHHRHHLFLLFLLISAINAAPSSATTTTNNDQKFKEAPQFYNAPTCPSINGTTNEMCSHEAVHVAMTLDAAYLRGSMAAIFSILQHSSCPQNILFHFVSTSTADHRHLLLTITHSFPSLKFQIYPYDSSVVSGLISTSIRSALDSPLNYARNYLADLLPHCIHDIVYLDSDLVLVDDIAKLAATPLDENTVLAAPEYCNANFTSYFTPTFWSNPTLSLTFAGRKPCYFNTGVMVIDLQKWREGDYTIKIIEWMELQKRIRIYELGSLPPFLLVFAGTIAPVDHRWNQHGLGGDNYRGLCRNLHPGRVSLLHWSGKGKPWVRLDANRPCPLDALWAPYDLLQTPFALES

>GhGATL12_Dt

MFPSKSFPPAEAIRSSHLHAYLRLPSYQVSSSTYSPLDRFSFRKASEFGNADKCRSTDHKITGVCDPSLVHVAITLDVEYLRGSIAAVHSILQHSLCPENVFFHFLVSDTDLETLVRSTFPQLKFKAYYFDPAIVRNLISSSVRQALEQPLNYARNYLADLLEPCVRRVIYLDSDLVVVDDIAKLWSTNLGSRTIGAPEYCHANFTKYFTSSFWSAERFSGTFKGRKPCYFNTGVMVIDLVKWRRVGYTKRIERWMEIQKTDRIYELGSLPPFLLVFAGRVAPIEHRWNQHGLGGDNVRGSCRGLHPGPVSLLHWSGSGKPWDRLDSRRPCPLDALWAPYDLYRHSH*

>ATGATL3

MSSLRLRLCLLLLLPITISCVTVTLTDLPAFREAPAFRNGRECSKTTWIPSDHEHNPSIIHIAMTLDAIYLRGSVAGVFSVLQHASCPENIVFHFIATHRRSADLRRIISSTFPYLTYHIYHFDPNLVRSKISSSIRRALDQPLNYARIYLADLLPIAVRRVIYFDSDLVVVDDVAKLWRIDLRRHVVGAPEYCHANFTNYFTSRFWSSQGYKSALKDRKPCYFNTGVMVIDLGKWRERRVTVKLETWMRIQKRHRIYELGSLPPFLLVFAGDVEPVEHRWNQHGLGGDNLEGLCRNLHPGPVSLLHWSGKGKPWLRLDSRRPCPLDSLWAPYDLFRYSPLISDS

>ATGATL9

MRLRFPMKSAVLAFAIFLVFIPLFSVGIRMIPGRLTAVSATVGNGFDLGSFVEAPEYRNGKECVSQSLNRENFVSSCDASLVHVAMTLDSEYLRGSIAAVHSMLRHASCPENVFFHLIAAEFDPASPRVLSQLVRSTFPSLNFKVYIFREDTVINLISSSIRQALENPLNYARNYLGDILDPCVDRVIYLDSDIIVVDDITKLWNTSLTGSRIIGAPEYCHANFTKYFTSGFWSDPALPGFFSGRKPCYFNTGVMVMDLVRWREGNYREKLETWMQIQKKKRIYDLGSLPPFLLVFAGNVEAIDHRWNQHGLGGDNVRGSCRSLHKGPVSLLHWSGKGKPWVRLDEKRPCPLDHLWEPYDLYEHKIERAKDQSLFGFSSLSELTEDSSFF

>ATGATL1

MSQHLLLLILLSLLLLHKPISATTIIQKFKEAPQFYNSADCPLIDDSESDDDVVAKPIFCSRRAVHVAMTLDAAYIRGSVAAVLSVLQHSSCPENIVFHFVASASADASSLRATISSSFPYLDFTVYVFNVSSVSRLISSSIRSALDCPLNYARSYLADLLPPCVRRVVYLDSDLILVDDIAKLAATDLGRDSVLAAPEYCNANFTSYFTSTFWSNPTLSLTFADRKACYFNTGVMVIDLSRWREGAYTSRIEEWMAMQKRMRIYELGSLPPFLLVFAGLIKPVNHRWNQHGLGGDNFRGLCRDLHPGPVSLLHWSGKGKPWARLDAGRPCPLDALWAPYDLLQTPFALDS

>ATGATL8

MSSRFSLTVVCLIALLPFVVGIRLIPARITSVGDGGGGGGNNGFSKLGPFMEAPEYRNGKECVSSSVNRENFVSSSSSSNDPSLVHIAMTLDSEYLRGSIAAVHSVLRHASCPENVFFHFIAAEFDSASPRVLSQLVRSTFPSLNFKVYIFREDTVINLISSSIRLALENPLNYARNYLGDILDRSVERVIYLDSDVITVDDITKLWNTVLTGSRVIGAPEYCHANFTQYFTSGFWSDPALPGLISGQKPCYFNTGVMVMDLVRWREGNYREKLEQWMQLQKKMRIYDLGSLPPFLLVFAGNVEAIDHRWNQHGLGGDNIRGSCRSLHPGPVSLLHWSGKGKPWVRLDEKRPCPLDHLWEPYDLYKHKIERAKDQSLLGFASLSELTDDSSFL

>ATGATL5

MHWITRFSAFFSAALAMILLSPSLQSFSPAAAIRSSHPYADEFKPQQNSDYSSFRESPMFRNAEQCRSSGEDSGVCNPNLVHVAITLDIDYLRGSIAAVNSILQHSMCPQSVFFHFLVSSESQNLESLIRSTFPKLTNLKIYYFAPETVQSLISSSVRQALEQPLNYARNYLADLLEPCVKRVIYLDSDLVVVDDIVKLWKTGLGQRTIGAPEYCHANFTKYFTGGFWSDKRFNGTFKGRNPCYFNTGVMVIDLKKWRQFRFTKRIEKWMEIQKIERIYELGSLPPFLLVFAGHVAPISHRWNQHGLGGDNVRGSCRDLHSGPVSLLHWSGSGKPWLRLDSKLPCPLDTLWAPYDLYKHSH

>ATGATL2

MHSKFILYLSILAVFTVSFAGGERFKEAPKFFNSPECLTIENDEDFVCSDKAIHVAMTLDTAYLRGSMAVILSVLQHSSCPQNIVFHFVTSKQSHRLQNYVVASFPYLKFRIYPYDVAAISGLISTSIRSALDSPLNYARNYLADILPTCLSRVVYLDSDLILVDDISKLFSTHIPTDVVLAAPEYCNANFTTYFTPTFWSNPSLSITLSLNRRATPCYFNTGVMVIELKKWREGDYTRKIIEWMELQKRIRIYELGSLPPFLLVFAGNIAPVDHRWNQHGLGGDNFRGLCRDLHPGPVSLLHWSGKGKPWVRLDDGRPCPLDALWVPYDLLESRFDLIES

>ATGATL4

MASRSLSYTQLLGLLSFILLLVTTTTMAVRVGVILHKPSAPTLPVFREAPAFRNGDQCGTREADQIHIAMTLDTNYLRGTMAAVLSLLQHSTCPENLSFHFLSLPHFENDLFTSIKSTFPYLNFKIYQFDPNLVRSKISKSIRQALDQPLNYARIYLADIIPSSVDRIIYLDSDLVVVDDIEKLWHVEMEGKVVAAPEYCHANFTHYFTRTFWSDPVLVKVLEGKRPCYFNTGVMVVDVNKWRKGMYTQKVEEWMTIQKQKRIYHLGSLPPFLLIFAGDIKAVNHRWNQHGLGGDNFEGRCRTLHPGPISLLHWSGKGKPWLRLDSRKPCIVDHLWAPYDLYRSSRHSLEE

>ATGATL7

MLWIMRFSGLFSAALVIIVLSPSLQSFPPAEAIRSSHLDAYLRFPSSDPPPHRFSFRKAPVFRNAADCAAADIDSGVCNPSLVHVAITLDFEYLRGSIAAVHSILKHSSCPESVFFHFLVSETDLESLIRSTFPELKLKVYYFDPEIVRTLISTSVRQALEQPLNYARNYLADLLEPCVRRVIYLDSDLIVVDDIAKLWMTKLGSKTIGAPEYCHANFTKYFTPAFWSDERFSGAFSGRKPCYFNTGVMVMDLERWRRVGYTEVIEKWMEIQKSDRIYELGSLPPFLLVFAGEVAPIEHRWNQHGLGGDNVRGSCRDLHPGPVSLLHWSGSGKPWFRLDSRRPCPLDTLWAPYDLYGHYSR

>ATGATL10

MMSGSRLASRLIIIFSIISTSFFTVESIRLFPDSFDDASSDLMEAPAYQNGLDCSVLAKNRLLLACDPSAVHIAMTLDPAYLRGTVSAVHSILKHTSCPENIFFHFIASGTSQGSLAKTLSSVFPSLSFKVYTFEETTVKNLISSSIRQALDSPLNYARSYLSEILSSCVSRVIYLDSDVIVVDDIQKLWKISLSGSRTIGAPEYCHANFTKYFTDSFWSDQKLSSVFDSKTPCYFNTGVMVIDLERWREGDYTRKIENWMKIQKEDKRIYELGSLPPFLLVFGGDIEAIDHQWNQHGLGGDNIVSSCRSLHPGPVSLIHWSGKGKPWVRLDDGKPCPIDYLWAPYDLHKSQRQYLQYNQELEIL

>ATGATL6

MLWITRFAGLFSAAMAVIVLSPSLQSFPPAAAIRSSPSPIFRKAPAVFNNGDECLSSGGVCNPSLVHVAITLDVEYLRGSIAAVNSILQHSVCPESVFFHFIAVSEETNLLESLVRSVFPRLKFNIYDFAPETVRGLISSSVRQALEQPLNYARSYLADLLEPCVNRVIYLDSDLVVVDDIAKLWKTSLGSRIIGAPEYCHANFTKYFTGGFWSEERFSGTFRGRKPCYFNTGVMVIDLKKWRRGGYTKRIEKWMEIQRRERIYELGSLPPFLLVFSGHVAPISHRWNQHGLGGDNVRGSCRDLHPGPVSLLHWSGSGKPWIRLDSKRPCPLDALWTPYDLYRHSH

>GrGATL6

MPKPQLLHFLHVLSFNVFAIAAVIATVTAATVTLQLKEAPQFYNSPDCPSITNIEDDPNGESSIFCSDQAVHVAVTLDSPYIRGSMAVTLSVLQHSSCPQNIVFHFVTSAATNVSFLHATISASFPYLNFQIYPFDDASVSRLISTSIRSALDCPLNYARSYLANLLPLCVTRVVYLDSDLILVDDIAKLAATPLGDNSVLAAPQYCNANFTSYFTTTFWSNPSLYLTFANRKPCYFNTGVMVMDLDRWRNGDYTTKIEEWMEIQKQMRIYELGSLPPFLLVFAGNIVPVDHRWNQHGLGGDNFRGLCRNLHPGPVSLLHWSGKGKPWARLDANRPCPLDALWAPYDLLQTPFVLDS*

>GrGATL17

MRFILYAAALLLLHFLLTVSFCLAIRTTVGGDGLGFRFPEAPYYRNGVECPVSSGDRNLVQVAMTLDFEYLRGSIAAVHSVVRHASCPENIFFHFIAAEFDPASPRVLSKLVRSTFPSLNFRIYIFREDTVINLISSSIRQALENPLNYARNYLGDMLDLQVGRVIYLDSDLVLVDDILKLWNTTLTNSRVIGAPEYCHANFTKYFTAGFWSDPVISRVFRSRKPCYFNTGVMVMDLVRWREGNYRKRIENWMEIQRKKKIYELGSLPPYLLVFAGNVEGIDHRWNQHGLGGDNIRGSCRSLHPGPVSLLHWSGKGKPWVRLDGGSPCPLDHIWESYDLYKGNLIKHQSFPSAFANFFEYPSYLF*

>GrGATL5

MPKSPSLFDFHLLLLFTIFSLAAADAIAAARPITQQFKEAPQFYNSEICPKIVGEDDESDNGTSVFLCSGQSVHVAMTLDSAYIRGSMAAVLSILQHSSCPQNIVFHFVASATANASFLHATISSSFPYLNFQVYSFDDSSVSRLISTSIRSALDCPLNYARSYLADLLPSCVGRIVYLDSDLVLVDDIAKLAATQLGDNSVLAAPEHCNANFTSYFTLTFWSSPTLSLTFANRKPCYFNTGVMVIDLDRWRRGDYTKKIEEWMEIQKRTRIYELGSLPPFLLVFAGNIVAVDHRWNQHGLGGDNFKGLCRDLHPGPVSLLHWSGKGKPWVRLDANRPCPLDALWAPYDLLQTPFALDS*

>GrGATL4

MSKPPRFDLLLVLLFTVIALASDSVTATPITQLFKEAPQFYNSPDCAVLVDEDESDGEASILCSDQAVHVAMTLDTAYIRGSMAAILSVLQHSSCPQNIAFHFVASATANATFLRATISSSFPYLNFRVYPFDDSSVSRLISTSIRSALDCPLNYARSYLANLLPPCVRRVVYLDSDLVLVDDIAKLAATPLGDDSVLAAPEHCNANFTTYFTPTFWSNPSLSLTFANRKPCYFNTGVMVIDLDRWREGDYTTKIEEWMELQKRIRIYELGSLPPFLLVFAGNIVPVDHRWNQHGLGGDNFRGLCRDLHPGPVSLLHWSGKGKPWARLDANRPCPLDALWAPYDLLETPFALDA*

>GrGATL9

MHPSKPLNLFFILTVMIRLCFADLPSFREAPAFRNGRECPQTTWSSLDKEIHNPSIIHIAMTLDTAYLRGSVAGVFSVLHHATCPENIVFHFVTTHRHGAKLTRAITSTFPYLNFHLYYFNTNLVKGKISSSIRRALDQPLNYARMYLADLLPAGVRRIIYFDSDLIVVDDVINLWSINLRSHVLGAPEYCHANFTNYFTSKFWSNPAFSASFKGRPRNPCYFNTGVMVIDLWKWREGKYTEKLENWMRIQKRYRIYELGSLPPFLLVFAGDVEGMEHRWNQHGLGGDNLEGLCRALHPGPVSLLHWSGKGKPWLRIDSKRPCPLDSLWAPYDLFRHPSLFSNS*

>GrGATL11

MVALPFLGRLSFLLLIATTTTTAFAIGIHHGSIIRKPSSGVSNFREAPKFRNGDVCGTNASERIHIAMTLDVNYLRGTMAAVLSVLMHSTCPENTEFHFLWGKYEPEVLVSINSTFPYLNFRLYHFDSDRVRGKISRSIRQALDQPLNYARIYLADMLPPDVKRVLYLDSDLVVVDDIVKLWEVDLEGKVLAAPEYCHANFTKYFTDLFWSDKELSSTFNGRKPCYFNTGVMVVDVDKWRQGGYTPKVEEWMALQKQKRIYTLGSLPPFLLVLAGNIKAVHHRWNQHGLGGDNLEGKCRSLHPGPISLLHWSGKGKPWLRLDSRKPCAVDHLWAPYDLYGSPDHSLEE*

>GrGATL7

MPEPHGHLHRHHLLLLLFLLITATNSAPASATTTNYQIFKEAPQFYNSPACPSLDTNEMCSHQAVHVAMTLDAAYLRGSMAAILSVLQHSSCPQNIRFHFIPSSTANHQHLRLTISRSFPSLKFQIYPYDSSKVSGLISTSIRSALDCPLNYARNYLANLLPPCLSRVVYLDSDLVLVDDISKLAATPLGDRSVLAAPEYCNANFTSYFTPTFWSNPTLSLTFAGRKACYFNTGVMVIDLQRWRQGDYTTKIIEWMELQKRMRIYELGSLPPFLLVFAGNIAPVDHRWNQHGLGGDNYRGLCRNLHPGPVSLLHWSGKGKPWVRLDANRPCPLDALWAPYDLLQTPFALES*

>GrGATL13

MLWIMQFSGFFSAAMMMIVLSPSLQSFPPAEAIRSSHLDSYIRLPSFQVSISPDSQDDRFSFRKAFEFRNADECGFADHKTTGVCDPTLVHVAITLDVQYLRGSIAAVHSILQHSLCPENVFFHFLVSETDMETLVRSTFPQLKFKVYYFDPEIVRNLISSSVRQALEQPLNYARNYLADLLEPCVRRVIYLDSDLVFVDNISKLWSTNLRSRTIGAPEYCHANFTKYFTNSFWSDDRFSGTFRGRKPCYFNTGVMVIDLVRWRRAGFTRRIERWMEIQKSNRIYELGSLPPFLLVFAGRVAPIEHRWNQHGLGGDNVRGSCRDLHPGPISLLHWSGSGKPWLRLDSKRPCPLDALWAPYDLYRHPH*

>GrGATL1

MRRNHLSYPNSLFFTFNINRLFRYRVNITCIYIDSVLMMKVICTMAHLLHRLLIFTILIAMAATVTPITHQHFKEASKFYNSAACLSISEPYILCSHQALHVAMTIDKAYIRGSMAAILSVLQHSSCPHNIAFHFLISATANASLLRATISSSFPYLYFRLYPFDHSSVAPLISTSIRSALDCPLNYARIYLASLLPSCIHRVLYLDSDLILVDDIAELAAIQLGDNSVLAATEYCSANFASYFTPTFWSNPCLSLTFANRKACYFNTGVMVMDLDRWREGDYTRKIEEWMEIQKRTRIYELGSLPPFLLVFAGNIVPVDHRWNQHGLGGDNFDGLCRDLHPGPVSVLHWSGKGKPWARLDAKTPCLLDALWASYDLLDTSFAFDS*

>GrGATL3

MSKFALVIPPNHLHRHHHHHLFLLFLLISAINAVPSSATTTTNNDQKFKEAPQFYNAPTCPSINGTTNEMCSHEAVHVAMTLDAAYLRGSMAAIFSILQHSSCPQNILFHFVSTSTADHRHLLLTITHSFPSLKFQIYPYDSSVVSGLISTSIRSALDSPLNYARNYLADLLPHCIHRIVYLDSDLVLVDDIAKLAATPLDENTVLAAPEYCNANFTSYFTPTFWSNPTLSLTFAGRKPCYFNTGVMVIDLQKWREGDYTIKIIEWMELQKRIRIYELGSLPPFLLVFAGTIAPVDHRWNQHGLGGDNYRGLCRNLHPGRVSLLHWSGKGKPWVRLDANRPCPLDALWAPYDLLQTPFALES*

>GrGATL15

MRSIFHAAAAVFLLRFFLTVTFSVGIRTIGGDGSGPGFGFSEAPDYRNGVECPVSVNKEVVSSCDPDLVHVAMTLDSEYLRGSIAAVHSVLRHASCPENVFFHFIAAEFDPASPRVLSKLVRSTFPSLNFKIYIFREDAVINLISSSIRQALENPLNYARNYLGDILDLCVDRVIYLDSDLVVVDDIHKLWNTALTNSRVIGAPEYCHANFTKYFTDGFWSDPVLSRVFHSRRPCYFNTGVMVMDLVRWREGNYRKRIENWMEIQRKRRIYELGSLPPFLLVFAGNVEAIDHKWNQHGLGGDNVRGSCRSLHTGPVSLLHWSGKGKPWVRLDARNPCPLDHLWKPYDLYKGSSIKDRSSFPSSIFLGFSSYLS*

>GrGATL2

MSKPQHFHFVLLLSTVVVALAAIANAAAATSITPRFKEAPSFYNSPDCPLIIDENHGFSILFSDQAVHVAMTLDTAYIRGTVAAILSILQHSSCPQNIAFHFVYYERAILRRATISSSFPHLNFLVYPFYDSSVSRLLSTSIRSALDCPLNYARSYLANLLPSCVHRVVYLDSDLVLIDDIAKLAATPLGDNSVLAAPEYCNANFTSYFTSTFWSNTYFSLTFANRKACYFNTGVMVIDLDRWREGDYTTKIEEWMEVQKRMRIYELGSLPPFLLVFAGNIVPVDRRWNQHGLGGDNFIGLCRDLHHGPVSLLHWSGKGKPWARLDANRPCPLDALWEPYDLLQPKTPFALDS*

>GrGATL10

MALWRTLSIPIASPHGLILSFLLLLHSAVTTTVAIRVRTIIHDPSPVKLPVFREAPAFRNGDSCGSNKDDRIHIAMTLDENYLRGTMAAVLSMLQHSTCPENLSFHFLCAHSDVKLVSSIESTFPYLNFKIYRFDSNRVRGKISKSIRQALDQPLNYARIYLADILPADVKRVLYLDSDLVVVDDIGKLWDVDMEDKVLAAPEYCHANFTVYFNDAFWSDPVLSNTFQGRNPCYFNTGVMVVDVDKWRKGGYTKKVEQWMAFHKKKRIYHLGSLPPFLLVLAGNIKPVDHRWNQHGLGGDNFEGKCRNLHPGPISLLHWSGKGKPWLRLDSRKPCVVDHLWAPYDLYRSSRHFLEE*

>GrGATL14

MVDLKRILLFFRSSLFILLADWLDFKSKKKKRRGICSSKILWIRRFSGFFFAAMLMIILSPSLHSFPPAEAIKSSHLDSYLRLQSYQASHSSKYRFSFRKASGFRNADECGFTDRKITGVCDPSLVHVAITLDVVYLRGSMAAVHSILQHSLCPENIFFHFIVSDADLETLVRSTIPQLKFKVYYFDPETVRNLISSSVRQALEQPLNYARNYLADLLEPCVRRVIYLDSDLVVVDDITKLWITNLGSRTIGAPEYCHANFTNYFTGGFWSDQRFSRTFKGRKPCYFNTGVMVIDLVKWRRVGYTKRIERWMQIQKRYRIYELGSLPPFLLVFAGRVAPIEHRWNQHGLGGDNVKGSCRELHPGPVSLLHWSGSGKPWLRLDSNRPCPLDALWAPYDLYGHPH*

>GrGATL8

MPKLALIPPHDHHRHHRQLLLIFLLISATTAAPASATTNYQVFKEAPQFYNSPTCPSIHTNGMCCDQAVHVAMALDTAYLRGLMAAILSILQHSSCPQNILFHFIASATANHQHFRYTIFRAFPALVFQIYPYDSKAVSGLISTSIRSALDCPLNYARNYLPNLLPRCLRRVVYLDSDLVLVDDMAKLAATPLGDSSVLAAPEYCNANFTSYFTPTFWSNPTLSLTFSGRKACYFNTGVMVIDLQRWRAGDYTTKIVEWMELQKRMRIYELGSLPPFLLVFAGNIAPVDHRWNQHGLGGDNYRGLCRDLHPGPTSLLHWSGKGKPWARLDANRPCPLDAFWAPYDLLQTSSALES*

>GrGATL12

MLWVMQFSGFFSAAMLMIVLSPSFQSFPPAEAIRSSHLHAYLRLPSYQVSSSTYSPLDRFSFRKASEFGNADKCRSTDHKITGVCDPSLVHVAITLDVEYLRGSIAAVHSILQHSLCPENVFFHFLVSDTDLETLVRSTFPQLKFKAYYFDPAIVRNLISSSVRQALEQPLNYARNYLADLLEPCVRRVIYLDSDLVVVDDIAKLWSTNLGSRTIGAPEYCHANFTKYFTSGFWSAERFSGTFKGRKPCYFNTGVMVIDLVKWRRVGYTKRIERWMEIQKTDRIYELGSLPPFLLVFAGRVAPIEHRWNQHGLGGDNVRGSCRGLHPGPVSLLHWSGSGKPWDRLDSRRPCPLDALWAPYDLYRHSH*

>GrGATL16

MFLSRFVLAFIFLSSLLLFPANSIRLFKAEDESDLFMEAPVYQNGPHCPVLAIHSVVKHGSCPQNVFFHFIASDSSSVVPTQLTRIVKSVFPSLSFKVYVFQKKLVRDLVSSSIRQALDNPLNYARIYLADLLETCIQKVIYLDSDTIVVDNIQKLWNINLTGSRTIGAPEYCNANFDKYFTSDFWSNPRFSKVFEGKRACYFNTGVMVMDLGRWRQGDYTREIEKWMRVQKDKRIYELGSLPPFLLVFGGDIEAIDHRWNQHGLGGDNLVNSCRTLHPGPISLLHWSGKGKPWVRLDAKRPCSVDFLWAPYDLYKFHGYSRHRQLDGFRFDSL*

>GaGATL1

MVGDPWPLFFPYFNTPLVPTTLPSISSSPPPQTPHFYALYPFDHSSVAPLISTSIRSALDCPLNYARIYL

ASLLPSCIHRVLYLDSDLILVDDVAELAAIQLGDNSVLAATEYCSANFVSYFTPTFWSNPCLSLTFANRK

ACYFNTGVMVMDLDRWREGDYTRKIEEWMEIQKRMRIYELGSLPPFLLVESTWAWGDNFDGLCRDLHPGPVSVLHWSGKGKPWARLDAKTPCLLDALWASYDLMDTSFAFDS

>GaGATL3

MSKFALVIPANQLHRHHHHHHRHHLFLLFLLISAVNAAPSLASTTTNNDQKFKEAPQFYNAPTCPSINGTTNEMCSHEAVHVAMTLDAAYSRGSMAAIFSILQHSSCPQNILFHFVSTSTADHRHLRLTITHSFPSLKFQIYPYDSSVVSGLISTSIRSALDSPLNYARNYLADLLPHCIHRIVYLDSDLVLVDDIAKLAATPLDENTVLAAPEYCNANFTSYFTPTFWSNPTLSLTFAGRKPCYFNTGVMVIDLQKWREGDYTTKIIEWMELQKRIRIYELGSLPPFLLVFAGTIAPVDHRWNQHGLGGDNYRGLCRNLHPGRVSLLHWSGKGKPWVRLDANRPCPLDALWAPYDLLQTPFALES

>GaGATL15

MHSIFHAAAAVFLLHFFLTVTFSVGIRTIGGGDGSGPGFGFSEAPDYRNGVECPVSVNKEVVSSCDPDLVHVAMTLDSEYLRGSIAAVHSVLRHASCPENVFFHFIAAEFDPASPRVLSKLVRSTFPSLNFKIYIFREDAVINLISSSIRQALENPLNYARNYLGDILDLCVDRVIYLDSDLVVVDDIHKLWNTALTNSRVIGAPEYCHANFTKYFTDGFWSDPVLSRVFHSRRPCYFNTGVMVMDLVRWREGNYRKRIENWMEIQRKRRIYELGSLPPFLLVFAGNVEAIDHKWNQHGLGGDNVRGSCRSLHTGPVSLLHWSGKGKPWVRLDARNPCPLDHLWKPYDLYKGSSIKDRSSFPSSIFLGFSSYLS

>GaGATL16

MFLSRSVLGFIFLSSLLLFPANSIRLFKAEDESDLFMEAPVYQNGPHCPVLGKDTLICDSSIVHIAMTIDPEYLRGTIAAIHSVVKHGSCPQNVFFHFIASDSSLVVPTQLTRIVKSVFQSLSFKVYVFQKKLVSDLVSS

SIRQALDNPLNYARIYLADLLETCIQKVIYLDSDTIVVDDIQKLWNINLTGSRTIGAPEYCNANFDKYFT

SDFWSSPGFSKVFEGKRACYFNTGVMVMDLGRWRQGDYTREIEKWMRVQKDKRIYELGSLPPFLLVFGGDIEAIDHRWNQHGLGGDNLVNSCRTLHPGPVSLLHWSGKGKPWVRLDAKRPCSIDFLWAPYDLYKFHGYSRHRQLDGFRFDSL

>GaGATL17

MRFILYAAALLLLHFLLTVWFCLAIRTTVGGDGLGFRFPEAPYYRNGVECPVSSGDRNLVQVAMTLDFEYLRGSIAAIHSVVRHASCPENIFFHFIAAVFDPASPRVLSKLVRSTFPSLNFRIYIFREDTVINLISSSIRQALENPLNYARNYLGDMLDLQVDRVIYLDSDLVLVDDILKLWNTTLTNSRVIGAPEYCHANFTKYFTAGFWSDPVISRVFRSRKPCYFNTGVMVMDLVRWREGNYRKRIENWMEIQRKKKIYELGSLPPYLLVFAGNVEGIDHRWNQHGLGGDNIRGSCRSLHPGPVSLLHWSGKGKPWVRLDGGNPCPLDHIWESYDLYKGNLIKHQSFPSAFANFFEYPSYLF

>GaGATL5

MPKSPSLFDFHLLLLFTIFSLAAADAIAAARPITQQFKEAPQFYNSEICPKIVGEDDESDNGTSIFLCSD

QSVHVAMTLDSAYIRGSMAAVLSILQHSSCPQNIVFHFVASATANASFLRATISSSFPYLNFQIYSFDDSSVSRLISTSIRSALDCPLNYARSYLADLLPSCVRRIVYLDSDLVLVDDIAKLAATQLGDNSVLAAPEHCNANFTSYFTLTFWSSATLSLTFANRKPCYFNTGVMVIDLDRWRRGDYTKKIEEWMEIQKRTRIYELGSLPPFLLVFAGSIVAVDHRWNQHGLGGDNFKGLCRDLHPGPVSLLHWSGKGKPWVRLDANRPCPLDALWAPYDLLQMPFALDS

>GaGATL9

MHPSKPLNLFFILTVMIRLCFADLPSFREAPAFRNGRECPQTTWSSLDKEIHNPSIIHIAMTLDTAYLRG

SVAGVFSVLHHATCPENIVFHFVTTHRHGAKLTRAITSTFPYLNFHLYYFNTNLVKGKISSSIRRALDQP

LNYARMYLADLLPAGVRRIIYFDSDLIVVDDVINLWSINLRSHVLGAPEYCHANFTNYFTSKFWSNPAFAASFKGRPRNPCYFNTGVMVIDLWKWREGKYTEKLENWMRIQKRYRIYELGSLPPFLLVFAGDVEGMEHRWNQHGLGGDNLEGLCRALHPGPVSLLHWSGKGKPWLRIDSKRPCPLDSLWAPYDLFRHPCLFSNS

>GaGATL4

MSKPPRFDLLLVLLFTVIALASNSVTVATPITQQFKEAPQFYNSPDCAVPVDEDESDGEASILCSDQAVHVAMTLDTAYIRGSMAAILSVLQHSSCPQNIAFHFIASATANASFLRATISSSFPYLNFRVYPFDDSSVSRLISTSIRSALDCPLNYARSYLANLLPPCVRRVVYLDSDLVLVDDIAKLAATPLGDDSVLAAPEHCNANFTTYFTPTFWSNPSLSLTFANRKPCYFNTGVMVIELDRWREGDYTTKIEEWMELQKRIRIYELGSLPPFLLVFAGNIVPVDHRWNQHGLGGDNFRGLCRDLHPGPVSLLHWSGKGKPWARLDANRPCPLDALWAPYDLLQTPFALDS

>GaGATL6

MPKSQLLHFLHVLAFNVFAIAAVIATVTAATVTLQLKEAPQFYNSPDCPSITNIEDDPNGESSIFCSDQAVHVAVTLDSAYIRGSMAVILSVLQHSSCPQNIVFHFVTSAAANVSFLHATISSSFPYLNFQIYPFDDASVSRLISTSIRSALDCPLNYARSYLANLLPLCVTRVVYLDSDLILVDDIAKLAATPLGDNSVLAAPQYCNANFTSYFTTTFWSNPSLSLTFANRKPCYFNTGVMVMDLDRWRNGDYTTKIEEWMEIQKQMRIYELGSLPPFLLVFAGNIVPVDHRWNQHGLGGDNFRGLCRNLHPGPVSLLHWSGKGKPWARLDANRPCPLDALWAPYDLLQTPFVLDS

>GaGATL12

MLWVMQFSGFFSAAMLMIVLSPSFQSFPPAEAIRSSHLHAYLRLSSYQVSSSPYSPLDRFSFRKASEFGNADKCRSTDHKITGVCDPSLVHVAITLDVEYLRGSIAAVHSILQHSLCPENVFFHFLVSDTDLETLVRSTFPQLKFKAYYFDPAIVRNLISSSVRQALEQPLNYARNYLADLLEPCVRRVIYLDSDLVVVDDIAKLWSTNLGSRTIGAPEYCHANFTKYFTSGFWSAERFSGTFKGRKPCYFNTGVMVIDLVKWRRVGYTKRIERWMEIQKTDRIYELGSLPPFLLVFAGRVAPIEHRWNQHGLGGDNVRGSCRGLHPGPVSLLHWSGSGKPWDRLDSRRPCPLDALWAPYDLYRHSH

>GaGATL10

MALWRTLPIPIASPHGLILSFLILLHSAVTTTVAIRVRTIIHDPSPVKLPVFREAPAFRNGDSCGSNKDD

RIHIAMTLDENYLRGTMAAVLSMLQHSTCPENLSFHFLCAHSDVELVSSIESTFPYLNFKIYRFDSNRVR

GKISKSIRQALDQPLNYARIYLADILPADVKRVLYLDSDLVVVDDIGKLWDVDMEDKVLAAPEYCHANFTVYFNDAFWSDPVLSNTFQGRNPCYFNTGVMVVDVDKWRKGGYTKKVEQWMAFQKKKRIYHLGSLPPFLLVLAGNIKPVDHRWNQHGLGGDNFEGKCRNLHPGPISLLHWSGKGKPWLRLDSRKPCVVDHLWAPYDLYRSSRHFLEE

>GaGATL7

MPKPHDHLHRHHLLLLLFLLITATNSAPASATTTNYQRFKEAPQFYNSPACPSLDTNEMCSHQAVHAAMTLDAAYLRGSMAAILSVLQHSSCPQNIRFHFIPSATANHHHLRLTISRSFPSLKFQIYPYDSSKVSGLISTSIRSALDCPLNYARNYLANLLPPCLSRVVYLDSDLVLVDDIAKLAATPLGDRSVLAAPEYCNANFTSYFTPTFWSNPTLSLTFAGRKACYFNTGVMVIDLQRWRQGDYTTKIIEWMELQKRIRIYELGSLPPFLLVFAGNIAPVDHRWNQHGLGGDNYRGLCRNLHPGPVSLLHWSGKGKPWVRLDANRPCPLDALWAPYDLLQTPFALES

>GaGATL11

MVALPFLGRLSFLLLIATTTTTAFAIGIHHGSIIRKPSSGVPNFREAPEFRNGDVCGTNASERIHIAMTL

DVNYLRGTMAAVLSVLMHSTCPENTEFHFLWGKYEPEVLVSINSTFPYLNFRLYRFDSNRVRGKISRSIRQALDQPLNYARIYLADMLPPDVKRVLYLDSDLVVVDDIVKLWEVDLEGKVLAAPEYCHANFTKYFTDLFWSDKELSSTFNGRKPCYFNTGVMVVDVDKWRQGGYTPKVEEWMALQKQKRIYTLGSLPPFLLVLAGNIKAVHHRWNQHGLGGDNLEGKCRSLHPGPISLLHWSGKGKPWLRLDSRKPCVVDHLWAPYDLYGSSDHSLEE

>GaGATL13

MLWIIQFSGFFSAAMVMIVLSPSLQSFPPAEAIRSSHLDSYIRLPSFQVSTSPDSQDDRFSFRKAFEFRN

ADECGFADHQTTGVCDPTLVHVAITLDVQYLRGLIAAVHSILQHSLCPENVFFHFLVSETDMETLVRSTFPQLKFKVYYFDPEIVRNLISSSVRQALEQPLNYARNYLADLLEPCVRRVIYLDSDLVFVDDISKLWNTNLRSRTIGAPEYCHANFTKYFTNSFWSDDRFSGTFRGRKPCYFNTGVMVIDLVRWRRAGFTRRIERWMEIQKSNRIYELGSLPPFLLVFAGRVAPIEHRWNQHGLGGDNVRGSCRDLHPGPISLLHWSGSGKPWLRLDSKRPCPLDALWAPYDLYRHPH

>GaGATL14

MADLKRILLFFRSSLFILLADWLDFKSKKKKRRGICSSKILWIRRFSGFFFAAMLMIILSPSLHSFPPAE

AIKSSHLDSYLRLPSYQASHSSKDRFSFRKASGFRNADECGFTDRKITGVCDPSLVHVAITLDVVYLRGS

IAAVHSILQHSLCPENIFFHFIVSDADLETLVRSTIPQLKFKVYYFDPEIVRNLISSSVRQALEQPLNYA

RNYLADLLEPCVRRVIYLDSDLVVVDDISKLWITNLGSRTIGAPEYCHANFTNYFTGGFWSDQRFSRTFKGRKPCYFNTGVMVIDLVKWRRVGYTKRIERWMQIQKRYRIYELGSLPPFLLVFAGRVAPIEHRWNQHGLGGDNVKGSCRELHPGPVSLLHWSGSGKPWLRLDSNRPCPLDALWAPYDLYGHPH

>GaGATL8

MAAILSILQHSSCPQNILFHFIASDNANHHHFRYTISRAFTALVFQIYPYDSKAVSGLISTSIRSALDCP

LNYARNYLPNLLPHCLRRVVYLDSDLVLVDDMAKLTATPLGDSSVLAAPEYCNANFTSYFTPTFWSNPTLSLTFSGRNACYFNTGVMVIDLQRWRAGDYTTKIVEWMELQKRMRIYELGSLPPFLLVFAGNIAPVDHRWNQHGLGGDNYRGLCRDLHPGPTSLLHWSGKGKPWARIDANRPCPLDAFWAPYDLLQTSSSLES

>Zm1d022349

MARACCRMAAAPAAALLLLLVAAASAIRVDVVRISSSSSAPPQPPSPPAFREAPAFRNGDECPPRGSPDGHVDVAMTLDANYLRGTMAAVFSILQHTACPENVAFHFLAAAADPDSDSDPDPLAAIRATFPYLDPSVHRFDPSRVRGRISRSVRHALDQPLNYARIYLADTLPAVVRRVIYLDSDVVVVDDVRKLWSVDLGERHVVAAPEYCHANFTKYFTDAFWSDRELRAAFRDRRPCYFNTGVMVMDVARWRRGGYTRRVEEWMAVQKRKRIYHLGSLPPFLLVLAGDIRPVDHRWNQHGLGGDNVEGRCRSLHPGPISLLHWSGKGKPWLRLDSRKPCTVDYLWAPYDLYKAAATALEE*

>Zm1d025917

MRVLAVVLLAAACAAAAAGGGGELPEFREAPAFRNGAACAGAPTIHIAMTLDATYLRGSLAGVLSVLRHAACPESIAFHFVASSASPARRLASLRRALAAAFPTLPATVHRFDARLVRGKISSSVRRALDQPLNYARIYLADLLPRSVPRVLYLDSDLLVVDDVARLWATDLGPDAALAAPEYCHANFTSYFTDAFWRHPEYAAVFANRTRAPCYFNTGVMVIDLDRWRSGGYTAKLEYWMEVQKQEARIYELGSLPPFLLVFAGEVKAVGHRWNQHGLGGDNVAGQCRELHPGPVSLLHWSGKGKPWLRLDAGRPCPLDALWAPYDLLRRRGARDDLLDAVA*

>Zm1d002851

MRVLAVFLLGAAFAAAAGGGGELPEFREAPAFRNGAACADAPTIHIAMTLDGTYLRGSLAGVLSVLRHAACPESVAFHFVASSASPARRLASLRRALAAAFPTLPATVHRFDARLVRGKISSSVRRALDQPLNYARIYLADLLPRSVSRVLYLDSDLLVVDDVARLWATDLGPDAALAAPEYCHANFTSYFTDAFWSHPEYTAVFANRTRVPCYFNTGVMVIDLDRWRSGGYTAKLEYWMEVQKQEARIYELGSLPPFLLVFAGEVKAVEHRWNQHGLGGDNVAGQCRQLHPGPVSLLHWSGKGKPWLRLDAGRPCPLDALWMPYDLLRRRGARDDLLAAVS*

>Zm1d033385

MTRSPLALALLLLCAAGAAAAVPRYREAPHFTNSAAAQCPPLLQPSDADAACSPHAAVHVAMTLDASYLRGTMAAVLSVLRHASCPESIHFHFIASASSGSSATAEALRATVRASFPSLAFRVYPFADEARVAGLISTSIRGALDRPLNYARSYLASTLPACVRRVVYLDSDVVLTDDIAALAATPLPGEGTAVAAPQYCGANFTAYFTPGFWASPALSSAFAGRRACYFNTGVMVLDLARWRRAGYTAQIEEWMELQKRVRIYELGSLPPFLLVFAGRIASVDHRWNQHGLGGDNYRGLCRGLHAGAVSLLHWSGKGKPWDRLDAGRPCPLDAVWAKYDLLRPAAGIETS*

>Zm1d029235

MMIRQPSSSSSVLMFREAPAFRNGPDCGADGRVDIAMTLDANYLRGTMAAVLSILQHTACPESVAFHFLTADADADGHGLSAALRASFPFLDLRVYRFDPSRVRDRISRSVRQELDQPLNYARVYLADTLPRDVRRVTYLDSDVVVVDDVRTLASVDLAGHVVAAPEYCHANFSNYFTDAFWSHPALNGTFHGRRPCYFNTGVMVMDVDKWRAGGYTRRVEEWMAVQKRRRIYHLGSLPPFLLVFAGHIRAVDHRWNQHGLGGDNVEGRCRGLHPGPISLLHWSGKGKPWLRLDARRPCSVDYLWAPYDLYRYSSPVIDEW*

>Zm1d028824

MLWVARLSGFLSAAMVMVVLSPSLQSFPPAEAIRSSQFDGSVRFPGQIAGGARGVAFRRAPSFRNAADCGAGAGNGTAANVCDPSLVHTAITLDEEYLRGSVAAVHSVVQHARCPESVFFHFLVSDPSLGDLVRAVFPQLRFKVYYFDPGRVRGLISTSVRQALEQPLNYARNYLADLLEPCVRRVIYLDSDLVLVDDVAKLWRTDLGGRTVGAPEYCHANFTKYFTSRFWSDQRFAGTFVGRRPCYFNTGVMVLDLERWRRAGYTQRIERWMEIQKSPPGRIYELGSLPPFLLVFAGHVAPIEHRWNQHGLGGDNVLGSCRDLHPGPVSLLHWSGSGKPWARLGAGRPCPLDALWAPFDLYGPAGAGGEESR*

>Zm1d047685MLWVARLSGLLSAAMVVVVLSPSLQSFPPAEAIRSSQFDGSVRFPGQIAGGARGVAFRRAPPFRNAADCGAGADNGTGANVCDPWLVHIAITLDNEYLRGSVAAVHSVVQHARCPESVFFHFLVSDPGLGDLVRAVFPQLRFKVYYLDPGRVRGLISTSVRQALEQPLNYARNYLAELLEPCVRRAIYLDSDLVVVDDVAKLWRTDLGGRTVGAPEYCHANFTKYFTGRFWSDQRFAGTFAGRRPCYFNTGVMVVDLERWRQAGYTQRIERWMEVQKSAAGRIYELGSLPPFLLVFAGHVAPIEHRWNQHGLGGDNVLGS

CRDLHPGPVSLLHWSGSGKPWARLGAGRPCPLDALWAPFDLYDPGGGAEESPYKF*

>Zm1d044752

MGGAAGEAMWAAVVAATVVFLAVEVSGAASALPRFAEAPEYRNGEGCPAAAAGVCDPGLVHIAMTLDTHYLRGSMAAVYSLLKHASCPESIFFHFLAAEAGAVDGADPEPELLRRAVAASFPSLRFEIYPFRAEAVAGLISASVRAALEAPLNYARNHLADLLPRCVPRAIYLDSDVLAADDVRRLWETRLPAAAVVAAPEYCHANFSRYFTPAFWSDPVLGARVFAGRRRPPCYFNTGVMVIDLRRWRAGNYRQRIERWMEIQKQKRIYELGSLPPFLLVFAGEVEAVDHRWNQHGLGGNNVHGSCRPLHAGPVSLMHWSGKGKPWDRLDAGRPCPLDHTWKSYDLYIPGDSGGAASPASGPALSASVFSW*

>Zm1d052292

MGAASPAMWAGLVLVALLAVSGPAVAAGLPRFAEAPEYRNGEGCPAPVAGAGVCDPGLVHIAMTLDAHYLRGSMAAIYSLLKHASCPESLFFHFLAAAEGGGAPAASGLRAVVAASFPSLSFEIYPFRADAVAGLISASVRAALEAPLNYARNHLAGLLPRCVPRAIYLDSDVLAVDDVRWLWETRLPAAAVVAAPEYCHANFSRYFTDAFWDDPVLGARVFAGRRRAPCYFNTGVMVIDLRRWRVGNYRQRIERWMEMQKEKRIYELGSLPPFLLVFAGEIEAVDHRWNQHGLGGDNVFGSCRPLHNGPVSLMHWSGKGKPWDRLDAGKPCPLDHTWKAYDLYIGENDSSSSGPSRSALSSLAACAATAAAASQLPPSAVQDSEINLSFTLPFTGDGSS

GSRPQECSRKGTYHLKLKPEKNIKAVF*

>Zm1d017984

MGAASPAMWAGLVLVVLLAASGPASAGLPRFAEAPEYRNGDGCPAPVTGAGVCDPGLVHIAMTLDAHYLRGSMAAIYSLLKHASCPESIFFHFLAAEGGGAPAVAELRAAVAASFPSLRFEIYPFRADAVAGLISASVRAALEAPLNYARNHLADLLPRCVPRAIYLDSDVLAVDDVRRLWETRLPAAAVVAAPEYCHANFSRYFTEAFWDDPVLGARVFAGRRRAPCYFNTGVMVIDLRRWRVGNYRQRIERWMEMQKEKRIYELGSLPPFLLVFAGEIEAVDHRWNQHGLGGDNVFGSCRPLHNGPVSLMHWSGKGKPWDRLDAGKPCPLDHTWKSYDLYIGENDSSPASGPSRSALSSLVAW*

>VIT_214s066g02350.1

MEANTMVCVRGFVLVLLLFLSPANAIRSFPSKVRDGVGESGIKSHVGLQFAEAPEYRNGPQCPISSGKEGLVSVCDPVLVHIAMTLDVEYLRGSVAAVHSVLRHASCPDNIFFHFIASDSNSMNPDDLSGIVRSVFPSLNFRVHVFNESLVKGLISSSIRRALDNPLNYARSYLADMLDGCVDRVIYLDSDVVVVDDIQKLWRTNLMGSRVIGAPVYCHANFTKYFSDKFWFDGELSGVFAGKKPCYFNTGVMVMDLGRWRGGDYTRRIEKWMEVQKERRIYELGSLPPFLLVFGGEVEGIDHRWNQHGLGGDNVVSSCRPLHPGPASLLHWSGKEKPWRRFDAGKPCPVDHLWAPYDLLRNRQQQDQLLISSYTSL*

>VIT_201s011g03050.1

MLPFPPLAIVLILSIAVHFPATVTSGDLPSFSEAPAFRNGEECPRTTWSSLPKGSYNPSIIHIAMTLDATYLRGSIAGVLSVLQHASCPENIVFHFLASHRRAELRRIIVTTFPYLSFHLYHFDTNLVKGKISSSIRRALDQPLNYARIYLADLLPGGVRRIIYFDSDLIVVDDVAKLWEINLGPHVLGAPEYCHANFTNYFTAKFWSNPAFTTSFRGRKPCYFNTGVMVIDLWRWREGKFTERLETWMRIQKRYRIYQLGSLPPFLLVFAGDVEGVEHRWNQHGLGGDNLEGLCRNLHPGPVSLLHWSGKGKPWLRLDSKRPCPLDSLWAPYDLFRHASLISDS*

>VIT_201s026g00980.1

MLGFGLNPVHLAVLAFILFSPVCLGIRSFPGRDVAGGDYGYEGFFRFAEAPDYRNGEECPAKGHKGYVSSCDPSLVHIAMTLDSEYLRGSIAAVHSILRHSSCPENVFFHFIAAEFDPASPRVLTQLVRSTFPSLNFKVYIFREDTVINLISSSIRSALENPLNYARNYLGDILDPCVERVIYIDSDLVVVDDIRKLWNITLTESRVIGAPEYCHAVFEKYFTDEFWSDSVLPRVFDSRKPCYFNTGVMVMDLVRWRKGNYRRKIENWMELQRRRRIYELGSLPPFLLVFAGNVEAIDHRWNQHGLGGDNVKGSCRPLHPGPVSLLHWSGKGKPWSRLDARKPCPVDHLWEPYDLYKPHRNHRLNHQQMLLSASSSTLVGLIRRNWLSTVVGGDYSFVEFFC*

>VIT_218s001g11860.1

MRTPPLLTLFFFFLVIYLFSLSFTAANATTLLSQHFREAPQFYNSPDCPSLIEEDNEDDDDASEEDSNGGYICSDLAVHVAMTLDTAYIRGSVAAILSVLQHSSCPQNVVFHFVASASSNASLLRATISTTFPYLRFQVYSFDDSAVAGLISTSIRSALDCPLNYARSYLANILPFCVRRVVYLDSDLVLVDDIGKLAATPLGDSSVLAAPEYCNANFTTYFTPTFWSNPSLSLTFANRKACYFNTGVMVIDLDRWRAGDYTSKIEDWMELQKRMRIYELGSLPPFLLVFAGNIVAVDHRWNQHGLGGDNFRGLCRDLHPGPVSLLHWSGKGKPWARLDANRPCPLDALWSPYDLLQTPFSLDS*

>VIT_207s005g01040.1

MDSSLSASSFLKGSFVPTASGLRYREKGRLIRSIFHFLSLKTLVDRRGTLGFLFSSLASLVTNPLFEFKSEKKVLRAEVYPLKMMWFMRFSGLLFAIMVMILFSPHFQSFPPAEAIRSSHHDGNLRVPILIPAGDSSRLFSFRKASAFNNAEECGFRDRNFSGKSSVCDPFLVHVAITLDVHYLRGSMAAVHSILQHSQCPEDIFFHFLVSETHLEILVRSTFPQLKFKVYYFNPEIVRNLISTSVREALEHPLNYARNYLADLLEPCVRRVIYLDSDLIVVDDIYKLWSTSLGTRTIGAPEYCHANFTRYFTDKFWSEKRYYGTFDGRKPCYFNTGVIVIDLAKWRRFGFTKRIERWMEVQKNNRIYELGSLPPYLLVFAGHVAPIEHRWNQHGLGGDNVKGSCRELHPGPVSLLHWSGSGKPWARLDMKAPCPIDAVWSPYDLYRPLR*

>VIT_207s104g00680.1

MALWSPPSSLPLLGLLSIIIFLRPTSVVTSPSGIRHGIIRKPSSYEPAVVLFREAPAFRNGDACGSSDADRIHVVMTLDANYLRGTIAALLSILQHSTCPENIDFHFLWSHFESDIFSSINSTFPFLNFKVYRFDSNRVRGKISKSIRRALDQPLNYARIYLGDILPADVRRVIYLDSDLVMVDDIAKLWGVELGDKVLAAPEYCHANFTNYFTSAFWSDRALARTFDGRKPCYFNTGVMVVDVEKWREGGYTKKVEEWMAVQKKKRIYQLGSLPPFLLVLAGNMQPVHHRWNQHGLGGDNLEGRCRSLHPGPISLLHWSGKGKPWLRLDSRRPCTVDHLWAPYDLYRSSTSSLEE*

>VIT_204s023g01120.1

MPATAPLLRLFLFLCGIIYFLAIHATAYQFREAPQFYNSPDCPSINYDDAEGYSHETSICSDDAVHVAMTLDAAYIRGSMAAILSVLQHATCPENVNFHFVASASADAHHLRRTIANSFPYLRFRVYRFDDSAVSGLISTSIRAALDCPLNYARNYLADLLPTCVRRVVYLDSDLVLVDDIAKLVATPLGDHSVLAAPEYCNANFTSYFTPTFWSNPSLSLTFAGRNACYFNTGVMVIDLQRWRAGDYTTKIVEWMELQKRMRIYELGSLPPFLLVFAGNIAPVDHKWNQHGLGGDNFRGLCRDLHPGPVSLLHWSGKGKPWARLDANRPCPLDALWAPYDLLKTPFALDN*

>VIT_205s020g02380.1

MAFWSSSMLPLLGLLSLFFLHPFSTSSSVHAIRLGAVVRRPSPDVPIFREAPAFRNGDACGTRDVDGIHVSMTLDANYLRGTMAAVLSILQHSTCPENLSFHFLSAQHVPEIVSTIQATFPYLNFRVYRFDSNRVRGKISKSIRRALDQPLNYARIYLADILPANVRRVIYLDSDLVMVDDISNLWGVDLGDKVVAAPEYCHANFTKYFTDEFWSSPEMAKTFKGRSPCYFNTGVMVVDVDRWRKGGYTQKVEEWMAVQKQNRIYDLGSLPPFLLVLAGNIKAVDHRWNQHGLGGDNLEGKCRNLHPGPISLLHWSGKGKPWLRLDSRKPCAVDHLWAPYDLYRSSRVSLEE*

>Thecc1EG041698t1

MPSSKPLNIFLLLTVTINLCFAELPSFREAPAFRNGGECPKTTWSSLDKKVHNPSLIHIAMTLDTAYLRGSIAGVFSVLHHATCPENIVFHFITTHRRGAELTRAITSTFPYLNFHLYHFDSNLVKGKISSSIRRALDQPLNYARMYLADLLPAGVRRIIYFDSDLIVVDDVNKLWSINLGSHVLGAPEYCHANFTNYFTSKFWSNPVFTASFKGRSRNPCYFNTGVMVIDLWKWREGKYTEKLENWMRIQKRYRIYELGSLPPFLLVFAGNVEGMEHRWNQHGLGGDNLEGLCRALHPGPVSLLHWSGKGKPWLRIDSKKPCPLDSLWAPYDLFRHPSLFSDS*

>Thecc1EG000465t1

MSKLALFPPHHLHRHHLLLVLLLFFLLASATNAAATTAYHRFKEAPQFYNSPSCRSLGSSGASDMCSDEGVHVAMTLDAAYLRGSMAAILSVLQHSSCPQNILFHFIASSTANHHHLRHKISHSFPSLKFQIYPYASSAVSGLISTSIRSALDCPLNYARNYLANILPPCLRRVVYLDSDLVLVDDIAKLAATPLGDNSVLAAPEYCNANFTSYFTPTFWSNPTLSLTFAGRKACYFNTGVMVIDMQRWREGDYTTKIIEWMELQKRMRIYELGSLPPFLLVFAGNIAPVDHRWNQHGLGGDNYRGLCRDLHPGPVSLLHWSGKGKPWVRLDANRPCPLDALWAPYDLLQAPFALDWRLCSRLIPIALFLFLSIILFRRRVSYLPNQESDFGASSHGSYRLVALETWFVLPCEKE*

>Thecc1EG004746t1

MLWIMRFSGFFSAAMVMIVLSPSLQSFPPAEAIRSSHLDSYLRLPSYQLSTSPHSLEDRFSFRKASEFRNADECGFTGHKITGVCDPSLVHVAITLDVEYLRGSIAAVHSILQHSLCPENIFFHFLVSETNLETLVRSTFPQLKFKVYYFDPEIVRNLISSSVRQALEQPLNYARNYLADLLEPCVRRVIYLDSDLVVVDDISKLWSTNLGSRTIGAPEYCHANFTKYFTGGFWSDQRLSGTFNGRKPCYFNTGVMVIDLVKWRRVGYTKRIERWMEIQKSDRIYELGSLPPFLLVFAGRVAPIEHRWNQHGLGGDNVRGSCRDLHPGPVSLLHWSGSGKPWLRLDSKKPCPLDALWAPYDLYGHSE*

>Thecc1EG004469t1

MQTTSMCTLKCPVFLFSLKLGIEYENPKWKPLSKWLPFSLLFYPNPIPTVPSSKPPIEGRMASLPLLGLLSLILTATAATATTTSATGIRHGIIRKPSSDVPVFREAPAFRNGDICGSNASDRIHIAMTLDANYLRGTMAAVLSILQHSTCPENVEFHFLWGKYEPEVLVSINSTFPYLNFRVYRFDPNRVRDKISKSIRQALDQPLNYARIYLADILPADVRRVLYLDSDLVVVDDILKLWEVDLEGKVLAAPEYCHANFTKYFTDLFWSDKGLASTFEGRHPCYFNTGVMVVDVDKWRQGGYTQKVEEWMAVQKQKRIYTLGSLPPFLLVLAGNIKAVNHRWNQHGLGGDNLEGRCRSLHPGPISLLHWSGKGKPWLRLDSRKPCVVDHLWAPYDLYNSSIQSLEE*

>Thecc1EG017924t1

MFVSRSVFGVIFLASLLLFPVNAIRLFTEKVTSSGGDETERESDLFMKFAEAPEYHNGPECPVLAEESLLCDPSVVHIAMTIDPEYLRGSTAAIHSVVKHSSCPENVFFHLIASDSSFVNANDLTQIVKSAFPSLSFKVYVFQENLVRNLISSSIRQALDSPLNYARSYLADIFEACVERVIYLDSDTIVVDDTQKLWRITLTGSRTIGAPEYCHANFAKYFTGEFWSDPELSRVFEGKRPCYFNTGVMVMDLARWREGDYTRKIERWMRIQKEKRIYKLGSLPPFLLVFGGDVEAIDHRWNQHGLGGDNLVNSCRSLHPGPVSLLHWSGRGKPWVRLDAGRPCPVDFLWAPYDLHK*

>Thecc1EG021073t1

MASWRTSTSIASLLGLLSFLLLHCSTTSTAIRVSTIIRNPSPMKLPIFREAPAFRNGDSCGSEEAYRIHIAMTLDANYLRGTMAAVLSMLQHSTCPENLSFHFLCAHSDSELVSSIKSTFPYLNFTIYRFDSNRVRGKISKSIRQALDQPLNYARIYLADILPADVKRVIYLDSDLVVVDDVGKLWGVDMEDNVLAAPEYCHANFTLYFNDAFWSDPVLSKTFQGRNPCYFNTGVMVVDVEKWRRGGYTKKVEQWMAFQKQKRIYHLGSLPPFLLVLAGNIKAVNHRWNQHGLGGDNFEGKCRNLHPGPISLLHWSGKGKPWLRLDSRKPCVVDHLWAPYDLYRSSRHFLED*

>Thecc1EG033980t1

MPRSPALFHFLLLSLFAVFFLANATAVSTMAQQFKEAPQFYNSLDCPVIVDEDEEGEGSILCSDQAVHVAMTLDKAYIRGSMAAILSVLQHSSCPQNIAFHFVASATANASLLRATISSSFPYLNFQVYPFDDSSVSRLISTSIRSALDCPLNYARSYLANLLPLCVRRVVYLDSDLVLVDDIAKLAATPLGDNSVLAAPEYCNANFTSYFTLTFWSNPSLSLTFANRKACYFNTGVMVIDLDRWRDGDYTTKIEEWMELQKRMRIYELGSLPPFLLVFAGNIVPVNHRWNQHGLGGDNFRGLCRDLHPGPVSLLHWSGKGKPWARLDANRPCPLDALWAPYDLLQTPFALDS*

>Thecc1EG010134t1

MRSILYAAAVLLVHFLLSARFCVGIRTISGGDGLGFGFSEAPDYRNGVECPVSVNTEVVSSCDPSLVHVAMTLDSEYLRGSIAAVHSVLRHASCPENIFFHFIAAEFDPASPRVLSKLVRSTFPSLNFKIYIFREDTVINLISSSIRQALENPLNYARNYLGDILDLCVDRVIYLDSDLVVVDDILKLWNTTLTNSRVIGAPEYCHANFTKYFTDGFWSDPVISRVFQSRKPCYFNTGVMVMDLVRWREGNYRKRIENWMEIQRKRRIYELGSLPPFLLVFAGNVEAIDHRWNQHGLGGDNVRGSCRSLHPGAVSLLHWSGKGKPWVRLDARKPCPLDHLWEPYDLYKGNLVKDQSSSSSSSSTGSSIFLGFSSYLL*

>Sobic.010G101400.1.p

MGGAAMWAAALAATVVFLAVEVSGAAGALPRFAEAPEYRNGEGCPAAAAGVCDPGLVHIAMTLDAHYLRGSMAAVYSLLKHASCPESIFFHFLAAEADGEEDPEPELLRRAVAASFPSLRFEIYPFRAEAVAGLISASVRAALEAPLNYARNHLADLLPRCVPRAIYLDSDVLAADDVRRLWETRLPAAAVVAAPEYCHANFSRYFTPAFWSDPELGARVFAGRRRPPCYFNTGVMVIDLRRWRAGNYRQRIERWMEIQKVKRIYELGSLPPFLLVFAGEVEAVDHRWNQHGLGGDNVHGSCRPLHAGPVSLMHWSGKGKPWDRLDAGRPCPLDHTWKSYDLYIPSDSSGAASPASGPALSASVLSW*

>Sobic.004G244100.1.p

MGAPSPAMWAGLVLVALLAASGPAATAAAGLPRFAEAPEYRNGEGCPAPVAGAGVCDPGLVHIAMTLDAHYLRGSMAAIYSLLKHASCPESLFFHFLAAEGGGAPAVADLRAAVSASFPSLRFEIYPFRADAVAGLISASVRAALEAPLNYARNHLADLLPRCVPRAIYLDSDVLAVDDVRRLWETRLPAAAVVAAPEYCHANFSRYFTEAFWNDPVLGARVFAGRRRAPCYFNTGVMVIDLRRWRVGNYRQRIERWMEMQKEKRIYELGSLPPFLLVFAGEIEAVDHRWNQHGLGGDNVFGSCRPLHNGPVSLMHWSGKGKPWDRLDAGKPCPLDHTWRSYDLYIGENDSSSASGPSRSALSSSAAW*

>Sobic.002G398400.1.p

MARACRMAPLLLLLVAVAASGIRVDVIRLPSSSSAPPPLPPSSPAFREAPAFRNGDECPPRGSPDGRVDVAMTLDANYLRGTMAAVFSILQHTACPENVAFHFLAAAGDYQHDSDPLAAIRATFPYLDPSVHRFDPSRVRGRISRSVRHALDQPLNYARIYLADTLPATVRRVIYLDSDVVVVDDVRKLWSVDLGDRHVVAAPEYCHANFTKYFTDAFWSDEELSAAFRGRRPCYFNTGVMVMDVARWRRGGYTRRVEEWMAVQKRKRIYHLGSLPPFLLVLAGDIRPVDHRWNQHGLGGDNVEGRCRSLHPGPISLLHWSGKGKPWLRLDARKPCTVDYLWAPYDLYKAAATALEE*

>Sobic.001G138200.1.p

MTRSPLTLILLLLLCAAAATAAVPRYREAPHFTNSAAAQCPPPLHPSDADAACSPHAAVHVAMTLDASYLRGTMAAVLSVLRHASCPESIHFHFIASASPKSRATAEELGATVRASFPSLAFRVYPFADEARVAGLISTSIRGALDRPLNYARSYLASTLPPCVRRVVYLDSDVVLTDDIASLAATPLPGEEETAVAAPQYCGANFTAYFTPGFWASPALSSTFAGRRACYFNTGVMVLDLARWRRAGYTAQIEEWMELQKRVRIYELGSLPPFLLVFAGRIASVDHRWNQHGLGGDNYRGLCRGLHAGAVSLLHWSGKGKPWDRLDAGRPCPLDAVWAKYDLLRPAAGIESS*

>Sobic.001G400100.1.p

MLWVARLSGFLSAAMVMVVLSPSLQSFPPAEAIRSSQFDGSVRFPGQIAGGARGIAFRRAPSFRNAADCGAGAGNGTAANVCDPSLVHIAITLDEEYLRGSVAAVHSVVQHARCPESVFFHFLVSDPGLGDLVRAVFPQLRFKVYYFDPERVRGLISTSVRQALEQPLNYARNYLADLLEPCVRRVIYLDSDLVLVDDVAKLWRTDLGGRTVGAPEYCHANFTKYFTGRFWSDQRFAGTFVGRRPCYFNTGVMVLDLERWRQAGYTQRIERWMEIQKSPPGRIYELGSLPPFLLVFAGHVAPIEHRWNQHGLGGDNVLGSCRDLHPGPVSLLHWSGSGKPWARLGAGRPCPLDALWAPFDLYGPAGAGESR*

>Sobic.001G364700.1.p

MPSNCAVVVVIAIFVVVVVILSASHAVTGARVGTMMIRQPSSVLMFREAPAFRNGPECDGDGRVDIAMTLDANYLRGTMAAVLSILQHTACPESVAFHFLTADADADDHVGLAAALRASFPFLDLRVYRFDPSRVRDRISRSVRQELDQPLNYARVYLADTLPPDVRRVTYLDSDVIVVDDVRTLASVDLAGHVVAAPEYCHANFSNYFTDAFWSHPALNGTFHGRRPCYFNTGVMVMDVDKWRAGGYTRRVEEWMAVQKRRRIYHLGSLPPFLLVFAGHIRAVDHRWNQHGLGGDNVEGRCRGLHPGPISLLHWSGKGKPWLRLDARRPCSVDYLWAPYDLYRYSSPVIDEW*

>Sobic.006G157800.1.p

MRVLAVVLLAAACAAAAGGGGELPEFREAPAFRNGAACAGAPTIHIAMTLDATYLRGSLAGVLSVLRHAACPESIAFHFVASSASPARRLDSLRRALAAAFPTLPATVHRFDARLVRGKISSSVRRALDQPLNYARIYLADLLPRSVSRVLYLDSDLLVVDDVARLWATDLGPDAALAAPEYCHANFTSYFTDTFWRHPEYAAVFANRTRVPCYFNTGVMVIDLDRWRSGGYTAKLEYWMEVQKQEARIYELGSLPPFLLVFAGEVKAVEHRWNQHGLGGDNVAGQCRELHPGPVSLLHWSGKGKPWLRLDAGRPCPLDALWAPYDLLRHRGARDDLLAAVA*

>Potri.010G038300.1.p

MVHFRLHSGVLLYTLVVLFPQLCLGIRSIPTRETNTGAVEVPNGFRFSEAPDYRNGRDCPVSTTNGRSVSSCDPSLVHIAMTLDSEYLRGSIAAVHSVLKHASCPESIFFHFVAAEFDPASPRVLTQLVRSTFPSLNFKVYIFREDTVINLISSSIRQALENPLNYARNYLGDMLDLCVDRVIYLDSDIVVVDDIHKLWNTALSGSRVIGAPEYCHANFTQYFTSVFWSDQVMSGTFSSARRKPCYFNTGVMVMDLVRWREGDYKRRIEKWMEIQKKTRIYELGSLPPFLLVFAGDVEAIDHRWNQHGLGGDNVRGSCRSLHPGPVSLLHWSGKGKPWVRLDAKKPCKLDHLWEPYDLYIIIELNDQSLTGFNSDSEPKRKKE*

>Potri.010G129400.1.p

MSLLSIFLFLNAVVFSAHGLSSAELPAFREAPAFRNGRECPKTTWLSSLNNYHDPSIIHIAMTLDATYLRGSVAGVLSVLQHAACPENVVFHFIATHRRADLRRTITSTFPYQTFHLYHFNTDLVKGKISSSIRRALDQPLNYARIYLADLLPMSVRRIIYFDSDLILVDDVAKLWNINLGAHVLGAPEYCHANFTNYFNSRFWSNSACAASLRGRRACYFNTGVMVIDLGKWREGKYTERLEYWMKVQKKYRIYELGSLPPFLLVFAGDVEGVGHRWNQHGLGGDNLEGLCRDLHPGPVSLLHWSGKGKPWLRLDSKRPCPLDYLWAPYDLYRHSSLFCDS*

>Potri.010G242300.1.p

MASCTFILPLGLLSFIIVANTVSSLPSSSGGIRLGVIRKPSADVPVFREAPAFRNGDSCGPLRIHIVMTLDANYLRGTMAAIFSILRHSTCPENMEFHFLWARFDREVFSSIKSTFPYLNFKFYRFDSNRVRGKISKSIRQSLDQPLNYARIYLADIIPSNVKRVIYLDSDLLLVDDIAKLWEVDLEDRVLAAPEYCHANFTYYFSNLFWLDPVLARTFHGRRPCYFNTGVMVVDVEKWRQVQLTQKVEGWMTVQKQKRIYHLGSLPPFLLVLAGNIKGVDHRWNQHGLGGDNMEGKCRSLHPGPISLLHWSGKGKPWLRLDSRKPCIVDHLWAPYDLYRSSLHVLEE*

>Potri.007G031700.1.p

MFKLGLKSPPPSSSPHHLLPILLLSIIIINSTATATANGKGFKEAPQFYNSPSCPSISTQDMCSQQAVHVAMTLDFPYLRGTMAAIFSILQHSSCPENIRFHFISSPSSTHLHETITSSFPYLRSQIYSFDTNPVSGLISTSIRSALDSPLNYARNYLANILPPCVPKVVYLDSDLVLVDDIASLAATPLGTGTVLAAPEYCNANFTTYFTPTFWANPMLSLTFSGRNACYFNTGVMVIDLERWREGDYTTKIVEWMELQKRMRIYELGSLPPFLLVFAGNIAAVDHKWNQHGLGGDNFRGLCRNLHPGPVSLLHWSGKGKPWVRLDANRPCPLDALWAPYDLLRTPFALES*

>Potri.002G200200.1.p

MLWILRFSGFFSAALVMIILSPSFQSFPPAEAIHSSNLDGHLRFPLLLSPADSLTQLSFRKSTIFRNADECGFSDHQSRGKTSVCYPSLVHVAITLDVEYLRGSVAAVHSILQHSMCPENVFFHFLVSETNLESLVRSTFPQLKFKVYYFDPEIVRSLISTSVRQALEQPLNYARNYLADLLEPCVKRVIYLDSDLVVVDDIAKLWTTNLGSRIIGAPEYCHANFTKYFTADFWSDKRFSGTFRGRKPCYFNTGVMVIDLVKWRWAGYTKRIERWMEIQKSHRIYELGSLPSYLLVFAGHVAPIEHRWNQHGLGGDNVRGSCRDLHPGPVSLLHWSGSGKPWLRLDSKQPCPLDALWAPYDLYGRPP*

>Potri.002G132900.1.p

MLMPRLIHKLTLLFLFLSLTTATITQQFKEAPQFYNSPDCHSIDQDGIDSDSESDGDKTIFCSEHAVHVAMTLDTAYIRGSMAAILSVLQHTSCPQNIAFHFVASASANTSLLRATISSSFPYLNFRVYTFDDSSVSRLISTSIRSALDCPLNYARSYLANIIPLCVRRVVYLDSDLVLVDDIAKLAATPLGEQSVLAAPEYCNANFTSYFTPTFWSNPSLSLTFADRKPCYFNTGVMVIDLDRWREGDYTTKIEEWMELQKRIRIYELGSLPPFMLVFAGDIVPVDHRWNQHGLGGDNFKGLCRDLHPGPASLLHWSGKGKPWARLDANRPCPLDALWAPYDLLQTPFALDC*

>Potri.005G128000.1.p

MYKPGRKLTLPSPPHLLLSLLLLLSIIIINATATATVNTKRFREAPQFYNSPTCPSISTKDMCSQQAVHVAMTLDFPYLRGTMAAILSILQHSSCPENTRFHFISSPSSTYLHETITSSFPYLRSQIHPFDTNSVSGLISTSIRSALDSPLNYARNYLANILPPCVRKAVYLDSDLVLVDDIAMLAATPLGTGTVLAAPEYCNANITAYFTPTFWANPSLSLTFSGRNACYFNTGVMIIDLERWREGDYTTKIVEWMELQKRMRIYELGSLPPFLLVFAGNIAAVDHKWNQHGLGGDNFRGLCRDLHPGPVSLLHWSGKGKPWVRLDENRPCPLDALWAPYDLMQTPFAIDF*

>Potri.014G125000.1.p

MLWILRFSGFFSAAVVMIILSPSIQSFPPAEAIRSSNLDGYLRFPILPSPPDYLPQLSFRRSTIFRNADECRFSARQIRGKTSVCDPSLVHIAITLDVEYLRGSIAAVHSILLNSLCPENVFFHFLVSETNLESLVRSTFPQLKFKVYYFDPEIVRSLISTSVRQALEQPLNYARNYLADLLETCVKRVIYLDSDLVVVDDIAKLWATNLGSRTIGAPEYCHANFTKYFTSGFWSDKRFSGAFRGRKPCYFNTGVMVIDLVKWRHAQYTKWIERWMEVQKSDRIYDLGSLPPYLLVFAGNVAPIEHRWNQHGLGGDNVRGSCRDLHPGPYSLLHWSGSGKPWLRLDSKQPCPLDFLWSPYDLYGHSRL*

>Potri.014G040300.1.p

MLMPRLIRKLTLLFTLLFLFLSLTTNAAATTTITQQFKEAPQFYNSPECPSIDQDEIDSEAEPDGDNTIFCSEHAVHVAMTLDAAYIRGSMAAILSVLQHTSCPQNIAFHFVASASANASLLRATISSSFPYLKFRAYTFDDSSVSGLISTSIRSALDCPLNYARSYLANILPLCVRRVVYLDSDLVLVDDIAKLAATPLGEKSVLAAPEYCNANFTSYFTPTFWSNPSLSLTFADRRPCYFNTGVMVIDLDRWREGDYTTKIEEWMELQKRMRIYELGSLPPFLLVFAGDIVPVDHKWNQHGLGGDNFRGLCRDLHPGPVSLLHWSGKGKPWARLDANRPCPLDALWAPYDLLQTPFALDS*

>Potri.008G018100.1.p

MASCASSLLLGLLSFLIVANIPSSSSSSGGIRLSIIRKPFAAVPVFREAPAFRNGDSCGLQRIHIVMTLDANYLRGTMAAVLSILQHSTCPENMEFHFLWSRFEREVFSSIKSTFPYLNFKFYRFDSNRVRGKISKSIRQALDQPLNYARIYLADIIPSDVKRVIYLDSDLVVVDDIAKLWEVDLEEKVLAAPEYCHANFTNYFSNLFWLDPVLAKTFHGRRPCYFNTGVMVVDVEKWRQGGITQKVEEWMTVQKQKRIYHLGSLPPFLLVLAGNIKGVDHRWNQHGLGGDNMEGKCRSLHPGPISLLHWSGKGKPWLRLDSRKPCIVDHLWAPYDLYRSSMHALEE*

>Potri.008G116900.1.p

MSILCIFLFLTTVVVSVQSLPNAELPAFREAPAFRNGRECPKKTWPSSFNNLNHHRHDPSIIHIAMTLDATYLRGSVAGVLSVLQHAACPEHIVFHFIATHRRADLRRTITSTFPYLTFHLYHFNTDLVRGKISSSIRRALDQPLNYARIYLADLLPFTVRRIIYFDSDLIVVDDVAKLWNINLGAHVLGAPEYCHVNFSYYFNSRFWSSPVYATSFTGRRACYFNTGVMVIDLRKWREGKYTEKLEYWMRVQKKNRIYELGSLPPFLLVFAGDVEGVEHRWNQHGLGGDNLEGLCRDLHPGPVSLLHWSGKGKPWLRLNSKRPCPLDSLWAPYDLYRHPTLFCDT*

>Potri.008G192600.1.p

MVHFRLHAGVLFFTVVVLFPLLCFGIRSIPSREGNSGAVEVLNGFRFAEAPEYRNGRDCPVLTSNGRLVSSCDPSLVHIAMTLDSEYLRGSIAAVHSVLKHASCPESIFFHFIAAEFDPASPRVLSQLVRSTFPSLNFKVYIFREDTVINLISSSIRQALENPLNYARNYLGDMLDLCVDRVIYLDSDVVVVDDIHKLWTTTLSGARVIGAPEYCHTNLTKYFTDVFWSDPVMSGTFTSARRKPCYFNTGVMVMDLVRWREGNYRGRIEKWMEVQRKTRIYELGSLPPFLLVFAGDVEAMDHQWNQHGLGGDNVRGTCRSLHPGPVSLLHWSGKGKPWVRLDAKRPCKVDHLWEPYDLFIRNNKGYSYSRNDHRRSSG*

>Os04g44850.1

MRVLAVALLAAAVLAAEAAAELPEFREAPAFRNGAGCAGAPTIHIAMTLDTTYLRGSLAGVLSVLRHAACPESIAFHFVASSASPARRLAALRRALAAAFPTLPATVHRFDARLVRGKISTSVRRALDQPLNYARIYLADLLPRSVSRVLYLDSDLLVVDDVARLWATDLGPDAALAAPEYCHANFTSYFTDAFWSHPEYSSIFTNRGRAPCYFNTGVMVIDLDRWRAGGYTVKLEYWMEVQKQEARIYELGSLPPFLLVFAGEVKAVEHRWNQHGLGGDNVAGQCRELHPGPVSLLHWSGKGKPWLRLDAGRPCPLDALWAPYDLLRRRGARDDLLAAVA*

>Os07g45260.1

MAPPTAVSASTAGLIWSLSPSLSLVVTTSVALLLVLGGVSGIRVNVIRLPSASPFPAFREAPAFRNGDGCPPARGSAAAEGGRVDVAMTLDANYLRGTMAAVFSILQHTACPESVAFHFLAARSDPDAGDLAAAIRATFPYLGAAVSVYRFDPSRVRGRISRSVRRALDQPLNYARVYLADTLPAGVRRVLYLDSDVVVVDDVRKLWSVDLAGHVVAAPEYCHANFTKYFTDAFWSDGELSGAAFRRGRRRPPCYFNTGVMVMDMGRWRDGGYTRRVEEWMAVQKRRRIYHLGSLPPFLLVLAGDIKAVDHRWNQHGLGGDNAEGKCRSLHPGPVSLLHWSGKGKPWLRLDSRKPCAVDYLWAPYDLYKAAVPALEE*

>Os03g47530.1

MDDMAKLAQMPRAPLLVLLLMLGVGAAVAVPEYREAPHFTNSAAARCPPPLPATDADAACSPHAAVHVAMTLDAPYLRGTMAAVLSVLRHASCPESVHFHFLASSSSSPEAAAAVRELRDTVRASFPSLAFRVYPFDESRVAGLISTSIRGALDRPLNYARSYLATTLPACVRRVVYLDSDVVVTDDIAALAATPLPGEAAVAAPEYCGANFTAYFTPGFWASRALSEAAFAGRRACYFNTGVMVLDLPRWRRAGYTAQIEEWMELQRRVRIYELGSLPPFLLVFAGRIAAVDHRWNQHGLGGDNYRGLCRGLHAGAVSLLHWSGKGKPWDRLDAGKPCPLDAVWAKYDLLRPAAAIETS*

>Os03g24510.1

MARCGSAIAAVAALLVLLGGHAAARIRVEHSGMVIRRPSSSIPSFREAPAFRNGEECGGGGRVDVAMTLDANYLRGTMAGVLSILQHTACPESVSFHFLAAGMDADLAAAVRATFPYLDLRVYRFDPSRVRGRISRSIRHALDQPLNYARIYLADTLPPDVRRVIYLDSDVVVVDDIRALASVDLGGHVVGAPEYCHANFTNYFTDAFWSDPALNGTFAGRRPCYFNTGVMVMDVGKWRAGGYTRRVERWMEVQKQTRIYHLGSLPPFLLVLAGDIQAVDHRWNQHGLGGDNVKGRCRGLHPGPISLLHWSGKGKPWIRLDARRPCAVDYLWAPYDLFRPSSPVLEE*

>Os03g18890.1

MLWAARVSGFFSAAMVMVVLSPSLQSFPPAEAIRSSQFDSHVRFPGQIAGGARGLAFRRAPAFRNAADCGNATGNVCDPSLVHIAITLDEEYLRGSVAAVHSVVQHATCPESVFFHFLVSDPALGDLVRAVFPQLQFKVYYFDPDRVRGLISTSVRQALEQPLNYARNYLADLLEPCVRRVIYLDSDLVVVDDVAKLWRTDLGGRTVGAPEYCHANFTKYFTDRFWSDKQFAGTFAGRRPCYFNTGVMVLDLARWRRTGYTRRIERWMEIQKSPAGRIYELGSLPPFLLVFAGHVAPIEHRWNQHGLGGDNVFGSCRDLHPGPVSLLHWSGSGKPWARLGAGRPCPLDALWAPFDLYGPADSAAEGSR*

>Os02g50600.1

MGGNPAMRAGLALVVVVVAVVVGDVGAALPRFAEAPEYRNGEGCPAAVAGAGVCDPGLVHIAMTLDAHYLRGSMAAIYSLLKHASCPESIFFHFLAAADGGEGCGGGVGELRTAVAASFPSLRFEIYPFRADAVTGLISASVRAALEAPLNYARNYLADLLPKCVPRAIYLDSDVLAVDDVRRLWETRLPAAAVVAAPEYCHANFSRYFTETFWSDPQLGDRVFAGRRRAPCYFNTGVMVIDLRRWRVGNYRRRIEVWMELQKEKRIYELGSLPPFLLVFAGEVEAVDHRWNQHGLGGDNVLGSCRPLHKGPVSLMHWSGKGKPWDRLDAGKPCPLDHTWKSYDLYIAEGDSSSASAPFALSSSALPAAAFSW*

>Os06g13760.1

MPARAAAAAAAVALLLAVCAQAAALPRFAEAPEYRNGEGCPAAATAAAGVCDAGLVHISMTLDAHYLRGSMAAVYSLLKHASCPESLFFHFLAEEEEVGGGGDLRRAVAASFPSLRFEIYAFRAEAVAGLISASVRAALESPLNYARNHLADLLPRCVPRAIYLDSDVLAVDDVRRLWETRLPAAAVVAAPEYCHANFSRYFTPAFWSDPGLGRRVFAGRRRPPCYFNTGVMVIDLRRWRAGNYRHRIERWMEIQKEKRIYELGSLPPFLLVFAGEVEAVDHRWNQHGLGGDNVRGSCRPLHDGPVSLMHWSGKGKPWDRLDAGNPCPLDHTWKSYDLYVAGDDGAAASSPASGPALSSTSTTWPALVFSW*

>Glyma14g08430.1

MKFNPKSKPPPHLLYFLIILSLSFFFPCVSSSRKQQFKEAPQFYNSPNCPSIEHHDILSSSEEAVHVAMTLDTTYIRGSMAAILSVLQHSSCPQNTFFHFVCSSNANANANTNTNASLLRATISNAFPYLNFQLYPFDDAVVSNLISTSIRAALDCPLNYARSYLPNLLPPRVKRVVYLDSDLVLVDDIAKLATTSLGQNSVLAAPEYCNANFTSYFTPTFWSNPSMSLTFAERKRKACYFNTGVMVIDLERWREGDYTRKIEEWMELQKRMRIYELGSLPPFLLVFAGNIVSVDHRWNQHGLGGDNFRGLCRDLHPGPVSLLHWSGKGKPWMRLDANRPCPLDALWAPYDLLKTSFSLDS*

>Glyma02g11100.1

MPPTLSLSLSLVLVVLFFAAVDAHFPATGELPTFREAPAFRNGRECRNRPRSDSVIHIAMTLDATYLRGSVAGVFSVLQHASCPENVVFHFIATTHRRTELRRIITATFPYLSFHLYHFDANLVRGKISYSIRRALDQPLNYARMYLADLLPATVRRIIYFDSDLIVVDDVAKLWSIDLHARVLGAPEYCHANFTNYFTHRFWSNPSYAASFKRRDACYFNTGVMVIDLWKWREGRYTEKLETWMRIQKRNRIYELGSLPPFLLVFAGDVERVEHRWNQHGLGGDNLEGLCRDLHPGPVSLLHWSGKGKPWLRIDSKKPCPLDSLWAPYDLFRHSPSLFSDS*

>Glyma02g01880.1

MLWLMRFSGFFSAAMLVILLSPSLQSFHPAEAIRSSHHLDGLLRLPPPRLSFRPAAPFRNAADGKCASSVPTSVCDPSLVHVAITLDVEYLRGSIAAVHSILQHSQCPENIFFHFLVSETNLESLVKSTFPQLNFKVYYFDPEIVRNLISTSVRQALEQPLNYARNYLADLLEPCVERVIYLDSDLVLVDDIAKLWSTSLGSRTIGAPEYCHANFTKYFTAGFWSDMRFASAFAGRRPCYFNTGVMVIDLVRWRKIGYSKRIERWMEIQKNDRIYELGSLPPFLLVFAGRVAPIEHRWNQHGLGGDNVKGSCRDLHAGPVSLLHWSGSGKPWTRLDSKHPCPLDALWAPYDLYGHAH*

>Glyma02g06640.1

MAPPSFFFVFFFILIFSCTSATTTQQQRFKEAPKFYNSPTCATLRHHPNPNHTCPDNAVHVAMTLDVSYLRGSMAAILSVLQHTSCPENVIFHFVTAASKSSSAAKLNQTLTTSFPYLNFQIYPFDDDAVSRLISTSIRSALDCPLNYARSYLSTLLPPCVAKIVYLDSDLILVDDISKLAETPLSGTAVLAAPEYCSANFSAYFTPSFWSNPSLSLVLANRRRPPCYFNTGVMVIDLRQWREGEYTTEIEEWMELQKRMRIYELGSLPPFLLVFAGRIAAVDHRWNQHGLGGDNFRGLCRDLHPGPVSLLHWSGKGKPWARLDAGRPCPLDALWAPYDLLETRFAALQA*

>Glyma02g03090.1

MLLLRQSAVVSSLILCFFFPPLLCLGIRSFPTTADDGAFFHYTEAPEYRNGAGCPVSSTRNFLPSCDPSLVHIAMTLDSGYLRGSIAAVHSVLRHSSCPENVFFHFIAAEFDPASPRVLTRLVRSIFPSLNFKVYIFREDTVINLISSSIRQALENPLNYARNYLGDMLDTCVSRVIYLDSDVVVVDDVGKLWRAAITHGRVIAAPEYCHANFTKYFTDEFWNDPLLSRVFNTREPCYFNTGVMVMDLAKWREGNYKRKIENWMELQRKKRIYELGSLPPFLLVFGGNVEAIDHRWNQHGLGGDNVNGVCRSLHPGPVSLLHWSGKGKPWVRLDEKKPCPLDRLWEPYDLYKQVKDSVRDQNWGFSSSILVGYAHDLL*

>Glyma06g03770.1

MKFKSRRPQPRALVAFFLILSIFSLPASSSSSSSSSIDDVKSSTIIHQFKEAPEFYNSPECASLTDNEEDSSDRYICSEEAVHVAMTLDTTYIRGSMAAILSVLQHSSCPQNTFFHFVCSSSASLLRAAISHSFPYLNFQLYTFDDSQVSGLISSSIRSALDCPLNYARSYLANLLPICVRRVVYLDSDLILVDDIAKLAATPLGENKVLAAPEYCNANFTSYFTPTFWSNPSLSLTFADRRPCYFNTGVMVIDLERWREGDYTTKIEEWMELQKRMRIYDLGSLPPFLLVFAGNIASVDHRWNQHGLGGDNFRGLCRDLHPGPVSLLHWSGKGKPWVRLDANRPCPLDALWAPYDLLNTPFSLDS*

>Glyma04g03690.1

MKFKSRRLEAEAGALVLFFLILSSSSSSNYAKPSTIIHQFKEAPEFYNSPECASLTHSSDSYICSEEAVHVAMTLDTTYIRGSMAAILSVIQHSSCPQNTFFHFVCSSSASLLRAAISHSFPYLNFHLYTFDDSQVSGLISTSIRSALDCPLNYARSYLPSLLPLCVRRVVYLDSDLILVDDIAKLAATPLGENTVLAAPEYCNANFTSYFTPTFWSNPSLSLTFADRRPCYFNTGVMVIDLERWREGDYTTKIQEWMELQKRMRIYDLGSLPPFLLVFAGNIASVDHRWNQHGLGGDNFRGLCRDLHPGPVSLLHWSGKGKPWVRLDANRPCPLDALWAPYDLLNTPFSLDS*

>Glyma17g36650.1

MKSNPKSKTPPHLLYYLLIILSLLFFFPCVSSTQQFKEAPQFYNSPNCPSIEHNICSEEAVHVAMTLDTTYIRGSMAAILSVLQHSSCPQNTFFHFVCSSNDNTNASLLRATISNTFPYLNFQLYPFHDAVVSGLISTSIRAALDCPLNYARSYLANLIPPCVKRVVYLDSDLVLVDDIAKLATTSLGENNNVLAAPEYCNANFTSYFTPTFWSNPSLSLTFADRKQKACYFNTGVMVIDLERWREGDYTRKIEEWMELQKRMRIYELGSLPPFLLVFAGNIVSVDHRWNQHGLGGDNFRGLCRDLHPGPVSLLHWSGKGKPWVRLDANRPCPLDALWAPYDLLRTPFSFDS*

>Glyma17g02330.1

MVFRSSTSLIGLLSLLFLLSVSSAIRLGLVRRPSPELPLFREAPAFRNGEDCGSSPSATINVAMTLDTNYLRGTMAAVLSMLQHSTCPENLAFHFLSSHDDPPELFSSILSTFPYLKMKIYPFDSNRVRGKISKSIRQALDQPLNYARIYLADTIPENVKRVIYLDSDLVVVDDIAKLYGVDMKGKVVAAPEYCHANFTLYFTDNFWSDPVLAKTFRGRKPCYFNTGVMVMDVDTWRKERYTEKVEEWMAVQKQQKRIYHLGSLPPFLLVLAGNIKAVDHRWNQHGLGGDNFEGKCRSLHPGPISLLHWSGKGKPWLRLDSRKPCIVDHLWAPYDLYRSSRHFFEE*

>Glyma13g04780.1

MFLSRSILFVFVFSACLLLIPANGIRSFARTNGYETEVEEVDPFAQFREAPEYRNQRKCTLIDTTNAQLVCDPSLVHVAMTIDWHYLRGSIAAVHSVVKHTSCPLNLFFHFIASDARLDSKDVFERIVHTSFPSLRFKVYVFRESLVDNLISPSIREALDNPLNYARSYLPDLLDQCIERVIYLDSDVIVVDDVQELWKVSLTGSRVIGAPEYCHANFTRYFSYEFWSSAEFSEVFQGKRPCYFNTGVMVMDLVRWRAGDYTRKIEKWMEIQKERRIYKLGSLPPFLLAFGGNVEAIEHRWNQHGLGGDNVRNSCRTLHPGPVSLLHWSGKGKPWTRLDAKMPCSVDFLWAPYDLYIPHHHTYHHYHHHQRIAIGSTHSSF*

>Glyma01g38520.1

MATSAAPKPNQYYFFTLILHFCLLLIISTSTTQQRFKQAPKFYNSPSCPTIRLSPTDTCSDEAVHVAMTLDVTYLRGSMAAILSVLQHSSCPENIIFHFVTAASSSLLNRTLSTSFPYLKFQIYPFDDAAAVSGLISTSIRSALDCPLNYARNYLANLLPSCVLKIVYLDSDLVLVDDIAKLAATPLGDNNNTVLAAPEYCNANFSAYFTPSFWSNPSLSLTFAGRTPCYFNTGVMVIHLQRWRAGDYTTKIQEWMELQKRMRIYELGSLPPFLLVFAGNIVPVDHRWNQHGLGGDNFRGLCRDLHPGPVSLLHWSGKGKPWARLDANRPCPLDALWAPYDLLLTPFALEA*

>Glyma01g04460.1

MLPLRLSAVVSSLILCFLFPPLLCLGIRSFPTTADDGAFFHYSEAPEYRNGAGCPVSSTRVSLPSWDPSLVHIAMTLDSGYLRGSIAAVNSVLRHSSCPENVFFHFIAAEFDPASPRVLTRLVGSIFPSLNFKVYIFREDTVINLISSSIRQALENPLNYARNYLGDMLDACVSRVIYLDSDVVVVDDVGKLWRAPITRERVIAAPEYCHANFTKYFTDEFWNDPLLSRVFSTRKPCYFNTGVMVMDLAKWREGNYRRKIENWMELQRKKRIYELGSLPPFLLVFGGNVEAIDHRWNQHGLGGDNLNGVCRSLHPGPVSLLHWSGKGKPWVRLDEKKPCPLDSLWEPYDLYKQVKDRVRDQNWGFSSSILVGYAHDLL*

>Glyma01g22480.1

MPPKLTLVLVVLFFAPVDAHFPATGELPTFREAPAFRNGRECRNRARSDSVIHIAMTLDATYLRGSVAGVFSVLRHASCPENIVFHFIGTTRRSTELRRIITATFPYLAFYLYQFDANLVRGKISYSIRRALDQPLNYARMYLADLLPATVRRIIYFDSDLIVVDDVAKLWSIDLHARVLGAPEYCHANFTNYFTHRFWSNPSYAASFKGRDACYFNTGVMVIDLWKWREGRYTEKLERWMRIQKRNRIYELGSLPPFLLVFAGDVERVEHRWNQHGLGGDNLEGLCRDLHPGPVSLLHWSGKGKPWLRIDSKKPCPLDSLWAPYDLFRHLPSLFSDS*

>Glyma19g01910.1

MFISRSILFVFVFSACLLLFPANGIRSFATTNGYETEVEEVDPFVQFREAPEYRNQQKCTLIDTTNVQLVCDPSLVHVAMTIDWHYLRGSIAAVHSVVKHTSCPQNLFFHFIASDARLESKDVFERIVHTSFPSLGFKVYVFRESLVGNLISPSIREALDNPLNYARSYLADLLDQCIERVIYLDSDVVVVDDVQELWKVSLTGSRVIGAPEYCHTNFTRYFSYEFWSSAEFSEVFQGKRPCYFNTGVMVMDLVRWREGGYTRKIEKWMEIQKERRIYKLGSLPPFLLAFGGDVEAIEHRWNQHGLGGDNVRNSCRTLHPGPVSLLHWSGKGKPWTRLDAKMPCSVDFLWAPYDLYIPHHHTHHQHHHHQRIAIGSTHSSF*

>Glyma19g40180.1

MFRITRFSGFFSAAMFVIVLVILSPSFQSEAIRSSHRFSFRKAPPFRNAAECGSISGETTTVCDPSLVHVAITLDVDYLRGSIAAVHSILHNSLCPENIFFHFLVSDTNLQTLVESTFPNLKFNVYYFDPNIVAHLISSSVRQALEQPLNYARNYLVDLLESCVERVIYLDSDLVVVDDVAKLWSASLDSRAIGAPEYCHANFTKYFTAGFWSEPRLSGTFAQRRACYFNTGVMVMDLVKWRKEGYTKRIERWMEIQKSDRIYELGSLPPFLLVFAGHVAPIEHRWNQHGLGGDNVKGSCRDLHPGPVSLLHWSGSGKPWIRLSSKRPCPLDSLWAPFDLYAHSSS*

>Glyma03g37560.1

MLRITRFSGFFSAAMFVTVLVILSPSFQSEAIRSSHRFSFRKAPPFRNAAECASVSGQTTTVCDPSLVHVAITLDVDYLRGSIAAVHSILHNSLCPENIFFHFLVSDTNLQTLVESTFPNLKFNVYFFDPNIVAHLISSSVRQALEQPLNYARNYLVDLLESCVERVIYLDSDLVVVDDVAKLWSASLDSRAIGAPEYCHANFTKYFTAGFWSESRLSGTFAQRRACYFNTGVMVMDLVKWRKEGYTKRIERWMEIQKSDRIYELGSLPPFLLVFAGHVAPIEHRWNQHGLGGDNVKGSCRDLHPGPVSLLHWSGSGKPWLRLSSKRPCPLDSLWAPFDLYTHPSS*

>Glyma10g01960.1

MLWLMRFSGFFSAAMLVILLSPSLQSFHPAEAIRSSHHLDGLLRLPPPRLSFRPAPRFRNAADANKCASSSVSTSVCDPSLVHVAITLDVEYLRGSIAAVHSILQHSQCPENIFFHFLVSETNLESLVKSTFPQLNFKVYYFDPEIVRNLISTSVRQALEQPLNYARNYLADLLEPCVERVIYLDSDLVVVDDIAKLWSTSLGSRTIGAPEYCHANFTKYFTAAFWSDTRFARAFAGRRPCYFNTGVMVIDLVRWRRIGYSKRIERWMEIQKNDRIYELGSLPPFLLVFAGHVAPIEHRWNQHGLGGDNVKGSCRDLHAGPVSLLHWSGSGKPWTRLDSKQPCPLDALWAPYDLYGHAH*

>Glyma07g38430.1

MVFRSSTSLIGLLSLLFLLLLLPAASSAIRLGLVRRPSPELPLFREAPAFRNGEECGSSPADTINVAMTLDANYLRGTMAAVLSILQHSTCPENLAFHFLSAHDDAPELFSSIRSTFPYLNMKIYRFDSNRVRGKISKSIRQALDQPLNYARIYLADTIPEDVKRVIYFDSDLVVVDDIAKLWGVDMEGKLVAAPEYCHANFTLYFTDNFWSDPVLAKTFEGRKPCYFNTGVMVMDVDTWRKERYTEKVEEWMAVQKQQKRIYHLGSLPPFLLVLAGNIKAVDHRWNQHGLGGDNFEGKCRSLHPGPISLLHWSGKGKPWLRLDSRKPCIVDHLWAPYDLYRSSRHFFEE*

>MD13G1046300

MPQGHGPLHLLLAALFLAATVQFPANSAAAELPEFREAPAFRNGRGCPRTAWSSLDQNSHYDPSIIHIAMTLDTTYLRGSVAGVFSVLRHATCPENIVFHFIAASHRSRRSSDLHHVITSTFPYLTFHLYHFDSNLVRGKISYSVRRALDQPLNYARIYLADLLPSGVRRIIYFDSDLIVVDDVARLWRIDLGRRVLGAPEYCHANFTNYFTPKFWSNAAFAAEFKGRSACYFNTGVMVIDLWKWREGKYTQKLEHWMRVQKRCRIYELGSLPPFLLVFAGDVEGVEHRWNQHGLGGDNVEGLCRDLHSGPVSLLHWSGKGKPWLRLDSKQPCPLDSLWAPYDLYRDHRHSSLFSDI*

>MD13G1178900

MALLSTPTPLPILLGLLSILLFHPITTTSGIRLGILRRPSPHLPVFREAPAFRNGEQCGSENTDAIHVAMTLDANYIRGTMAAVFSLLQHSTCPENLYFHFLSARIAPEFFSSIKSTFPYLNFKTYAFDSNRVRWKISKSIRQALDQPINYARIYLADILPTDMRRVIYLDSDLIVVDDIAKLWSVDMEDKVVAAPEYCRANFSQYFTDAFWSDPDLSKTFQGRNPCYFNTGVMVVDVDKWRKGGYTQKVEEWMALQKRKRLYHLGSLPPFLLVLAGNIKGVDHRWNQHGLGGDNFEGRCRNLHPGPISLLHWSGKGKPWLRLDARKPCTVDHLWAPYDLYHSARHFLEE*

>MD15G1269900

MLSFFISFILLFTTTTNAAVTTSERLREALATSTTQRFKEAPKFYNSPSCPDLNNHEHKVAVCSDEAVHVAMTLDAAYLRGSMAAILSVLQHSSCPENVIFHFVSSSSTSNSQLLQQTIAKSFPYLKFRVYPFDDSAVEGLISTSIRSALDCPLNYARNYLANILPTCVRHVVYLDSDIILVDDISKLASTPLGPTQVLAAPEYCNANFTSYFTASFWSNPTLSLTFENRKACYFNTGVMVIDLDKWRSGGYTDRIVEWMELQKRMRIYELGSLPPFLLVFAGNIAAVDHRWNQHGLGGDNFRGLCRDLHPGPVSLLHWSGKGKPWVRLDANRPCPLDALWAPYDLLQTPFALES*

>MD03G1271300

MATSSTSHGLLSLLLLIFLHCLTHGAATTGIRLGIIRKISPDVAIFQEAPAFRNGDVCEHAKKIHIAMTLDSNYLRGTMSAVLSILQHSTCPEIVEFHFLWARFEPEVLSNIKSTFPYLKFNVYRFDSKRVRGKISKSIRQALDQPLNYARIYLADIIPVDVNRVLYLDSDLVVVDDVAKLWKVDLQGKVLAAPEYCHANFTQYFTESFWSDNEMSKTFEGRNPCYFNTGVMVVDVDKWRQGGYTKKMEEWMLVQKQKRIYHLGSLPPFLLVLAGDITAVHHRWNQHGLGGDNLEGKCRSLHPGPISLLHWSGKGKPWLRLDSRRPCTVDHLWAPYDLYRSSTHYFEEREMMRSTVWEGQEGEK*

>MD16G1180100

MALLSTPTSLPILLGLLSILLFHPITTTSGIRLGFVPRPSPDLPVFREAPAFRNGEQCGSENADGIHVAMTLDANYIRGTMAAVFSLLQHSACPANLYFHFLSAHTTPKFFSSIKSTFPYLNFKTYAFDSDRVRGKISKSIRQALDRPMNYARIYLADILPNEMRRVIYLDSDLVVVDDIAKLWSVDMEDMVVAAPEYCRANFTEYFTNAFWCDQDLSKTFQGRNPCYFNTGVMVVDVDKWRKGGYTQKVEEWMALQKRKRLYHLGSLPPFLLLLAGNIKAVDHRWNQHGLGGDNFEGRCRSLHPGPISLLHWSGKGKPWLRLDSRKPCTVDHLWAPYDLYHSSRHFLEE*

>MD16G1048100

MPPGHGPLPLLLLLAALFATGTVQFLVNSAAVELPKFREAPAFRNGRGCPRTAWSSLDQHSHYDPSIIHIAMTLDATYLRGSVAGVFSVLQHATCPENIVFHFIAASHRNRRSSDLLRHAITSTFPYLTFHVYHFDSSLVRGKISYSVRRALDQPLNYARIYLADLLPSGVRRIIYFDSDLIVVDDVAKLWRIDLGRRVLGAPEYCHANFTNYFTPKFWSNAAFAAEFKGRRACYFNTGVMVIDLWKWREGKYTQKLEHWMRVQKRCRIYELGSLPPFLLVFAGDVEGVEHRWNQHGLGGDNVEGLCRDLHPGPVSLLHWSGKGKPWLRLDSKRPCPLDSLWAPYDLYRHSSLFSDS*

>MD02G1155400

MLSFLISFILLFTTTTNATITTSERLREALATATTQRFKEAPKFYNSPSCPDLNNHEHTAVAMCSDEAVHVAMTLDAAYLRGSMAAILSVLQHSSCPENVIFHFVSSSSTSNSQLLQQIIATSFPYLKFRVYPFDDSAVAGLISTSIRSALDCPLNYARNYLANILPTCVRRVVYLDSDLILVDDISKLASTPLGPTQVLAAPEYCNANFSSYFTPTFWSNPTLSLTFENRKACYFNTGVMVIDLDKWRSGGYTERIVEWMELQKRMRIYELGSLPPFLLVFAGNIAAVDHRWNQHGLGGDNFRGLCRDLHPGPVSLLHWSGKGKPWVRLDANRPCPLDALWGPYDLLQTPFALES*

>MD08G1036800

MPMPPPLLLLLLLLLFLSPATATNAAATTQQFREAPQFYNSQDCPSITTLPDVDVDPDESDDGHMICSPQAVHVAMTLDTAYIRGSMAAILSVLQHSSCPQNAVFHFVASAAANASLLRQTISTSFPYLKFRIYAFDDSHVSGLISTSIRSALDCPLNYADPTSPTFSPSASAAVAVLAAPEYCNANFTSYFTSAFWSNPSLSLTFADRHACYFNTGVMVIDLDRWRGDDYTAKIEEWMELQKRMRIYELGSLPPFLLVFAGKIAPVEHRWNQHGLGGDNFRGLCRDLHPGAVSLLHWSGKGKPWARLEANRPCPLDALWAPYDLLETPFVLDS*

>MD17G1042500

MLLLKPISLLLLLSLVLIPAAAIRSFPDPQTGKEEGFNMDFSEAPEYTNGISCPALLGSNDRAGKEVCDPSLVHVAMTIDSEYLRGTIAAVHSVFKHASCPENTFFHFVASDSSAVDSHHLSRILKSTFPSLNFRVYVFRESLVSHLISSSIRRALENPLNYARSYLADLLDPCVERVIYLDSDVVVVDDIQKLWEITLSGSRVIGAPEYCHANFTKYFSDEFWKDSELSKVFDQKRPCYFNTGVMVIDLARWRAGGFTRKIETWMEIQKEKRIYELGSLPPFLLVFGGDVEAIHHRWNQHGLGGDNLMNSCRSLHSGPVSLLHWSGRGKPWTRLDLGTPCPVDLLWAPYDLYKHHQSQLQDLNLLQHHHHQQFLML*

>MD11G1291900

MAVSSTSHGLLSLLLLILIHLLVFLHYPTHVAATTGIRLGIIRKPSPNVAIFREAPAFQNMDACEHAEKIHIAMTLDSNYLRGTMAAMLSILQHSMCPGIVEFHFLWARFDPEVLSNIQSTFPYLKFNVYRFDSNRVRGKISKSIRQALDQPLNYARIYLADILPVDVKRVLYLDSDLVVVDDVAKLWKVDLQGKVLAAPEYCHANFTQYFTESFWSDSEMSKTFEGRNPCYFNTGVMVVDVDKWRQGEYTKKMEEWMVVQKQKRIYHLGSLPPFLLVLAGDITAVHHRWNQHGLGGDNLEGKCRSLHPGPISLLHWSGKGKPWLRLDSRKPCTVDHLWAPYDLYRSSAHHFEE*

>Prupe.8G268500.1.p

MLWIIRFSGFFSAAMLMIVLSPSLQSFPPAEAIRSSHHHHHLDYSYLRLPPPVLLSDSGSMVGAGGRLNFRKASAFRNSNECASPSPKTQIGDACDPNLVHVAITLDVEYLRGSIAAVHSVLQHSLCPESVFFHFLVSETNLEALVQSTFPQLKFRVYYFDPRMVRSLISTSVRQALEQPLNYARNYLADLLETCVERVIYLDSDLVVVDDISRLWTTSLGSRTIGAPEYCHANFTNYFTPAFWSDKRLSGTFDCRKPCYFNTGVMVIDLIKWRRGRYTKRIEKWMEIQKNRRIYELGSLPPFLLVFAGHVAPIEHRWNQHGLGGDNVKGSCRDLHSGPVSLLHWSGSGKPWLRLDSKRPCPLDALWSPYDLYGHTK*

>Prupe.8G247500.1.p

MASAKTRGLLSLLLILPLITLATATAGIRLGIILKPSPDVAIFREAPAFRNGDACQPAEKIHIAMTLDSNYLRGTMGAVLSILQHSTCPEFIEFHFLWARFEPEVLSNIKSAFPYLKFNVYRFDSNRVRGKISKSIRQALDQPLNYARIYLADIIPVDVKRVLYLDSDLVVVDDVAKLWKVDLQGKVLAAPEYCHANFSKYFTESFWSDQMFSKTFEGRKPCYFNTGVMVVDVEKWRQGEYTKQMEEWMVVQKQKRIYHLGSLPPFLLVLAGDIRAVDHRWNQHGLGGDNLEGKCRSLHPGPISLLHWSGKGKPWLRLDSRKPCTVDHLWAPYDLYRSSAHYLEE*

>Prupe.7G148000.1.p

MLAFLINLILLFASSTNAATAASSGRLKEALATSTPQRFKEAPKFYNSATCPALNTHEHSSTVCSDVAVHVAMTLDAAYLRGSMAAILSVLQHSSCPENIIFHFVSSSSSSNSQALTQTIATSFPYLKFRVYPFNDSAVLGLISTSIRSALDCPLNYARNYLANLLPTCVRRVVYLDSDLILVDDISKLASTPLEPTQVLAAPEYCNANFTSYFTPSFWANPTLSLTFENRRACYFNTGVMVIDLDKWRAGGYTDRIVEWMELQKRMRIYELGSLPPFLLVFAGNIAAVDHQWNQHGLGGDNFRGLCRDLHPGPVSLLHWSGKGKPWVRLDANRPCPLDALWAPYDLLQTPFALEA*

>Prupe.3G276500.1.p

MGLRFSEAPEYTNGLSCPVLGLNLKSLALACNPSLVHVAMTIDSEYLRGTMAAVHSVLKHASCPDNTFFHFIASDSILVNLQDLTRIVQSTFPSLSFRVYVFRERLVNNLISSSIRQALDNPLNYARSYLADLLDPCVERVIYLDSDVIVVDDVQKLWKISLSGSRVIGAPEYCHANFTKYFSDGFWKDSELSKVFEGKKPCYFNTGVMVMDLVRWREGEYTKKIENWMAIQKERRIYELGSLPPFLLVFGGDVEAIHHRWNQHGLGGDNMVNSCRSLHPGPVSLLHWSGRGKPWSRLDLGMPCPVDLLWAPYDLYKHHHHHHLQDQHHLQHQHHHHQQTQQFSML*

>Prupe.1G384100.1.p

MPLPQPPPLLPLLLLVAFLILLLPAPSVSAATTSTSNATTTTTQQFREAPQFYNSPDCPSITPLHHDDLDPDESDDDHMMICAYQAVHVAMTLDTAYIRGSMAAILSVLQHSSCPQNAVFHFVASATSNASLLRDTISSSFPYLKFRIYPFDDSHVSGLISISIRSALDCPLNYARSYLADLLPLCVRRVVYLDSDLILVDDIAKLAATPLGPSSVLAAPEYCNANFTTYFTPAFWSNPSLSLTFADRKACYFNTGVMVIDLDRWRGGDYTTKIEEWMELQKRMRIYELGSLPPFLLVFAGNIAPVEHRWNQHGLGGDNFRGLCRDLHPGSVSLLHWSGKGKPWARLEANRPCPLDALWAPYDLLVTPFVLDS*

>Prupe.1G305600.1.p

MQNQSKYHLSPLSDSISRAPQHQSPIKYQNYPLTKTSLLSSPSRTPFHTSESNPHSNSLHHQTMPPDHGLLYFLLIVLVLTVTVKFPATSAAVQLPKFREAPAFRNGRGCPRTAWSSLDQHSHYNPSIIHIAMTLDTTYLRGSIAGVFSVLQHATCPENIVFHFIAASHHNRRPSDLLSHVITSTFPYLTFHLYHFDSNLVKGKISYSVRRALDQPLNYARIYLADLLPSSVRRIIYFDSDLIVVDDVEKLWSINLGRRVLGAPEYCHANFTNYFTPKFWSNAAFAASFKGRRACYFNTGVMVIDLWKWREGKYTEKLEHWMRVQKRCRIYELGSLPPFLLVFAGDVEGVEHRWNQHGLGGDNLEGLCRDLHPGPVSLLHWSGKGKPWLRLDSKRPCPLDSLWAPYDLYRHSSLFSDS*

>Prupe.1G147700.1.p

MASWSTSTPLPILLGLLSILLLHPLTTTFGIRLGIIHRRSPDLPVFREAPSFKNGEQCGSKNADGIHVAMTLDANYIRGTMAAVFSLLQHSTCPENLYFHFLSAHIAPEFFSSIKSTFPYLNFKTYAFDSNRVRGKISKSIRQALDQPMNYARIYLANILPPDMGRVIYLDSDLVVVDDIAKLWSVDMEDKVVAAPEYCQANFIQYFTNTFWSDPDLSKTFQGRNPCYFNTGVMVVDVDKWRKGGYTQKVEEWMALQKRKRLYNLGSLPPFLLVLAGNIKAVDHRWNQHGLGGDNFEGKCRSLHPGPISLLHWSGKGKPWLRLDSRKPCTVDHLWAPYDLYHSSRHFLEE*

>Prupe.1G262000.1.p

MLPFRLYAAVLFVLSISLLFPPFCIGIRSFPTRAIDGGDAFELASAAWARFSEAPDYRNGADCAVSMNKEMVSSCDPSLVHIAMTLDSEYLRGSVAAVHSVLKHASCPENVFFHFIAAEFDPASPRVLTQLVRSTFPSLNFKVYIFREDTVINLISSSIRQALENPLNYARNYLGDILGRCVDRVIYLDSDVLVVDDIHKLWNISLSGLRVIGAPEYCHANFTKYFTDGFWSDPVLSRVFSSRKPCYFNTGVMVMDLVRWRQGNYRKRIENWMELQRKRRIYDLGSLPPFLLVFAGNVEAIDHRWNQHGLGGDNVRGSCRSLHPGPVSLLHWSGKGKPWVRLESKNPCPLDHLWEPYDLYKPLRHHNAAKFQALSSISASTLIGFSSYLS*

>Sphfalx0002s0011.1.p

MPHTRMVWRMLLLPWPNARNGSIMVTIWPCIMPGFHRCSLGGLHTPAAKPVVVTQEKEEEAEENLSLSPSLPEVVLQQHQCPPHSHHSLHFWLTILPIALIFTILLASIAAQEYSFEVSSSSRTLNSKLHHHLFEPTVYISFAFGPTISSSWGFISGNIGSILKCRWCSFTARSRTQVALVRSSDAFQEDTMAANQTPMQLDRIACPAVAQAADADIFQSSGIKKVEGSMMMTEMVDQQPSGKDMTFCPRPNMMPSDSQTHGVFLQAQVKAEEEQELQLEGLFSREAGAHVTSRHEQSTKLKLPKFRKLTSVPDKTHYDDCSHKKVDCGSDLGSLCSDHFVHIAMTLDVNYLRGSMAAIYSILLHADCPSNIRFHFIATKGKEELENMVAESLPFLHFQTYSFNEESVKSLITHAVRHALEEPLNYARFYLAHMLDPCVKRIIYLDSDVLVLDRIEELWRTDMADSTVATPEYCHTNFTSYFSESFWTNFTLSSTFANKQPCYFNSGVMVINLEKWRENKCTARVEYWMEVQKEQQIYDLGSLPPLLLTFAGDIQPIDHRWNQHGLGGDTLKGDCRPSRNEPASLLHWSGGGKPWQRIDIHQPCPVDNIWAQYDLLEPGH*

>Sphfalx0031s0153.1.p

MVLHSRRTRGTATMIEILDMALIWALVVQALFSLATLDSAAAAAAAMRSYSQFDEQVEATTTSMSLGAAAGGGRGGGEQQPAAAAAHESSSEAQSLMTTTAAKPREILPSFREAPAFRNDNQCVQSPVVNRTAEGICDSSSVHIAMTLDIAYLRGSMAAVFSILKHTGCPENVIFHFLVGDRDDYDQNDHASSSSSSELLLRSLIDSTFPFLRFKIYHFNEALVKTRISASVRLALEHPLNYARSYLADILEPCVKRVIYLDSDLIVVDDIVKLWGTRLGFRAIGAPEYCHANLTKYFTDSFWNNKEMIKIFDGKKPCYFNTGVMVMNLVKWREQNYRGAIEDWMMKQQETRIYELGSLPPFLLVFAGDVEPIDHRWNKHGLGGDNLEGRCRPLHPGPVSLLHWSGKGKPWIRLDARKPCPVDNLWAPYDLLLPSSSAAASSSLSLSSSSYS*

>Sphfalx0098s0087.1.p

MVSTSLMRRRRVAAAAATRLEIWVLAAIALVLLQTQLFSASWSLVAVEAIRSQSRQFEQVVESSGSISVRLDHQQQTTAAAHALQLPESAGDHDQELQLVQQQQKIFLSGSASGGAAAAAAAGGKRQQKNLDHKITASSSLPHAFREAPAFRNDEQCVSVNRSKSSSSDSRICGDSNVVHIAMTLDMGYFRGSIAVVYSILKHAACPENVIFHFLAAAAAGDGNNDVTRDSDTAASLELLRSLIDSTFPFLVFKVYRFDESLVKSRISASVRVALEHPLNYARIYLGDILESCIKRVIYLDSDLIVVDDIVKLWDTRLEGSRVIGAPEYCHANLTNYFTDSFWNNKGLSSPIMSFKPGRSKSKPCYFNTGVMVMDLVKWRQQNYRATIENWMKRQKQSRIYELGSLPPFLLVFAGQVQPIDHRWNQHGLGGDNLEGRCRPLHPGPVSLLHWSGKGKPWMRLDAKHPCPVDSLWIPYDLLL

PPSSYS*

>Sphfalx0010s0142.1.p

MVRILKSTRRSAGIQIWIVVFLLLHAVLSWSLVVEARRYSQFEVVENPLTGQQQAQGRGAGEGDEEPAQQSPTSSSGKHKESWPLFREAPAFRNGNQCVSVHRRKSDHAEDVCDSSSVHIAMTLDVAYLRGSMAAVFSILKHTGCPENVIFHFLVSDRDAELSALIYSTFPFLRFKVYHFDEALVKSRISASVRLALEHPLNYARSYLADILEPCVKRVIYLDSDLIVVDDIVKLWGTRLGSHAIGAPEYCHANLTKYFTASFWNNEGLAKIFDGKRPCYFNTGVMVMDLVKWREQNYRATIEEWMESQKETRIYELGSLPPFLLVFAGDVEPIDHRWNQHGLGGDNLEGRCRSLHPGPVSLLHWSGKGKPWIRLDARKPCPVDNLWAPYDLLLPAASSYSS*

>Sphfalx0166s0029.1.p

MCDSSSVHIAMTLDVAYLRGSMAAVFSILKHSSCPENVIFHFLAGDRAAELRSLIFSTFPFLRFKVYHFDEELVKSRISPSVRLALEHPLNYARSYLADILEPCVERVIYLDSDLIVVDDIVKLWGTRLGSGSAIGAPEYCHTNFTKYFTDSFWNNKAFTKVFDHGRKQQQQQQKKKSKLCYFNTGVMVMNLVKWREQNYRSKIESWMERQKESRIYELGSLPPFLLLFAGDVEPIDHRWNQHGLGGDNLEGRCRALHPGPVSLLHWSGKGKPWIRLDARNPCPVDHLWAPYDLVLQSSTSVSSSSSYAS*

>Sphfalx0166s0023.1.p

MVRTLRTRGRAAAVLVALLLQALFWWTTVESIRFSQFAFDQKSSMGGGGGGDELLLQQQQEETTPTEVQKRPEDWPPFREAPAFKNDKQCVSAERKKKKNNGAEEEICDSSAVHIAMTLDVAYLRGSMAAVFSILKHSSCPENVIFHFLVGNRDAGLRSLIFSTFPFLRFKVYHFDEALVKSRISASVRLALEHPLNYARSYLADILEPCVERVIYLDSDLIVVDDIVKLWGTRLGSGAIGAPEYCHTNFTKYFTDSFWSNKAFTKVFNHRRNKPCYFNTGVMVMNLVKWREQNYRSTIESWMKRQKEARIYELGSLPPFLLVFAGDVEPIDHRWNQHGLGGDNLEGRCRPLHPGPVSLLHWSGKGKPWIRLDARKPCSVDNLWAPYDLHLPSMSSYL*

>Pp3c2_18670V3.1.p

MAPSATMWRLLPPWQNLHNGAIVSIWPCMPGFHRCSLGLRTPTCMLVDQPAAPDVAEYRQSHSTQLWLTTLPLAIVVTIFLVSIAAQDFNGVDTTSQTQPQFPAYRRVHTGIPFKTRVSTSWSITTSKLGHFFDVKRFHPKSLRSNQSASTRPVKIPEYESTTGQLLGVESVGEHPHPSVQRAAKSSSRSNIGLLPDLVSYSNEGLPLAQGQRQLKNQERFTKPSSTIEQLEKSQFPRFRKAVSLPNRTCSDASQCIDSNCDNEAQGSFCNERLVHIAMTLDINYLRGSMAAIYSILRHAECSGNIRFHFVATNGKEKLEKVVAETLPFLQFQTYPFDESLVKSRISYAVRHALEEPLNYARFYLAHMIDPCVKRIIYLDSDVLVIDRIEELWMINMGNSTVGTPEYCHANFHSYFTERFWRNSSLASIFANKKPCYFNSGVMLINLDRWRKEACTATLEYWMEVQKERHIYELGSLPPLLLTFAGSIQAIDSRWNQHGLGGDILRGDCRPTRNEPASLLHWSGGGKPWQRLDIHQPCPVDSIWAQYDLLEPSG*

>Pp3c5_28420V3.1.p

MVHRQGNRPLLTWPLIILSLLSLVSATRVSPFDFRVRPELHSDRSPEPQGPQSYAASESAWPSFREAPVFRNGRRCAFVNRTGNDTCDPNSVHIAMTLDVEYLRGSIAAIFSILKHTACPENVIFHFFAANRDEELRFLVCSIFPFLRFKVYHFDEALVNSRISPSVRPALDHPLNYARSYMSDILEPCIQRVIYLDSDLIVVDDIVKLWGTKLGPHAIGAPEYCHTNMTKYFTDAFWANRTLSRIFDGKKPCYFNTGVMVMDMTKWRIANYRAEIEHWMGVQSRTRIYELGSLPPFLLVFGGLVEPIDHRWNQHGLGGDNLEGKCRSLHPGPVSLLHWSGKGKPWIRIDQKKTCPVDSLWVPYDLLLSPLSYQ*

>Pp3c1_18380V3.1.p

MWRLLPPWLIFNNGSIVSFWPCIPGSHRYTLGLRTSMCTLVKQPPTTEVADCHHDRSTQLWLTALSFSIIFSIFLLSIAVQDFTVGDTTSKAQPQLPLNQRIYIGFSLKTRGSSSLNVIAEKLGHFFGFRRFNPNSFRSKQRASTTPVKIPSFGSALGKPMKTEPGGEHQQPSAQQATQSSSGFKTDLLPNLISNSNKGIPWLPSNANCGHEAQGSFCNESLVHIAMTLDANYLRGSMAAIYSILLHAECASNVRFHFVATKEKEELEKEPLNYARFYLAHMIDSCVKRIIYLDLDVLVLGRIEELWMTNMGNSTVGTPEYCHANFPSYFTENFWINSSLASTFANKQPCYFNSGMMLINLERWRKTRCTSTLEYWMEVQKQQHIYELGSLPPLLLTFAGSIQAIDNRWNQHGLGGDIVKGDCRPTRNEPAS*

>Pp3c25_14930V3.1.p

MVHRSVSTITSLSIILLTFSALATAVRPSPFDFPARAELHSEPALHSQGSGPQYSSGPEWPEFREAPAFRNGKQCAFVNRTENELDHICDRNSVHIAMTLDVKYLRGSMAAVFSILKHTACPENVIFHFFAADRDEQLRSLIFSTFPFLRFKVYHFDEALVNLRISPSVRPALEHPLNYARSYLADILEPCIQRVIYLDSDLIVVDDIVKLWGTRLGPYAIGAPEYCHTNMTKYFTNAFWQNRTLSRTFDGKKPCYFNTGVMVMDMTKWRTENYRAVIEQWMGVQNRTRIYDLGSLPPFLLVFGGSVEPIDHRWNQHGLGGDNLEGKCRPLHPGPVSLLHWSGKGKPWIRIDQRKTCPVDSLWAPYDLLQPSALSYQ*

>Pp3c16_25090V3.1.p

MVCRSVCTLITFSVVLLILSSLASAIRSSPFDSTARDGFYSEPALPPLGSGSQVSSGPAWPEFREAPAFRNGKQCAFVNRTQNELDHICDRNSVHISMTLDVKYLRGSMAAVFSILKHTACPENVIFHFFAADRDEQLRSLVFSTFPFLRFKVYHFNDALVNSRISPSVRPALEHPLNYARSYLADILEPCIQRVIYLDSDLIVVDDIVKLWGTKLGPHAIGAPEYCHTNVTKYFTDAFWNNRILSSTFDGKKPCYFNTGVMVMDMVKWRTENYRAVIEQWMAVQSSTRIYDLDSLPPFLLVFGGSVEPIDHRWNQHGLGGDNLEGKCRPLHPGPVSLLHWSGKGKPWIRIDQKRKCSVDSLWAPYDLLLPSTLSYQ*
