# Supplementary material for: Genome-Wide Study of the GATL Gene Family in Gossypium hirsutum L. Reveals that GhGATL Genes Act on Pectin Synthesis to Regulate Plant Growth and Fiber Elongation
Source: Genes (Basel). 2020 Jan 6;11(1):64. doi: 10.3390/genes11010064 (PMC7016653; doi:10.3390/genes11010064)
Supplement: Supplementary file 1 [file genes-11-00064-s001.zip › Supplementary Files/Table S2.docx]

**Supplementary Table 2. The GhGATL gene family between *G. hirsutum* (NAU, version 1.1) and *G. hirsutum* (HAU, version 1.1) database.**

| Gene name | Gene（NAU, version 1.1） | Chr | Gene （HAU, version 1.1） | Chr |
| --- | --- | --- | --- | --- |
| GhGATL1_At | Gh_A01G0957 | A01 | Gohir.A01G117500.1.p | A01 |
| GhGATL1_Dt | Gh_D01G1004 | D01 | Gohir.D01G105800.1.p | D01 |
| GhGATL2_Dt | Gh_D02G0934 | D02 | Gohir.D02G093600.1.p | D02 |
| GhGATL3_At | Gh_Sca005377G02 | - | Gohir.A02G162700.1.p | A02 |
| GhGATL3_Dt | Gh_D03G1673 | D03 | Gohir.D03G017600.1.p | D03 |
| GhGATL4_At | Gh_A05G1690 | A05 | Gohir.A05G196900.1.p | A05 |
| GhGATL4_Dt | Gh_D05G1880 | D05 | Gohir.D05G200000.1.p | D05 |
| GhGATL5_At | Gh_A05G2220 | A05 | - | - |
| GhGATL5_Dt | Gh_D05G2480 | D05 | Gohir.D05G262400.1.p | D05 |
| GhGATL6_At | Gh_A06G0103 | A06 | Gohir.A06G012400.1.p | A06 |
| GhGATL6_Dt | Gh_D06G0076 | D06 | Gohir.D06G009800.1.p | D06 |
| GhGATL7_At | Gh_A11G1728 | A11 | Gohir.A11G187100.1.p | A11 |
| GhGATL7_Dt | Gh_D11G1886 | D11 | Gohir.D11G193600.1.p | D11 |
| GhGATL8_At | Gh_A12G0617 | A12 | Gohir.D12G066700.1.p | A12 |
| GhGATL8_Dt | Gh_D12G0630 | D12 | Gohir.A12G066300.1.p | D12 |
| GhGATL9_At | Gh_A05G0764 | A05 | Gohir.A05G091000.1.p | A05 |
| GhGATL9_Dt | Gh_D05G0896 | D05 | Gohir.D05G092000.1.p | D05 |
| GhGATL10_At | Gh_A10G0951 | A10 | Gohir.A10G106100.1.p | A10 |
| GhGATL10_Dt | Gh_D10G1607 | D10 | Gohir.D10G162100.1.p | D10 |
| GhGATL11_At | Gh_A11G1098 | A11 | Gohir.A11G119700.1.p | A11 |
| GhGATL11_Dt | Gh_D11G1248 | D11 | Gohir.D11G124500.1.p | D11 |
| GhGATL12_At | Gh_A07G1155 | A07 | Gohir.A07G126900.1.p | A07 |
| GhGATL12_Dt | - | - | Gohir.D07G131000.1.p | D07 |
| GhGATL13_At | Gh_A11G1018 | A11 | Gohir.A11G112100.1.p | A11 |
| GhGATL13_Dt | Gh_D11G1174 | D11 | Gohir.D11G117200.1.p | D11 |
| GhGATL14_At | Gh_A12G1168 | A12 | Gohir.A12G127900.1.p | A12 |
| GhGATL14_Dt | Gh_D12G1288 | D12 | Gohir.D12G131600.1.p | D12 |
| GhGATL15_At | Gh_A03G1265 | A03 | Gohir.A03G144000.1.p | A03 |
| GhGATL15_Dt | Gh_D02G1704 | D02 | Gohir.D02G167200.1.p | D02 |
| GhGATL16_At | Gh_A05G3147 | A05 | Gohir.A05G362200.1.p | A05 |
| GhGATL16_Dt | Gh_D04G0487 | D04 | Gohir.D04G052200.1.p | D04 |
| GhGATL17_At | Gh_A05G2796 | A05 | - | - |
| GhGATL17_Dt | Gh_D05G3101 | D05 | Gohir.D05G321800.1.p | D05 |
