# Supplementary material for: Genome-Wide Study of the GATL Gene Family in Gossypium hirsutum L. Reveals that GhGATL Genes Act on Pectin Synthesis to Regulate Plant Growth and Fiber Elongation
Source: Genes (Basel). 2020 Jan 6;11(1):64. doi: 10.3390/genes11010064 (PMC7016653; doi:10.3390/genes11010064)
Supplement: Supplementary file 1 [file genes-11-00064-s001.zip › Supplementary Files/Table S3.docx]

**Supplementary Table 3. The *GATL* genes in *G.raimondii* and *G. arboreum.***

| Gene name | Gene | Gene name | Gene |
| --- | --- | --- | --- |
| GaGATL1 | Ga01G1462 | GrGATL1 | Gorai.002G1298 |
| - | - | GrGATL2 | Gorai.005G1061 |
| GaGATL3 | Ga02G0189 | GrGATL3 | Gorai.003G0179 |
| GaGATL4 | Ga05G2085 | GrGATL4 | Gorai.009G2053 |
| GaGATL5 | Ga05G2763 | GrGATL5 | Gorai.009G2743 |
| GaGATL6 | Ga06G0139 | GrGATL6 | Gorai.010G0127 |
| GaGATL7 | Ga11G2009 | GrGATL7 | Gorai.007G2070 |
| GaGATL8 | Ga12G2378 | GrGATL8 | Gorai.008G0700 |
| GaGATL9 | Ga05G0963 | GrGATL9 | Gorai.009G0945 |
| GaGATL10 | Ga10G1224 | GrGATL10 | Gorai.011G1812 |
| GaGATL11 | Ga11G2785 | GrGATL11 | Gorai.007G1334 |
| GaGATL12 | Ga07G1412 | GrGATL12 | Gorai.001G1421 |
| GaGATL13 | Ga11G2868 | GrGATL13 | Gorai.007G1256 |
| GaGATL14 | Ga12G1612 | GrGATL14 | Gorai.008G1428 |
| GaGATL15 | Ga03G1928 | GrGATL15 | Gorai.005G1876 |
| GaGATL16 | Ga04G1545 | GrGATL16 | Gorai.012G0584 |
| GaGATL17 | Ga05G3590 | GrGATL17 | Gorai.009G3453 |
