# Supplementary material for: Genome-Wide Study of the GATL Gene Family in Gossypium hirsutum L. Reveals that GhGATL Genes Act on Pectin Synthesis to Regulate Plant Growth and Fiber Elongation
Source: Genes (Basel). 2020 Jan 6;11(1):64. doi: 10.3390/genes11010064 (PMC7016653; doi:10.3390/genes11010064)
Supplement: Supplementary file 1 [file genes-11-00064-s001.zip › Supplementary Files/Table S4.docx]

**Supplementary Table 4. The *GhGATL* genes duplication types in *G. hirsutum*.**

| Gene name | Gene | Gene type | Chromosome |
| --- | --- | --- | --- |
| GhGATL1_At | Gh_A01G0957 | 4 | A01 |
| GhGATL1_Dt | Gh_D01G1004 | 4 | D01 |
| GhGATL2_Dt | Gh_D02G0934 | 4 | D02 |
| GhGATL3_At | Gh_Sca005377G02 | 1 | - |
| GhGATL3_Dt | Gh_D03G1673 | 1 | D03 |
| GhGATL4_Dt | Gh_D05G1880 | 4 | A05 |
| GhGATL4_At | Gh_A05G1690 | 4 | D05 |
| GhGATL5_Dt | Gh_D05G2480 | 4 | A05 |
| GhGATL5_At | Gh_A05G2220 | 4 | D05 |
| GhGATL6_Dt | Gh_D06G0076 | 4 | A06 |
| GhGATL6_At | Gh_A06G0103 | 4 | D06 |
| GhGATL7_Dt | Gh_D11G1886 | 4 | A11 |
| GhGATL7_At | Gh_A11G1728 | 4 | D11 |
| GhGATL8_Dt | Gh_D12G0630 | 4 | A12 |
| GhGATL8_At | Gh_A12G0617 | 4 | D12 |
| GhGATL9_At | Gh_A05G0764 | 1 | A05 |
| GhGATL9_Dt | Gh_D05G0896 | 1 | D05 |
| GhGATL10_Dt | Gh_D10G1607 | 3 | A10 |
| GhGATL10_At | Gh_A10G0951 | 1 | D10 |
| GhGATL11_Dt | Gh_D11G1248 | 1 | A11 |
| GhGATL11_At | Gh_A11G1098 | 1 | D11 |
| GhGATL12_At | Gh_A07G1155 | 4 | A07 |
| GhGATL12_Dt | Gohir.D07G131000.1.p | - | - |
| GhGATL13_Dt | Gh_D11G1174 | 4 | A11 |
| GhGATL13_At | Gh_A11G1018 | 4 | D11 |
| GhGATL14_At | Gh_A12G1168 | 4 | A12 |
| GhGATL14_Dt | Gh_D12G1288 | 4 | D12 |
| GhGATL15_Dt | Gh_D02G1704 | 4 | A03 |
| GhGATL15_At | Gh_A03G1265 | 4 | D02 |
| GhGATL16_Dt | Gh_D04G0487 | 1 | A05 |
| GhGATL16_At | Gh_A05G3147 | 1 | D04 |
| GhGATL17_At | Gh_A05G2796 | 4 | A05 |
| GhGATL17_Dt | Gh_D05G3101 | 4 | D05 |

0, 1, 2, 3, 4 stand for singleton, dispersed, proximal, tandem, segmental.
