# Supplementary material for: Genome-Wide Study of the GATL Gene Family in Gossypium hirsutum L. Reveals that GhGATL Genes Act on Pectin Synthesis to Regulate Plant Growth and Fiber Elongation
Source: Genes (Basel). 2020 Jan 6;11(1):64. doi: 10.3390/genes11010064 (PMC7016653; doi:10.3390/genes11010064)
Supplement: Supplementary file 1 [file genes-11-00064-s001.zip › Supplementary Files/Table S5.docx]

**Supplementary Table 5. The motifs consensus identified by MEME.**

| Motif | Consensus | Width | Nsite | E-value |
| --- | --- | --- | --- | --- |
| 1 | DHRWNQHGLGGDNFRGLCRDLHPGPVSLLHWSGKGKPWVRLDANRPCPLD | 50 | 33 | 1.9e-1475 |
| 2 | TFAGRKPCYFNTGVMVIDLDRWREGDYTKKIEEWM | 35 | 33 | 4.2e-1005 |
| 3 | ISSSIRQALDCPLNYARNYLADLLPPCVRRVIYLDSDLVLVDDIAKLWAT | 50 | 33 | 6.0e-1215 |
| 4 | SRVJAAPEYCHANFTSYFTPTFWSBPTLS | 29 | 33 | 2.1e-687 |
| 5 | VHVAMTLDVAYLRGSMAAVLSVLQHSSCPZNI | 32 | 29 | 4.4e-687 |
| 6 | QKRKRIYELGSLPPFLLVFAG | 21 | 33 | 3.5e-557 |
| 7 | ASDLRATISSTFPYLNFKIYPFDSSSVSG | 29 | 32 | 1.9e-376 |
