# Supplementary material for: Genome-Wide Study of the GATL Gene Family in Gossypium hirsutum L. Reveals that GhGATL Genes Act on Pectin Synthesis to Regulate Plant Growth and Fiber Elongation
Source: Genes (Basel). 2020 Jan 6;11(1):64. doi: 10.3390/genes11010064 (PMC7016653; doi:10.3390/genes11010064)
Supplement: Supplementary file 1 [file genes-11-00064-s001.zip › Supplementary Files/Table S6.docx]

**Supplementary Table 6. The degree of staining on Arabidopsis stem sections converted into gray values.**

|  | Area | Min | Max | IntDen |
| --- | --- | --- | --- | --- |
| WT | 204489 | 0.289 | 0.682 | 72894.41 |
| Ox-GhGATL2 | 236928 | 0.293 | 0.734 | 85924.08 |
| Ox-GhGATL9 | 228778 | 0.293 | 0.744 | 82985.12 |
| Ox-GhGATL12 | 280404 | 0.293 | 0.708 | 102480.2 |
| Ox-GhGATL15 | 230957 | 0.289 | 0.744 | 81821.04 |

Area: staining area; IntDen: total of gray values
