# Supplementary material for: Genome-Wide Study of the GATL Gene Family in Gossypium hirsutum L. Reveals that GhGATL Genes Act on Pectin Synthesis to Regulate Plant Growth and Fiber Elongation
Source: Genes (Basel). 2020 Jan 6;11(1):64. doi: 10.3390/genes11010064 (PMC7016653; doi:10.3390/genes11010064)
Supplement: Supplementary file 1 [file genes-11-00064-s001.zip › Supplementary Files/Table S7.docx]

**Supplementary Table 7. Sequences of primers.**

| Primer name | Sequence (5’-3’) | Application |
| --- | --- | --- |
| Gohir.D07G131000.1.p-F | ATGTTTCCTTCAAAGTCATTTC | Detection of differential genes between two version of *G. hirsutum* database |
| Gohir.D07G131000.1.p-R | TCAATGTGAGTGTCGGTACAA |  |
| Gh_A05G2220-F | ATGCCTAAATCACCATCACTCTTC |  |
| Gh_A05G2220-R | TCAAGAATCCAATGCAAACGG |  |
| Gh_A05G2796-F | ATGCGTTTTATTTTATATGCG |  |
| Gh_A05G2796-R | TCAAAACAAATAGCTGGGGTA |  |
| Ox-GhGATL2-F | ATGTCTAAACCACAGCACTATCATT | Amplified genes for transgenic Arabidopsis |
| Ox-GhGATL2-R | TGAATCCAATGCAAATGGAGT |  |
| Ox-GhGATL9-F | ATGCATCCATCTAAACCACTCA |  |
| Ox-GhGATL9-R | GCTATTGGAGAACAAAGACGG |  |
| Ox-GhGATL12-F | ATGCTTTGGGTTATGCAATTC |  |
| Ox-GhGATL12-R | ATGTGAGTGTCGGTACAAGTCG |  |
| Ox-GhGATL15-F | ATGCGTTCCATTTTCCATGCT |  |
| Ox-GhGATL15-R | TGACAAAAAACTGGAAAATCCCA |  |
| RT-GhGATL2-F | CGATTCAAGGAAGCCCCGTCAT | qPCR(Candidate genes) |
| RT-GhGATL2-R | TGTACCCCGAATGTAAGCAGCG |  |
| RT-GhGATL9-F | GATCTCGTCTTCCATCCGACGC |  |
| RT-GhGATL9-R | TCTTCCTTTAAACGACGCCGCA |  |
| RT-GhGATL12-F | ATCTACGAGCTGGGTTCGTTGC |  |
| RT-GhGATL12-R | CGGTACAAGTCGTAAGGTGCCC |  |
| RT-GhGATL15-F | CGAGGAGGCCGTGCTATTTCAA |  |
| RT-GhGATL15-R | CTAGCAGAAACGGAGGCAACGA |  |
| GhUBQ14-F | CAACGCTCCATCTTGTCCTT | qPCR (Internal control) |
| GhUBQ14-R | TGATCGTCTTTCCCGTAAGC |  |
| VI-GhGATL15-F | AAAGAAGTTGTTTCGTCGTGCGA | Amplification genes for VIGS |
| VI-GhGATL15-R | GGGTTTTCCAGGGCTTG |  |
